# Comparison of Neutralizing Dengue Virus B Cell Epitopes and Protective T Cell Epitopes With Those in Three Main Dengue Virus Vaccines

**DOI:** 10.3389/fimmu.2021.715136

**Published:** 2021-08-20

**Authors:** Josilene Ramos Pinheiro, Esther Camilo dos Reis, Rayane da Silva Oliveira Souza, Ana Luíza Silva Rocha, Lincoln Suesdek, Vasco Azevedo, Sandeep Tiwari, Beatriz Gonçalves Silva Rocha, Alexander Birbrair, Erick Carvalho Méndez, Wilson Barros Luiz, Jaime Henrique Amorim

**Affiliations:** ^1^Laboratório de Agentes Infecciosos e Vetores, Centro das Ciências Biológicas e da Saúde, Universidade Federal do Oeste da Bahia, Bahia, Brazil; ^2^Programa de Pós-graduação em Biologia e Biotecnologia de Microrganismos, Universidade Estadual de Santa Cruz, Bahia, Brazil; ^3^Laboratório de Parasitologia, Instituto, Butantan, São Paulo, Brazil; ^4^Departamento de Genética, Ecologia e Evolução, Universidade Federal de Minas Gerais, Belo Horizonte, Brazil; ^5^Departamento de Patologia, Universidade Federal de Minas Gerais, Belo Horizonte, Brazil

**Keywords:** dengue, vaccines, immunization programs, protection, immunoinformatics

## Abstract

The four serotypes of Dengue virus (DENV1-4) are arboviruses (arthropod-borne viruses) that belong to the *Flavivirus* genus, *Flaviviridae* family. They are the causative agents of an infectious disease called dengue, an important global public health problem with significant social-economic impact. Thus, the development of safe and effective dengue vaccines is a priority according to the World Health Organization. Only one anti-dengue vaccine has already been licensed in endemic countries and two formulations are under phase III clinical trials. In this study, we aimed to compare the main anti-dengue virus vaccines, DENGVAXIA^®^, LAV-TDV, and TAK-003, regarding their antigens and potential to protect. We studied the conservation of both, B and T cell epitopes involved in immunological control of DENV infection along with vaccine viruses and viral isolates. In addition, we assessed the population coverage of epitope sets contained in each vaccine formulation with regard to different human populations. As main results, we found that all three vaccines contain the main B cell epitopes involved in viral neutralization. Similarly, LAV-TDV and TAK-003 contain most of T cell epitopes involved in immunological protection, a finding not observed in DENGVAXIA^®^, which explains main limitations of the only licensed dengue vaccine. In summary, the levels of presence and absence of epitopes that are target for protective immune response in the three main anti-dengue virus vaccines are shown in this study. Our results suggest that investing in vaccines that contain the majority of epitopes involved in protective immunity (cellular and humoral arms) is an important issue to be considered.

## Introduction

Dengue virus (DENV) is an arbovirus (arthropod-borne virus) that belongs to the *Flaviviridae* family, *Flavivirus* genus. There are four distinct serotypes: DENV-1, DENV-2, DENV-3, and DENV-4 (1). They are responsible for promoting an infectious disease called dengue, which can be presented in a wide spectrum. This can range from subclinical disease to severe flu-like symptoms in those infected. Although less common, some people develop severe dengue, which can be any number of complications associated with severe bleeding, organ impairment and/or plasma leakage. Severe dengue has a higher risk of death when not managed appropriately (2). Several factors such as disordered urbanization, population growth, migration, international travels, and vector control challenges favor a rapid and efficient spread of the disease (3). Dengue is considered an important global public health problem with significant social-economic impact (4). According to the Pan American Health Organization (PAHO), more than 1.6 million dengue cases were registered until June 2020 in the Americas, 65% of them registered in Brazil (5).

DENV is a single-stranded positive sense RNA virus surrounded by an icosahedral capsid and an envelope. The RNA is translated shortly after the virus enters the host cell. It encodes three structural proteins: capsid protein (C), pre-membrane protein (pre-M), and the envelope glycoprotein (E). In addition, the genomic RNA also encodes seven non-structural (NS) proteins: NS1; NS2A; NS2B; NS3; NS4A, NS4B, and NS5, which participate both in the replication of the viral genome and in the assembly of the new viral particles (1). The E glycoprotein is involved in attachment of DENV to the host cell receptors as well as in membrane fusion. In addition, it is the main target for neutralizing and enhancing antibodies. Its ectodomain is arranged in three domains: DI, DII and DIII. DII is central in the monomers, surrounded by DI and DIII. The hydrophobic fusion loop is located in DII and is essential for membrane fusion. Domain III is frequently related to a receptor binding function (6, 7).

Controlling dengue by suppressing mosquito vectors is only effective if applied before the circulation of viruses in a certain region. Due to epidemiological complexity of the disease, once it is established, agencies responsible for disease prevention face significant technical, scientific and operational difficulties in controlling dengue (8). Thus, according to the World Health Organization, the development of a safe and effective dengue vaccine is a priority. To date, one anti-dengue virus vaccine has already been licensed in endemic countries (Mexico, Philippines, Brazil, El Salvador, Costa Rica, Paraguay, Guatemala, Peru, Indonesia, Thailand, and Singapore) (9), while the other six vaccine formulations are under development; two are in phase III of clinical trials and four are in the early stages of clinical testing in different regions worldwide. A recently published review comprehensively explores details regarding development and clinical trials of anti-dengue virus vaccine formulations (9).

Both, antibodies and T lymphocytes play important roles in fighting DENV (10). Antibodies prevent host cells from being infected mainly through neutralization mechanisms. CD4+ T lymphocytes control viral replication mainly by producing inflammatory and antiviral cytokines, and CD8+ T lymphocytes contribute to controlling DENV spread through direct cytotoxicity on infected cells (9, 11). In addition, the production of IFN-γ by both CD4+ and CD8+ T cells is a hallmark of protective immunity (10, 12–15). However, the immune response against DENV is not that straightforward and can lead to exacerbated forms of the disease in the case of sequential contacts with different viral serotypes. In general, it occurs due to a phenomenon called antibody-dependent enhancement (ADE), in which antibodies generated against a first DENV serotype bind less efficiently to a different serotype and promote increased viral entry and replication in host cells through Fc-γ receptors (9, 16). In addition, exacerbation of DENV infection can also occur due to cross-reaction mediated by T lymphocytes. In both cases, an original antigenic sin leading to cytokine storms occurs (17, 18). There is a serious risk of vaccines inducing such mechanisms if the immune response is not balanced against all DENV serotypes. Therefore, an ideal anti-dengue vaccine should generate immune responses with antiviral mechanisms, as well as induce a safe, long-lasting, and balanced immune response for all four DENV serotypes, therefore reducing the risk of exacerbated inflammatory responses related to ADE and T cells cross-reaction.

In this study, we aimed to compare the main anti-dengue vaccine antigens regarding their compositions and potential to protect. We studied the licensed DENGVAXIA^®^ from Sanofi Pasteur; LAV-TDV from NIAID/Butantan, which is under phase III of the clinical trial; and TAK-003 from Takeda, which concluded phase III of the clinical trial. Dengvaxia^®^ is a live, attenuated and tetravalent recombinant vaccine. Its antigens consist of chimeric viruses in which genomic sequences encoding the pre-membrane (prM) protein and the envelope glycoprotein (E) of the 17D strain of *Yellow fever virus* (YFV) were replaced by those of each of the four serotypes of DENV. LAV-TDV is a live, attenuated and tetravalent vaccine solely based on DENV. The viruses were attenuated by deletions in their genomes and one of them consists of a chimera of a DENV2 and a DENV4. And TAK-003 is also a live attenuated tetravalent vaccine that is based on DENV2 (16681) PDK 53, which was used as a backbone in the constructs of the chimeric vaccine viruses of DENV1, DENV3 and DENV4 (9).The levels of presence and absence of epitopes that are target for protective immune response in the three main anti-dengue virus vaccines are shown here.

## Methods

### Reconstruction of Sequences Used in Vaccine Antigens

We performed a search for anti-dengue vaccine formulations both, in clinical trial records (ClinicalTrials.gov) and papers obtained from PUBMED (https://www. ncbi.nlm.nih.gov/pubmed/). The search filters used at ClinicalTrials.gov were as follows: Condition or disease: “dengue”; type of study: “interventional clinical trials”; study results: “with result”; Eligibility criteria: “child (birth–17)”, “adult (18–64)”, “elderly (65+)”; Gender: “all”; Study phase: I, II, III and IV. Pubmed searches were performed using keywords: dengue, vaccine, clinical trial. We selected three vaccine formulations that were either licensed or were under phase III of the clinical trial or had finished phase III of the clinical trial: DENGVAXIA® from Sanofi Pasteur; LAV-TDV from NIAID/Butantan Institute and TAK-003, from Takeda.

Nucleotide sequences of vaccine antigens were rebuilt using data recovered from the nucleotide database of the National Center for Biotechnology Information (NCBI) platform (https://www.ncbi.nlm.nih.gov/nucleotide/). The assembly was performed using the software ApE-A plasmid Editor, and the translation from DNA to protein sequence was performed using the Translate tool (https://web.expasy.org/translate/). Then, the sequences were used to compose a dataset in FASTA format (**Supplementary Data**). The dataset was used for the further analyses.

### Construction of a Dataset of Circulating DENV 1-4 Sequences

The DENV 1-4 polyprotein sequences dataset was built by applying the same filters for *Flavivirus* previously described (19). Sequences were retrieved until January 2021. The dataset consisted of 189 DENV polyprotein sequences: 60 for DENV1, 58 for DENV2, 46 for DENV3, and 25 for DENV4, representing five regions of the world: The Americas, Europe, Asia, Africa, and Oceania (**Supplementary Table 1**).

### Survey of Epitopes Involved in Protective Immunity Against Dengue

For B cell epitopes, we carried out a search at IEDB (Immune epitope database) (https://www.iedb.org/) for B cell epitopes validated in neutralization assays using the following filters: “any epitope”, “dengue1-4”, “B-cell positive essays”, “any MHC restriction”, “human host”, “infectious diseases” and “only positive B-cell assays with neutralization essays”. We retrieved 50 B cell discontinuous epitopes from IEDB, namely, 20 from DENV1, 13 from DENV2, 13 from DENV3, and 4 from DENV4 (see **Supplementary Table 2**).

Additionally, we carried out a bibliography search for the studies about the protective anti-dengue response induced by T lymphocyte epitopes that were experimentally characterized. The data was retrieved from Pubmed database (https://www.ncbi.nlm.nih.gov/pubmed/) using the following search criteria: i) studies evaluating the survival or viral load control in challenge experiments, either using human or humanized animal models (transgenic animals expressing HLA); ii) infection of human host by DENV without presentation of pathological signs; iii) identification of important epitopes in the context of an immune response induced by anti-dengue vaccines known to be protective. We constrained our search to studies that contained epitope binding to HLA (human leukocyte antigen) and analysis of the proinflammatory cytokine production profile by CD4+ T cells or cytotoxicity mediated by CD8+ T cells. We also selected only studies in which at least two different methods were used to validate the secretion of pro-inflammatory cytokines or cytotoxic activity. The keywords used were: CD4+ T lymphocyte, CD8+ T lymphocyte, epitopes, protection, immunity, Dengue virus.

A total of 175 epitopes were retrieved from five articles (see **Supplementary Table 3**). However, eight of them were found more than once and, therefore, repeated epitopes were removed from the dataset for the further analyses.

### Conservation Analysis

The IEDB conservation analysis tool (http://tools.iedb.org/conservancy) was performed as previously described (20). Briefly, we computed the conservation of B cell epitopes retrieved from the IEDB database and T cell epitopes characterized and reported in the literature. Only epitopes 100% conserved were considered.

### Population Coverage Analysis

The T cell epitopes selected in the conservation analysis were subjected to population coverage analysis using the IEDB population coverage calculation tool (http://tools.immuneepitope.org/tools/population/iedb_input), as previously described (20).

### Structural Biology Analysis

Epitopes conserved in vaccines were mapped in a 3D envelope glycoprotein model, as previously described (20). The PyMol program (http://www.pymol.org/) was used to perform the epitope mapping. The 3D model protein was retrieved from the protein database (https://www.rcsb.org/).

### Statistical Analysis

Statistical analysis was performed to compare the results of the epitope conservation scores that were present in at least one of the three vaccines. Scores were defined based on the levels of conservation of epitopes present in vaccines in circulating viruses. This method is shown in details in **Supplemental Material**. Comparisons were performed regarding location of epitopes in specific viral proteins and percentage of conservation in circulating viruses (clinical isolates). The Kruskal-Wallis one-way test was used to verify whether the differences in the medians of the set of epitopes present in each protein were significant. This statistical analysis was only necessary for the comparison of the conservation values among the T cell epitopes. We did not compare conservation regarding B cell epitopes because they do not vary in relevant levels.

## Results

### Reconstruction of the Sequences Used in Vaccine Antigens

**Figure 1:**
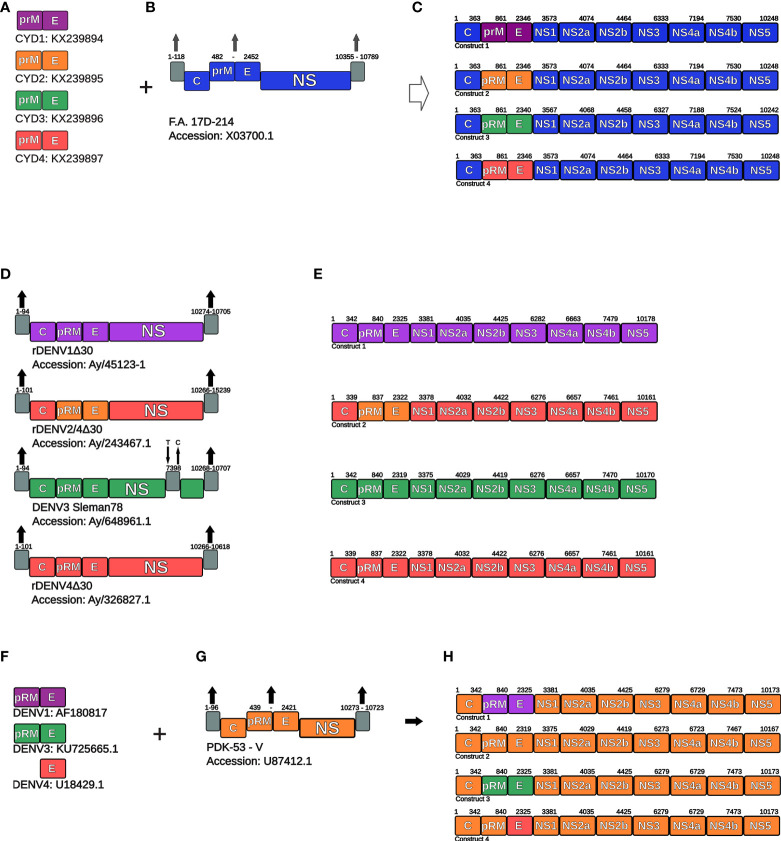
Schematic representation of genetic constructions of vaccine antigens. **(A)** PrM and E coding sequences were used to chimerize *Yellow fever virus* **(B)** and generate DENGVAXIA^®^ vaccine antigens (chimeric viruses) **(C)**. **(C)** Schematic representation of the final constructs of the DENV1-4 vaccine viruses of the DENGVAXIA^®^ formulation. **(D)** Representation of genomic characteristics of vaccine viruses of the LAV-TDV vaccine formulation **(E)** Schematic representation of the final constructs of the DENV1-4 vaccine viruses of the LAV-TDV formulation. **(F)** Representation of coding sequences of PrM and E proteins of the DENV1 and DENV3, and E protein of DENV4. These sequences were used to modify PDK-53-V infectious clone **(G)** and generate vaccine viruses of the TAK-003 vaccine formulation **(H)**. Known accession numbers are shown. NS, non-structural proteins.

In order to assemble the components of the DENGVAXIA^®^ vaccine, the sequences used for genetic recombination were extracted from GenBank, according to the accession numbers in **Figures 1A, B**. The vaccine constructs were assembled in the ApE-A plasmid Editor. The sequences from prM and E proteins (nucleotides 482-2452) from the YFV 17D backbone as well as the nucleotides at the 5 ‘end (1-118) and 3’ end (10355-10789) were removed from the assembly because they are not present or are not translated in vaccine antigens. Finally, the sequences of each Chimeric yellow fever virus-DENV (CYD) were inserted, and the four constructs were assembled (see **Figures 1A–C**), translated using the Translate tool, and deposited in FASTA format (see **Supplementary Data**).

The sequences of the vaccine antigens rDEN1Δ30, rDEN2/4Δ30, and rDEN4Δ30 that compose LAV-TDV were retrieved from the NCBI Database (Genbank) according to the accession numbers in **Figure 1D**. We did not obtain the accession number for DENV3 antigen, therefore, we used the sequence obtained from the Sleman/78 DENV strain, accession number: AY648961.1 (21), and applied the 7398 nucleotide point mutation from Cytosine to Thymine. This alteration contributes to viral attenuation, as previously described (21). For all sequences, we removed the 5´ and 3´ untranslated regions (UTR), which do not participate in polyprotein translation. The nucleotide position deletions in viral genomes are as follows: for DENV1 (1-94 and 10274-10705), DENV2 (1-101 and 10266-15239), DENV3 (1-94 and 10268-10707), and DENV4 (1-101 and 10266-10618) (see **Figures 1D, E**). Finally, the vaccine sequences were translated and used to compose a sequence set in FASTA format (see **Supplementary Data**).

TAK-003 is a tetravalent vaccine. Its antigens are based on the genetic backbone of the DENV-2 PDK-53-V variant, as previously described (22). In order to build the chimeras, we used the nucleotide sequence from PDK-53-V, according to the accession number in **Figure 1G**. The PDK-53-V virus is not annotated, then we used the PDK-53-E virus sequence (accession number M84728.1) (23) as a reference for annotation. The prM and E sequences (nucleotides 439-2421) and the UTR sequences (nucleotides 1-96 and 10273-10723) from PDK-53-V were removed for insertion of DENV1, DENV3, and DENV4 prM and E coding sequences, as shown in **Figures 1F–H**. For DENV4, only the E protein-coding sequence (Accession number: U18429.1) was found using the Genbank filter: “dengue virus 4 strain 1036 Indonesia 1976”. Therefore, the PrM coding-sequence of the PDK-53-V virus was used in the DENV4 vaccine. Finally, the nucleotide sequences of vaccine viruses were translated and deposited in a sequence dataset in FASTA format (see **Supplementary Data**).

### Conservation Analysis and Structural Biology of B Cell Epitopes Targeted by Neutralizing Antibodies

In the IEDB search, we retrieved 50 discontinuous IEDB B cell epitopes using the following filters: “any epitope”, “dengue1-4”, “positive assays for B cells”, “any MHC restriction”, “human host”, “infectious diseases” and “only positive assays for B cells with neutralization assays”. It is important to highlight that such epitopes are target for neutralizing antibodies which were previously reported. We evaluated the conservation of these 50 epitopes in vaccine antigens from the three manufacturers. However, we did not find relevant differences in the number of epitopes conserved among the vaccine antigens studied here (see **Table 1**). From 22 epitopes present in the LAV-TDV vaccine, only one is absent in TAK-003 vaccine (ID: 504135, sequence: F585, S586, I587, D588, K589, E590, M591, A592, E593, T594, T599, V600, V601, K602, V603, K604, E606, N641, V643, T644,N645, I646, L668, H669, W670, G678, K679) and another is absent in DENGVAXIA^®^ (ID: 504136, sequence: F586, K587, L588, E589, K590, E591, V592, A593, E594, T595, G598, T599, V600, L601, V602, Q603, V604, K605, E642, I647, E648, S670, F672, K674, G675, S676, S677, I678, G679, K680). Both epitopes are located in the envelope glycoprotein in the LAV-TDV vaccine. It is important to highlight that all of these conserved epitopes are discontinuous. In addition, most of them are located in the envelope glycoprotein and only one of them is located in prM. Moreover, our analyses showed that circulating DENV2 contain the highest number B cell epitopes targeted by neutralizing antibodies (see **Supplementary Table 4**). Of the 22 conserved epitopes in the three vaccine antigens, 13 of them are conserved in at least 50% of the circulating DENV2, while nine are conserved in the circulating DENV4 and DENV3 and seven are conserved in the circulating DENV1.

**Table 1:** B cell epitopes which are target for neutralizing antibodies and are conserved in the main dengue vaccines.

IEDB ID	Epitope Sequences	Vaccine in Which Epitope is Conserved	Location (protein)
173906	T331,N332,Q411,Y412,L415,K416,G439,T440,T445,P446,Q447,E452,I453,L455,G554,T555,L588,K590,E664,K665	LAV-TDV, DENGVAXIA®, TAK-003	E (DI, DII, and DIII.)
504078	W101,L107,G111	LAV-TDV, DENGVAXIA®, TAK-003	E (DII inside the fusion loop)
504083	R73,G78,E79	LAV-TDV, DENGVAXIA®, TAK-003	E (DII)
504136	F586,K587,L588,E589,K590,E591,V592,A593,E594,T595,G598,T599,V600,L601,V602,Q603,V604,K605,E642,I647,E648,S670,F672,K674,G675,S676,S677,I678,G679,K680	LAV-TDV, TAK-003	E (DIII and stem region)
240770	H438,S554,V589,K590,E591	LAV-TDV, DENGVAXIA®, TAK-003	E (DI and DII)
240773	T350,E351,S352,C354,Q357,S361,L362,N363,E364,R379,W381,G382,N383,G384,C385,G386,I393,T395,K526,K527,Q528	LAV-TDV, DENGVAXIA®, TAK-003	E (DII inside and outside the fusion loop)
504074	N103,G104,G111	LAV-TDV, DENGVAXIA®, TAK-003	E (DII inside the fusion loop)
504134	F586,K587,V588,V589,K590,E591,I592,A593,E594,T595,H597,T599,I600,V601,R603,Q605,V645,N646,I647,E648,S676,S677	LAV-TDV, DENGVAXIA®, TAK-003	E (DIII and stem region)
540687	K305,K307,K310	LAV-TDV, DENGVAXIA®, TAK-003	E (DIII)
540688	K305,K310,E311	LAV-TDV, DENGVAXIA®, TAK-003	E (DIII)
540689	T303,G304,K307	LAV-TDV, DENGVAXIA®, TAK-003	E (DIII)
753469	K307,V309,K310,Q316,G318,D362,S363,P364	LAV-TDV, DENGVAXIA®, TAK-003	E (DIII)
753470	K307,V309,Q316,D362,P364	LAV-TDV, DENGVAXIA®, TAK-003	E (DI and DII)
753471	K160,E161,I162,K163,I170,T171,A173,E174,T176,G177,T180	LAV-TDV, DENGVAXIA®, TAK-003	E (DII)
178102	W101,L107,G109	LAV-TDV, DENGVAXIA®, TAK-003	E (DII inside the fusion loop)
196271	L117,S119,E123,K140	LAV-TDV, DENGVAXIA®, TAK-003	PrM
241577	A50,T51,Q52,L53,A54,T55,R73,C74,W101,G106,E126,K128,V130,Q131,E133,N134,Q148,L196,T198,T274,I276,K307,K308,E309	LAV-TDV, DENGVAXIA®, TAK-003	E (DII inside and outside the fusion loop and DIII)
504117	V585,L586,K587,K588,E589,V590,S591,E592,G596,T597,I598,L599,I600,K601,V602,E603,V643,N644,I645,I667,W669,S674	LAV-TDV, DENGVAXIA®, TAK-003	E (DIII)
538524	Q52,L53,E126,K128,E133,L135,A203	LAV-TDV, DENGVAXIA®, TAK-003	E (DII and DIII)
504135	F585,S586,I587,D588,K589,E590,M591,A592,E593,T594,T599,V600,V601,K602,V603,K604,E606,N641,V643,T644,N645,I646,L668,H669,W670,G678,K679	LAV-TDV, DENGVAXIA®	E (DIII and stem region)
591353	K330, K403, K479, K481	LAV-TDV, DENGVAXIA®, TAK-003	E (DI and DII)
591354	K330, V332,K403, L414, K479, K513	LAV-TDV, DENGVAXIA®, TAK-003	E (DI and DII)

**Figure 2:**
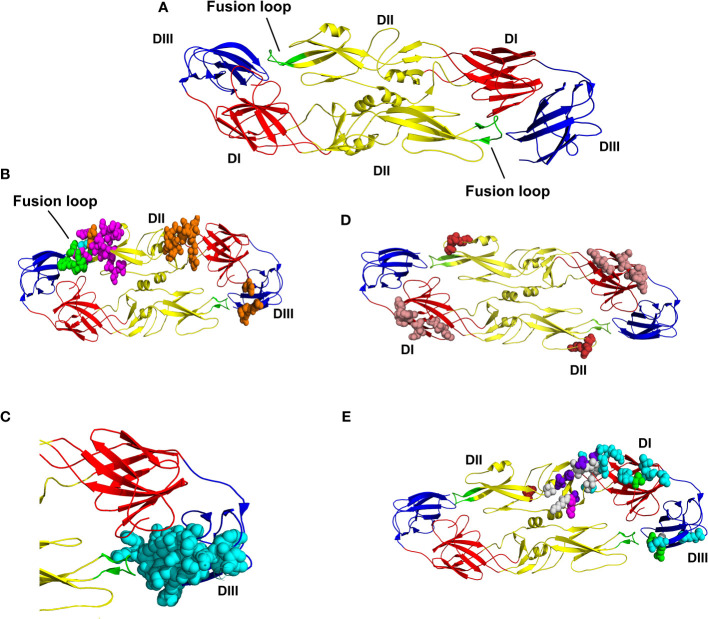
Location B cell epitopes that are the target for neutralizing antibodies and conserved in dengue vaccines on the 3D structure of envelope glycoprotein dimers. **(A)** 3D structure of envelope glycoprotein dimer. Domain I (DI) is shown in red, Domains II (DII) is shown in yellow, fusion loop is shown in green, and Domains III (DIII) is shown in blue. **(B)** Location of the 5 epitopes that share residues in the fusion loop, shown in green. Three of these epitopes contain all of their residues located in the fusion loop (504078, 504074 e 178102). The epitope ID 240773 (magenta) also has residues in DII and the epitope ID 241577 (orange) also has epitopes in DII and DIII. Residues shared by these two epitopes out of the fusion loop are shown in cyan. **(C)** Nine epitopes shown in cyan are located in DIII (IDs 540687, 540688, 540689, 753469, 753470, 504117, 504136, 504134, and 504135). The three last epitopes have residues located at the stem region; which was not represented in this 3D model. **(D)** Representation of the epitope with ID 753471, with residues shown in salmon. All residues are located at DII. **(E)** Residues shared by six epitopes are shown in white and are located in domains I, II, and III. The epitopes with residues located in the DI and DII are shown in brick (ID 591354), green (ID 240770), bluish-purple (ID 538524) and in magenta (ID591353). The epitope with ID 173906 is represented with residues in cyan, which are located in DI, DII, and DIII.

We identified the presence of a diverse distribution, with epitopes inserted in all regions of the envelope glycoprotein: DI, shown in red; DII, shown in yellow, DIII, shown in blue and the fusion loop shown in green (see **Figure 2A**). The residues W101, N103, G104, G106, L107, and G111, represented in **Figure 2B**, are shared with the five epitopes inserted in the fusion loop region. Epitopes with ID (504078, 504074, and 178102) are highlighted and contain all their residues fully inserted in the fusion loop region. The epitope with ID 240773 (magenta) also has residues in DII outside the fusion loop and the epitope with ID 241577 (orange) also has epitopes in DII outside the fusion loop and DIII. Residues shared by these two epitopes out of the fusion loop are shown in cyan. The Epitopes with ID 504136, 504134, and 504135 contain amino acids in the DIII, and also share common amino acids in the stem region, which is not present in the model used in this study (**Figure 2C**). Amino acids of the epitope with ID 753471 are all located in the DI, shown in salmon; amino acids of the epitope with ID 504083 (**Figure 2D**) are all located in the DII; and amino acids of epitopes with ID 540687, 540688, 540689, 753469, 753470 and 504117 are all located in the DIII, shown in cyan (**Figure 2C**). There are also epitopes with amino acids located in two or three domains: in DI and DII, epitopes with ID 591354 (shown in brick), ID 240770 (shown in green), ID 538524 (shown in luish-purple), and ID 591353 (shown in magenta) (**Figure 2E**); in DII (outside the loop) and DIII, epitopes with ID 240773 (shown in magenta) and 241577 (shown in orange) (**Figure 2B**); and in DI, DII and DIII, the epitope with ID 173906 (shown in cyan) (**Figure 2E**). Finally, only one epitope is located in the prM (ID 241577). It is conserved in the three vaccines, as shown in **Table 1**. It is important to highlight that some of the epitopes presented here are conserved in both vaccines and circulating viruses (see **Supplementary Table 4**). These results suggest that the three vaccines antigens analyzed in this study contain most of B cell epitopes targeted by neutralizing antibodies.

### Conservation Analysis of T Cell Epitopes Involved in Protective Immunity

The conservation analysis revealed that 29 out of 167 T cell epitopes were not conserved in the three vaccine antigens, whereas two of them were not preserved in either vaccine or circulating viruses (**Supplementary Table 5**).

**Figure 3:**
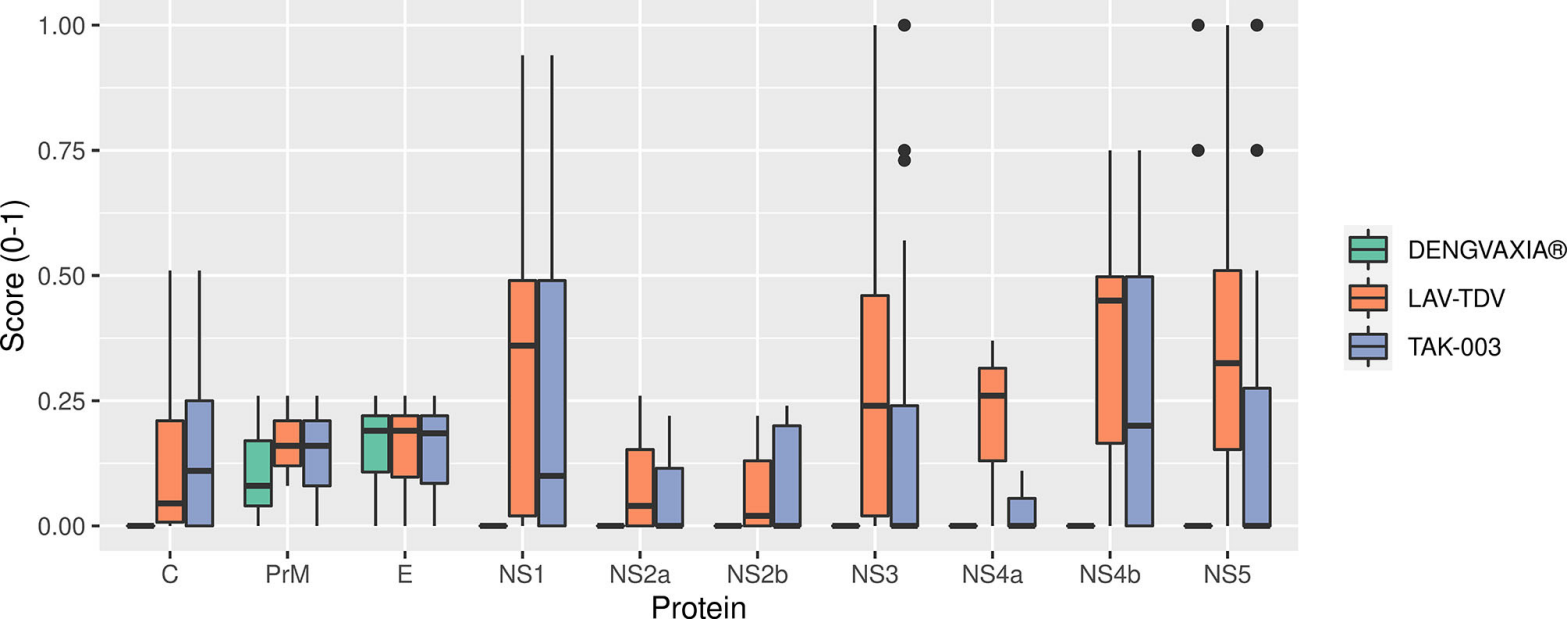
Distribution of protein scores in the three vaccines.

The DENGVAXIA^®^ vaccine presented only 23 conserved epitopes – a number relevantly smaller if compared to the number of epitopes presented by TAK-003 and LAV-TDV vaccines, 72 and 105, respectively. Most of the DENGVAXIA^®^ vaccine’s epitopes were located at the envelope glycoprotein. However, two and five of them were found in prM and NS5, respectively. In contrast, the LAV-TDV and TAK-003 vaccines preserved epitopes all over the 10 proteins (structural and non-structural). A score-based scale associated with conservation percentages of epitopes obtained from circulating viruses (**Supplementary Table 6**) was used to statistically compare conservations of epitopes in the three studied dengue vaccines. As shown in **Table 2** and **Figure 3**, significant numbers of epitopes in NS1, NS2a, NS3, NS4B, and NS5 are not present in the DENGVAXIA^®^ vaccine. Collectively, these results suggest that TAK-003 and LAV-TDV vaccines present higher conservation rates of epitopes involved in protective immunity than the DENGVAXIA^®^ vaccine, which lacks most of T cell epitopes involved in protective immunity against DENV.

**Table 2:** Results of the medians of the epitope set scores and the p-values derived from comparative analyzes between vaccines.

Protein	Median of Epitope Set Scores			P-value
	Dengvaxia®	LAV-TDV	TAK-003	
C	0.000	0.143	0.165	0.07758
prM	0.113	0.167	0.140	0.82796
E	0.168	0.161	0.149	0.81011
NS1	0.000	0.321	0.241	0.00716
NS2A	0.000	0.085	0.062	0.00585
NS2B	0.000	0.074	0.088	0.18181
NS3	0.000	0.284	0.147	0.00000
NS4A	0.000	0.210	0.037	0.19875
NS4B	0.000	0.361	0.280	0.00022
NS5	0.067	0.352	0.207	0.00002

### Analysis of Population Coverage of T Cell Epitopes Based on HLAs

**Figure 4:**
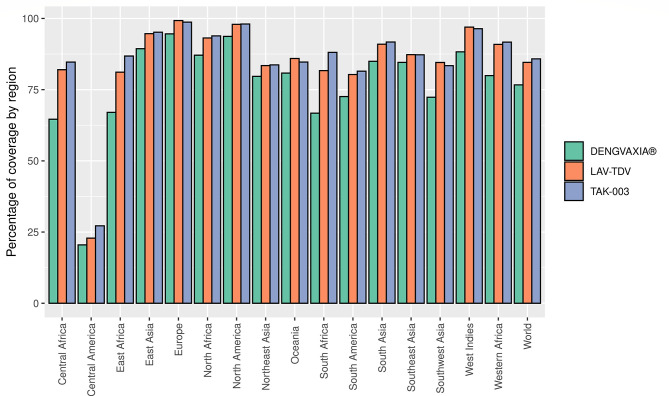
Percentage of coverage predicted for each vaccine based on the presence of a set of epitopes.

The 167 epitopes were evaluated regarding their coverage in the world population. The epitope sets for each vaccine were analyzed separately. Population coverage was shown to be high for all three vaccines worldwide, with percentages of 76.67% for DENGVAXIA^®^, 84.58% for LAV-TDV, and 85.80% for TAK-003. In addition, these vaccines had a population coverage of more than 70% in different continents and subcontinents in the world, except for Central America, which obtained a low percentage of 20.48%, 22.86%, and 27.18%, for DENGVAXIA^®^, LAV-TDV, and TAK-003, respectively. These results suggest that vaccine antigens studied here present high population coverage, mainly TAK-003 and LAV-TDV vaccine antigens (see **Figure 4**).

## Discussion

Dengue is a one of most common arboviral diseases. According to the World Health Organization, the development of safe and effective dengue vaccines is a priority. Therefore, in this study, we compared the main dengue vaccine antigens regarding their immunological properties. We studied DENGVAXIA^®^ from Sanofi Pasteur, which is a licensed vaccine; LAV-TDV from NIAID/Butantan, which is under phase III clinical trial and TAK-003 from Takeda, which finalized phase III clinical trial.

Neutralizing antibodies play a key role in controlling viral infections (24). Thus, much of the research to assess the immunological protection capacity of anti-dengue vaccines is based on the detection and/or evaluation of neutralizing antibodies (25–28). Therefore, we evaluated conservation of B cell epitopes for neutralizing antibodies in the three main dengue vaccines studied here. Most of the B cell epitopes here evaluated were located in the envelope glycoprotein, which is involved in the binding of DENV to host cell receptors. It is also involved in membrane fusion and is divided into three domains (DI, DII, and DIII). DII is central in each monomer and surrounded by DI and DIII (29). The hydrophobic fusion loop is located in DII and is highly conserved in DENV serotypes due to its important role in mediating membrane fusion (20, 29). Minimal changes in fusion loop amino acid sequences prevent membrane fusion (30). This explains the conservation of five epitopes for neutralizing antibodies in this region, despite the high selective pressure imposed by the host immune system on the whole envelope glycoprotein. These findings are in agreement with our previous report about the conservation of B cell epitopes for neutralizing antibodies in the *Flavivirus* genus (20). In addition, we found epitopes for neutralizing antibodies conserved in the DIII, which also corroborates our previous report considering the *Flavivirus* genus (20). The role of DIII in the DENV cycle is frequently related to a receptor binding function (29, 31). Antibodies targeting different regions in DIII were shown to strongly neutralize West Nile virus, a *Flavivirus* as DENV. Therefore, the concentration of epitopes for neutralizing antibodies in this domain was already expected. We also found epitopes with residues completely located at DI and DII or with part of residues located in these regions. These structures have important roles in changes in protein structure which occur before membrane fusion events. Finally, the single epitope located in the prM suggest that most epitopes which are target for neutralizing antibodies and are highly conserved have residues located in structures with key biological functions in the virus life cycle, such as the E protein.

The protective role of T lymphocytes during viral infections has been well established (32). Several findings have suggested that both, CD4+ and CD8+ T lymphocytes have a direct relationship with the establishment of the immune response since CD4+ T lymphocytes control viral infection through mechanisms such as: i) the increase in B lymphocyte responses and CD8+ lymphocytes, ii) production of inflammatory and antiviral cytokines, iii) cytotoxicity against infected cells, in addition to stimulating immune memory (33–37). It was previously reported that the repertoire of T cell epitopes for DENV is distributed throughout the virus proteome (38). We then computed conservation and population coverage of T cell epitopes involved in protective immunity against dengue, as previously described by us (20). Most of these epitopes are concentrated in NS3, NS5 and NS4B proteins (13, 14, 20, 38). Our results suggest that in Dengvaxia^®^, a chimeric vaccine in which non-structural proteins are those from *Yellow fever virus*, most of the important T cell epitopes involved in protective immunity are not present. This is probably the explanation for the low protective efficacy of Dengvaxia^®^ when compared to TAK-003. For example: clinical trials carried out with Dengvaxia^®^ revealed an efficacy of 60.3% (95%CI. 55.7 to 64.5) in children and adolescents from 2 to 16 years old (39) and an efficacy of 30.2% (95%CI. 13.4 to 56.6) in those aged between 4 and 11 years old (40). In contrast, TAK-003 showed an efficacy of 80.9% (95%CI. 75.2 to 85.3) in children and adolescents from 4 to 16 years old (41). In addition, it was found that hospitalization rates due to severe dengue were significantly higher in dengue naïve children who received Dengvaxia^®^ (42). Although this vaccine formulation had achieved equivalent percentages of population coverage regarding LAV-TDV and TAK-003, our results suggest that investing in vaccines that contain the majority of epitopes involved in protective immunity (cellular and humoral arms) is an important issue to be considered.

## Data Availability Statement

The original contributions presented in the study are included in the article/**Supplementary Material**. Further inquiries can be directed to the corresponding author.

## Author Contributions

JP collected data, analyzed data, interpreted data, prepared figures and wrote the manuscript. EC, RS, AR, LS, VS, ST and BR collected data, analyzed data and wrote the first version of manuscript. AB, EM and WL interpreted the data generated, prepared figures and wrote the manuscript. JA conceived the study, analyzed data, interpreted the data generated and wrote the manuscript. All authors contributed to the article and approved the submitted version.

## Funding

Instituto Serrapilheira/Serra-1708-15285, Consórcio Multifinalitário do Oeste da Bahia (CONSID-001), 27968 FINEP/RTR/PRPq/REDE COVID-19 (9-UFOB). AB is supported by a grant from Instituto Serrapilheira/Serra-1708-15285. JA is supported by grants from Consórcio Multifinalitário do Oeste da Bahia and 27968 FINEP/RTR/PRPq/ REDE COVID-19. The funders had no role in study design, data collection and analysis, decision to publish, or preparation of the manuscript.

## Conflict of Interest

The authors declare that the research was conducted in the absence of any commercial or financial relationships that could be construed as a potential conflict of interest.

## Publisher’s Note

All claims expressed in this article are solely those of the authors and do not necessarily represent those of their affiliated organizations, or those of the publisher, the editors and the reviewers. Any product that may be evaluated in this article, or claim that may be made by its manufacturer, is not guaranteed or endorsed by the publisher.
