# Supplementary material for: Comparison of Neutralizing Dengue Virus B Cell Epitopes and Protective T Cell Epitopes With Those in Three Main Dengue Virus Vaccines
Source: Front Immunol. 2021 Aug 20;12:715136. doi: 10.3389/fimmu.2021.715136 (PMC8417696; doi:10.3389/fimmu.2021.715136)
Supplement: Supplementary file 1 [file DataSheet_1.pdf]

Supplementary data

>Dengue1_Dengvaxia
MSGRKAQGKTLGVNMVRRGVRSLSNKIKQKTKQIGNRPGPSRGVQGFIFFFLFNILTGKKITAHLKRLWKMLDPRQGLAVLRK
VKRVVASLMRGLSSRKRRSHDVLTVQFLILGMLLMTGGFHLTTRGGEPHMIVTKQERGKSLLFKTSAGVNMCTLIAMDLGELC
EDTMTYKCPRITEAEPDDVDCWCNATDTWVTYGTCSQTGEHRRDKRSVALAPHVGLGLETRTETWMSSEGAWKQIQRVETWAL
RHPGFTVIALFLAHAIGTSITQKGIIFILLMLVTPSMAMRCVGIGNRDFVEGLSGATWVDVVLEHGSCVTTMAKDKPTLDIEL
LKTEVTNPAVLRKLCIEAKISNTTTDSRCPTQGEATLVEEQDANFVCRRTFVDRGWGNGCGLFGKGSLITCAKFKCVTKLEGK
IVQYENLKYSVIVTVHTGDQHQVGNESTEHGTTATITPQAPTSEIQLTDYGALTLDCSPRTGLDFNEMVLLTMKERSWLVHKQ
WFLDLPLPWTSGATTSQETWNRQDLLVTFKTAHAKKQEVVVLGSQEGAMHTALTGATEIQTSGTTTIFAGHLKCRLKMDKLTL
KGMSYVMCTGSFKLEKEVAETQHGTVLVQVKYEGTDAPCKIPFSTQDEKGVTQNGRVITANPIVTDKEKPVNIEAEPPFGESY
IVVGAGEKALKLSWFKKGSTIGKMFEATARGARRMAILGDTAWDFGSIGGVFTSVGKLVHQIFGTAYGVLFSGVSWTMKIGIG
VLLTWLGLNSRSTSLSMTCIAVGLVTLYLGVMVGADQGCAINFGKRELKCGDGIFIFRDSDDWLNKYSYYPEDPVKLASIVKA
SFEEGKCGLNSVDSLEHEMWRSRADEINAIFEENEVDISVVVQDPKNVYQRGTHPFSRIRDGLQYGWKTWGKNLVFSPGRKNG
SFIIDGKSRKECPFSNRVWNSFQIEEFGTGVFTTRVYMDAVFEYTIDCDGSILGAAVNGKKSAHGSPTFWMGSHEVNGTWMIH
TLEALDYKECEWPLTHTIGTSVEESEMFMPRSIGGPVSSHNHIPGYKVQTNGPWMQVPLEVKREACPGTSVIIDGNCDGRGKS
TRSTTDSGKVIPEWCCRSCTMPPVSFHGSDGCWYPMEIRPRKTHESHLVRSWVTAGEIHAVPFGLVSMMIAMEVVLRKRQGPK
QMLVGGVVLLGAMLVGQVTLLDLLKLTVAVGLHFHEMNNGGDAMYMALIAAFSIRPGLLIGFGLRTLWSPRERLVLTLGAAMV
EIALGGVMGGLWKYLNAVSLCILTINAVASRKASNTILPLMALLTPVTMAEVRLAAMFFCAVVIIGVLHQNFKDTSMQKTIPL
VALTLTSYLGLTQPFLGLCAFLATRIFGRRSIPVNEALAAAGLVGVLAGLAFQEMENFLGPIAVGGLLMMLVSVAGRVDGLEL
KKLGEVSWEEEAEISGSSARYDVALSEQGEFKLLSEEKVPWDQVVMTSLALVGAALHPFALLLVLAGWLFHVRGARRSGDVLW
DIPTPKIIEECEHLEDGIYGIFQSTFLGASQRGVGVAQGGVFHTMWHVTRGAFLVRNGKKLIPSWASVKEDLVAYGGSWKLEG
RWDGEEEVQLIAAVPGKNVVNVQTKPSLFKVRNGGEIGAVALDYPSGTSGSPIVNRNGEVIGLYGNGILVGDNSFVSAISQTE
VKEEGKEELQEIPTMLKKGMTTVLDFHPGAGKTRRFLPQILAECARRRLRTLVLAPTRVVLSEMKEAFHGLDVKFHTQAFSAH
GSGREVIDAMCHATLTYRMLEPTRVVNWEVIIMDEAHFLDPASIAARGWAAHRARANESATILMTATPPGTSDEFPHSNGEIE
DVQTDIPSEPWNTGHDWILADKRPTAWFLPSIRAANVMAASLRKAGKSVVVLNRKTFEREYPTIKQKKPDFILATDIAEMGAN
LCVERVLDCRTAFKPVLVDEGRKVAIKGPLRISASSAAQRRGRIGRNPNRDGDSYYYSEPTSENNAHHVCWLEASMLLDNMEV
RGGMVAPLYGVEGTKTPVSPGEMRLRDDQRKVFRELVRNCDLPVWLSWQVAKAGLKTNDRKWCFEGPEEHEILNDSGETVKCR
APGGAKKPLRPRWCDERVSSDQSALSEFIKFAEGRRGAAEVLVVLSELPDFLAKKGGEAMDTISVFLHSEEGSRAYRNALSMM
PEAMTIVMLFILAGLLTSGMVIFFMSPKGISRMSMAMGTMAGCGYLMFLGGVKPTHISYVMLIFFVLMVVVIPEPGQQRSIQD
NQVAYLIIGILTLVSAVAANELGMLEKTKEDLFGKKNLIPSSASPWSWPDLDLKPGAAWTVYVGIVTMLSPMLHHWIKVEYGN
LSLSGIAQSASVLSFMDKGIPFMKMNISVIMLLVSGWNSITVMPLLCGIGCAMLHWSLILPGIKAQQSKLAQRRVFHGVAENP
VVDGNPTVDIEEAPEMPALYEKKLALYLLLALSLASVAMCRTPFSLAEGIVLASAALGPLIEGNTSLLWNGPMAVSMTGVMRG
NHYAFVGVMYNLWKMKTGRRGSANGKTLGEVWKRELNLLDKRQFELYKRTDIVEVDRDTARRHLAEGKVDTGVAVSRGTAKLR
WFHERGYVKLEGRVIDLGCGRGGWCYYAAAQKEVSGVKGFTLGRDGHEKPMNVQSLGWNIITFKDKTDIHRLEPVKCDTLLCD
IGESSSSSVTEGERTVRVLDTVEKWLACGVDNFCVKVLAPYMPDVLEKLELLQRRFGGTVIRNPLSRNSTHEMYYVSGARSNV
TFTVNQTSRLLMRRMRRPTGKVTLEADVILPIGTRSVETDKGPLDKEAIEERVERIKSEYMTSWFYDNDNPYRTWHYCGSYVT
KTSGSAASMVNGVIKILTYPWDRIEEVTRMAMTDTTPFGQQRVFKEKVDTRAKDPPAGTRKIMKVVNRWLFRHLAREKNPRLC
TKEEFIAKVRSHAAIGAYLEEQEQWKTANEAVQDPKFWELVDEERKLHQQGRCRTCVYNMMGKREKKLSEFGKAKGSRAIWYM
WLGARYLEFEALGFLNEDHWASRENSGGGVEGIGLQYLGYVIRDLAAMDGGGFYADDTAGWDTRITEADLDDEQEILNYMSPH
HKKLAQAVMEMTYKNKVVKVLRPAPGGKAYMDVISRRDQRGSGQVVTYALNTITNLKVQLIRMAEAEMVIHHQHVQDCDESVL
TRLEAWLTEHGCDRLKRMAVSGDDCVVRPIDDRFGLALSHLNAMSKVRKDISEWQPSKGWNDWENVPFCSHHFHELQLKDGRR
IVVPCREQDELIGRGRVSPGNGWMIKETACLSKAYANMWSLMYFHKRDMRLLSLAVSSAVPTSWVPQGRTTWSIHGKGEWMTT
EDMLEVWNRVWITNNPHMQDKTMVKKWRDVPYLTKRQDKLCGSLIGMTNRATWASHIHLVIHRIRTLIGQEKYTDYLTVMDRY
SVDADLQLGELI

>Dengue2_Dengvaxia
MSGRKAQGKTLGVNMVRRGVRSLSNKIKQKTKQIGNRPGPSRGVQGFIFFFLFNILTGKKITAHLKRLWKMLDPRQGLAVLRK
VKRVVASLMRGLSSRKRRSHDVLTVQFLILGMLLMTGGFHLTTRNGEPHMIVSRQEKGKSLVFKTEDGVNMCTLMAMDLGELC
EDTITYKCPLLRQNEPEDIDCWCNSTSTWVTYGTCTTTGEHRREKRSVALVPHVGMGLETRTETWMSSEGAWKHAQRIEIWIL
RHPGFTIMAAILAYTIGTTHFQRALIFILLTAVAPSMTMRCIGISNRDFVEGVSGGSWVDIVLEHGSCVTTMAKNKPTLDFEL

IKTEAKQPATLRKYCIEAKLTNTTTESRCPTQGEPSLNEEQDKRFVCKHSMVDRGWGNGCGLFGKGGIVTCAMFTCKKNMEGK
VVQPENLEYTIVVTPHSGEEHAVGNDTGKHGKEIKVTPQSSITEAELTGYGTVTMECSPRTGLDFNEMVLLQMENKAWLVHRQ
WFLDLPLPWLPGADTQGSNWIQKETLVTFKNPHAKKQDVFVLGSQEGAMHTALTGATEIQMSSGNLLFTGHLKCRLRMDKLQL
KGMSYSMCTGKFKVVKEIAETQHGTIVIRVQYEGDGSPCKIPFEIMDLEKRHVLGRLITVNPIVTEKDSPVNIEAEPPFGDSY
IIIGVEPGQLKLNWFKKGSSIGQMFETTMRGAKRMAILGDTAWDFGSLGGVFTSIGKALHQVFGAIYGAAFSGVSWTMKILIG
VIITWIGMNSRSTSLSVSLVLVGVVTLYLGVMVGADQGCAINFGKRELKCGDGIFIFRDSDDWLNKYSYYPEDPVKLASIVKA
SFEEGKCGLNSVDSLEHEMWRSRADEINAIFEENEVDISVVVQDPKNVYQRGTHPFSRIRDGLQYGWKTWGKNLVFSPGRKNG
SFIIDGKSRKECPFSNRVWNSFQIEEFGTGVFTTRVYMDAVFEYTIDCDGSILGAAVNGKKSAHGSPTFWMGSHEVNGTWMIH
TLEALDYKECEWPLTHTIGTSVEESEMFMPRSIGGPVSSHNHIPGYKVQTNGPWMQVPLEVKREACPGTSVIIDGNCDGRGKS
TRSTTDSGKVIPEWCCRSCTMPPVSFHGSDGCWYPMEIRPRKTHESHLVRSWVTAGEIHAVPFGLVSMMIAMEVVLRKRQGPK
QMLVGGVVLLGAMLVGQVTLLDLLKLTVAVGLHFHEMNNGGDAMYMALIAAFSIRPGLLIGFGLRTLWSPRERLVLTLGAAMV
EIALGGVMGGLWKYLNAVSLCILTINAVASRKASNTILPLMALLTPVTMAEVRLAAMFFCAVVIIGVLHQNFKDTSMQKTIPL
VALTLTSYLGLTQPFLGLCAFLATRIFGRRSIPVNEALAAAGLVGVLAGLAFQEMENFLGPIAVGGLLMMLVSVAGRVDGLEL
KKLGEVSWEEEAEISGSSARYDVALSEQGEFKLLSEEKVPWDQVVMTSLALVGAALHPFALLLVLAGWLFHVRGARRSGDVLW
DIPTPKIIEECEHLEDGIYGIFQSTFLGASQRGVGVAQGGVFHTMWHVTRGAFLVRNGKKLIPSWASVKEDLVAYGGSWKLEG
RWDGEEEVQLIAAVPGKNVVNVQTKPSLFKVRNGGEIGAVALDYPSGTSGSPIVNRNGEVIGLYGNGILVGDNSFVSAISQTE
VKEEGKEELQEIPTMLKKGMTTVLDFHPGAGKTRRFLPQILAECARRRLRTLVLAPTRVVLSEMKEAFHGLDVKFHTQAFSAH
GSGREVIDAMCHATLTYRMLEPTRVVNWEVIIMDEAHFLDPASIAARGWAAHRARANESATILMTATPPGTSDEFPHSNGEIE
DVQTDIPSEPWNTGHDWILADKRPTAWFLPSIRAANVMAASLRKAGKSVVVLNRKTFEREYPTIKQKKPDFILATDIAEMGAN
LCVERVLDCRTAFKPVLVDEGRKVAIKGPLRISASSAAQRRGRIGRNPNRDGDSYYYSEPTSENNAHHVCWLEASMLLDNMEV
RGGMVAPLYGVEGTKTPVSPGEMRLRDDQRKVFRELVRNCDLPVWLSWQVAKAGLKTNDRKWCFEGPEEHEILNDSGETVKCR
APGGAKKPLRPRWCDERVSSDQSALSEFIKFAEGRRGAAEVLVVLSELPDFLAKKGGEAMDTISVFLHSEEGSRAYRNALSMM
PEAMTIVMLFILAGLLTSGMVIFFMSPKGISRMSMAMGTMAGCGYLMFLGGVKPTHISYVMLIFFVLMVVVIPEPGQQRSIQD
NQVAYLIIGILTLVSAVAANELGMLEKTKEDLFGKKNLIPSSASPWSWPDLDLKPGAAWTVYVGIVTMLSPMLHHWIKVEYGN
LSLSGIAQSASVLSFMDKGIPFMKMNISVIMLLVSGWNSITVMPLLCGIGCAMLHWSLILPGIKAQQSKLAQRRVFHGVAENP
VVDGNPTVDIEEAPEMPALYEKKLALYLLLALSLASVAMCRTPFSLAEGIVLASAALGPLIEGNTSLLWNGPMAVSMTGVMRG
NHYAFVGVMYNLWKMKTGRRGSANGKTLGEVWKRELNLLDKRQFELYKRTDIVEVDRDTARRHLAEGKVDTGVAVSRGTAKLR
WFHERGYVKLEGRVIDLGCGRGGWCYYAAAQKEVSGVKGFTLGRDGHEKPMNVQSLGWNIITFKDKTDIHRLEPVKCDTLLCD
IGESSSSSVTEGERTVRVLDTVEKWLACGVDNFCVKVLAPYMPDVLEKLELLQRRFGGTVIRNPLSRNSTHEMYYVSGARSNV
TFTVNQTSRLLMRRMRRPTGKVTLEADVILPIGTRSVETDKGPLDKEAIEERVERIKSEYMTSWFYDNDNPYRTWHYCGSYVT
KTSGSAASMVNGVIKILTYPWDRIEEVTRMAMTDTTPFGQQRVFKEKVDTRAKDPPAGTRKIMKVVNRWLFRHLAREKNPRLC
TKEEFIAKVRSHAAIGAYLEEQEQWKTANEAVQDPKFWELVDEERKLHQQGRCRTCVYNMMGKREKKLSEFGKAKGSRAIWYM
WLGARYLEFEALGFLNEDHWASRENSGGGVEGIGLQYLGYVIRDLAAMDGGGFYADDTAGWDTRITEADLDDEQEILNYMSPH
HKKLAQAVMEMTYKNKVVKVLRPAPGGKAYMDVISRRDQRGSGQVVTYALNTITNLKVQLIRMAEAEMVIHHQHVQDCDESVL
TRLEAWLTEHGCDRLKRMAVSGDDCVVRPIDDRFGLALSHLNAMSKVRKDISEWQPSKGWNDWENVPFCSHHFHELQLKDGRR
IVVPCREQDELIGRGRVSPGNGWMIKETACLSKAYANMWSLMYFHKRDMRLLSLAVSSAVPTSWVPQGRTTWSIHGKGEWMTT
EDMLEVWNRVWITNNPHMQDKTMVKKWRDVPYLTKRQDKLCGSLIGMTNRATWASHIHLVIHRIRTLIGQEKYTDYLTVMDRY
SVDADLQLGELI
>Dengue3_Dengvaxia
MSGRKAQGKTLGVNMVRRGVRSLSNKIKQKTKQIGNRPGPSRGVQGFIFFFLFNILTGKKITAHLKRLWKMLDPRQGLAVLRK
VKRVVASLMRGLSSRKRRSHDVLTVQFLILGMLLMTGGFHLTSRDGEPRMIVGKNERGKSLLFKTASGINMCTLIAMDLGEMC
DDTVTYKCPLIAEVEPEDIDCWCNLTSTWVTYGTCNQAGEHRRDKRSVALAPHVGMGLDTRTQTWMSAEGAWRQVEKVETWAL
RHPGFTILALFLAHYIGTSLTQKVVIFILLMLVTPSMAMRCVGVGNRDFVEGLSGATWVDVVLEHGGCVTTMAKNKPTLDIEL
QKTEATQLATLRKLCIEGKITNITTDSRCPTQGEAILPEEQDQNYVCKHTYVDRGWGNGCGLFGKGSLVTCAKFQCLESIEGK
VVQHENLKYTVIITVHTGNQHQVGNDTQGVTAEITPQASTVEAILPEYGTLGLECSPRTGLDFNEMILLTMKNKAWMVHRQWF
FDLPLPWTSGATTETPTWNRKELLVTFKNAHAKKQEVVVLGSQEGAMHTALTGATEIQNSGGTSIFAGHLKCRLKMDKLELKG
MSYAMCLNTFVLKKEVSETQHGTILIKVEYKGEDAPCKIPFSTEDGQGKAHNGRLITANPVVTKKEEPVNIEAEPPFGESNIV
IGIGDKALKINWYKKGSSIGKMFEATARGARRMAILGDTAWDFGSVGGVLNSLGKMVHQIFGSAYTALFSGVSWIMKIGIGVL

LTWIGLNSKNTSMSFSCVAIGIITLYLGAVVQADQGCAINFGKRELKCGDGIFIFRDSDDWLNKYSYYPEDPVKLASIVKASF
EEGKCGLNSVDSLEHEMWRSRADEINAIFEENEVDISVVVQDPKNVYQRGTHPFSRIRDGLQYGWKTWGKNLVFSPGRKNGSF
IIDGKSRKECPFSNRVWNSFQIEEFGTGVFTTRVYMDAVFEYTIDCDGSILGAAVNGKKSAHGSPTFWMGSHEVNGTWMIHTL
EALDYKECEWPLTHTIGTSVEESEMFMPRSIGGPVSSHNHIPGYKVQTNGPWMQVPLEVKREACPGTSVIIDGNCDGRGKSTR
STTDSGKVIPEWCCRSCTMPPVSFHGSDGCWYPMEIRPRKTHESHLVRSWVTAGEIHAVPFGLVSMMIAMEVVLRKRQGPKQM
LVGGVVLLGAMLVGQVTLLDLLKLTVAVGLHFHEMNNGGDAMYMALIAAFSIRPGLLIGFGLRTLWSPRERLVLTLGAAMVEI
ALGGVMGGLWKYLNAVSLCILTINAVASRKASNTILPLMALLTPVTMAEVRLAAMFFCAVVIIGVLHQNFKDTSMQKTIPLVA
LTLTSYLGLTQPFLGLCAFLATRIFGRRSIPVNEALAAAGLVGVLAGLAFQEMENFLGPIAVGGLLMMLVSVAGRVDGLELKK
LGEVSWEEEAEISGSSARYDVALSEQGEFKLLSEEKVPWDQVVMTSLALVGAALHPFALLLVLAGWLFHVRGARRSGDVLWDI
PTPKIIEECEHLEDGIYGIFQSTFLGASQRGVGVAQGGVFHTMWHVTRGAFLVRNGKKLIPSWASVKEDLVAYGGSWKLEGRW
DGEEEVQLIAAVPGKNVVNVQTKPSLFKVRNGGEIGAVALDYPSGTSGSPIVNRNGEVIGLYGNGILVGDNSFVSAISQTEVK
EEGKEELQEIPTMLKKGMTTVLDFHPGAGKTRRFLPQILAECARRRLRTLVLAPTRVVLSEMKEAFHGLDVKFHTQAFSAHGS
GREVIDAMCHATLTYRMLEPTRVVNWEVIIMDEAHFLDPASIAARGWAAHRARANESATILMTATPPGTSDEFPHSNGEIEDV
QTDIPSEPWNTGHDWILADKRPTAWFLPSIRAANVMAASLRKAGKSVVVLNRKTFEREYPTIKQKKPDFILATDIAEMGANLC
VERVLDCRTAFKPVLVDEGRKVAIKGPLRISASSAAQRRGRIGRNPNRDGDSYYYSEPTSENNAHHVCWLEASMLLDNMEVRG
GMVAPLYGVEGTKTPVSPGEMRLRDDQRKVFRELVRNCDLPVWLSWQVAKAGLKTNDRKWCFEGPEEHEILNDSGETVKCRAP
GGAKKPLRPRWCDERVSSDQSALSEFIKFAEGRRGAAEVLVVLSELPDFLAKKGGEAMDTISVFLHSEEGSRAYRNALSMMPE
AMTIVMLFILAGLLTSGMVIFFMSPKGISRMSMAMGTMAGCGYLMFLGGVKPTHISYVMLIFFVLMVVVIPEPGQQRSIQDNQ
VAYLIIGILTLVSAVAANELGMLEKTKEDLFGKKNLIPSSASPWSWPDLDLKPGAAWTVYVGIVTMLSPMLHHWIKVEYGNLS
LSGIAQSASVLSFMDKGIPFMKMNISVIMLLVSGWNSITVMPLLCGIGCAMLHWSLILPGIKAQQSKLAQRRVFHGVAENPVV
DGNPTVDIEEAPEMPALYEKKLALYLLLALSLASVAMCRTPFSLAEGIVLASAALGPLIEGNTSLLWNGPMAVSMTGVMRGNH
YAFVGVMYNLWKMKTGRRGSANGKTLGEVWKRELNLLDKRQFELYKRTDIVEVDRDTARRHLAEGKVDTGVAVSRGTAKLRWF
HERGYVKLEGRVIDLGCGRGGWCYYAAAQKEVSGVKGFTLGRDGHEKPMNVQSLGWNIITFKDKTDIHRLEPVKCDTLLCDIG
ESSSSSVTEGERTVRVLDTVEKWLACGVDNFCVKVLAPYMPDVLEKLELLQRRFGGTVIRNPLSRNSTHEMYYVSGARSNVTF
TVNQTSRLLMRRMRRPTGKVTLEADVILPIGTRSVETDKGPLDKEAIEERVERIKSEYMTSWFYDNDNPYRTWHYCGSYVTKT
SGSAASMVNGVIKILTYPWDRIEEVTRMAMTDTTPFGQQRVFKEKVDTRAKDPPAGTRKIMKVVNRWLFRHLAREKNPRLCTK
EEFIAKVRSHAAIGAYLEEQEQWKTANEAVQDPKFWELVDEERKLHQQGRCRTCVYNMMGKREKKLSEFGKAKGSRAIWYMWL
GARYLEFEALGFLNEDHWASRENSGGGVEGIGLQYLGYVIRDLAAMDGGGFYADDTAGWDTRITEADLDDEQEILNYMSPHHK
KLAQAVMEMTYKNKVVKVLRPAPGGKAYMDVISRRDQRGSGQVVTYALNTITNLKVQLIRMAEAEMVIHHQHVQDCDESVLTR
LEAWLTEHGCDRLKRMAVSGDDCVVRPIDDRFGLALSHLNAMSKVRKDISEWQPSKGWNDWENVPFCSHHFHELQLKDGRRIV
VPCREQDELIGRGRVSPGNGWMIKETACLSKAYANMWSLMYFHKRDMRLLSLAVSSAVPTSWVPQGRTTWSIHGKGEWMTTED
MLEVWNRVWITNNPHMQDKTMVKKWRDVPYLTKRQDKLCGSLIGMTNRATWASHIHLVIHRIRTLIGQEKYTDYLTVMDRYSV
DADLQLGELI
>Dengue4_Dengvaxia
MSGRKAQGKTLGVNMVRRGVRSLSNKIKQKTKQIGNRPGPSRGVQGFIFFFLFNILTGKKITAHLKRLWKMLDPRQGLAVLRK
VKRVVASLMRGLSSRKRRSHDVLTVQFLILGMLLMTGGFHLSTRDGEPLMIVAKHERGRPLLFKTTEGINKCTLIAMDLGEMC
EDTVTYKCPLLVNTEPEDIDCWCNLTSTWVMYGTCTQSGERRREKRSVALTPHSGMGLETRAETWMSSEGAWKHAQRVESWIL
RNPGFALLAGFMAYMIGQTGIQRTVFFVLMMLVAPSYGMRCVGVGNRDFVEGVSGGAWVDLVLEHGGCVTTMAQGKPTLDFEL
TKTTAKEVALLRTYCIEASISNITTATRCPTQGEPYLKEEQDQQYICRRDVVDRGWGNGCGLFGKGGVVTCAKFLCSGKITGN
LVQIENLEYTVVVTVHNGDTHAVGNDTSNHGVTATITPRSPSVEVKLPDYGELTLDCEPRSGIDFNEMILMKMKKKTWLVHKQ
WFLDLPLPWTAGADTSEVHWNYKERMVTFKVPHAKRQDVTVLGSQEGAMHSALAGATEVDSGDGNHMFAGHLKCKVRMEKLRI
KGMSYTMCSGKFSIDKEMAETQHGTTVVKVKYEGAGAPCKVPIEIRDVNKEKVVGRIISSTPFAENTNSVTNIELEPPFGDSY
IVIGVGDSALTLHWFRKGSSIGKMFESTYRGAKRMAILGETAWDFGSVGGMFTSLGKAVHQVFGSVYTTMFGGVSWMVRILIG
FLVLWIGTNSRNTSMAMTCIAVGGITLFLGFTVGADQGCAINFGKRELKCGDGIFIFRDSDDWLNKYSYYPEDPVKLASIVKA
SFEEGKCGLNSVDSLEHEMWRSRADEINAIFEENEVDISVVVQDPKNVYQRGTHPFSRIRDGLQYGWKTWGKNLVFSPGRKNG
SFIIDGKSRKECPFSNRVWNSFQIEEFGTGVFTTRVYMDAVFEYTIDCDGSILGAAVNGKKSAHGSPTFWMGSHEVNGTWMIH
TLEALDYKECEWPLTHTIGTSVEESEMFMPRSIGGPVSSHNHIPGYKVQTNGPWMQVPLEVKREACPGTSVIIDGNCDGRGKS
TRSTTDSGKVIPEWCCRSCTMPPVSFHGSDGCWYPMEIRPRKTHESHLVRSWVTAGEIHAVPFGLVSMMIAMEVVLRKRQGPK

QMLVGGVVLLGAMLVGQVTLLDLLKLTVAVGLHFHEMNNGGDAMYMALIAAFSIRPGLLIGFGLRTLWSPRERLVLTLGAAMV
EIALGGVMGGLWKYLNAVSLCILTINAVASRKASNTILPLMALLTPVTMAEVRLAAMFFCAVVIIGVLHQNFKDTSMQKTIPL
VALTLTSYLGLTQPFLGLCAFLATRIFGRRSIPVNEALAAAGLVGVLAGLAFQEMENFLGPIAVGGLLMMLVSVAGRVDGLEL
KKLGEVSWEEEAEISGSSARYDVALSEQGEFKLLSEEKVPWDQVVMTSLALVGAALHPFALLLVLAGWLFHVRGARRSGDVLW
DIPTPKIIEECEHLEDGIYGIFQSTFLGASQRGVGVAQGGVFHTMWHVTRGAFLVRNGKKLIPSWASVKEDLVAYGGSWKLEG
RWDGEEEVQLIAAVPGKNVVNVQTKPSLFKVRNGGEIGAVALDYPSGTSGSPIVNRNGEVIGLYGNGILVGDNSFVSAISQTE
VKEEGKEELQEIPTMLKKGMTTVLDFHPGAGKTRRFLPQILAECARRRLRTLVLAPTRVVLSEMKEAFHGLDVKFHTQAFSAH
GSGREVIDAMCHATLTYRMLEPTRVVNWEVIIMDEAHFLDPASIAARGWAAHRARANESATILMTATPPGTSDEFPHSNGEIE
DVQTDIPSEPWNTGHDWILADKRPTAWFLPSIRAANVMAASLRKAGKSVVVLNRKTFEREYPTIKQKKPDFILATDIAEMGAN
LCVERVLDCRTAFKPVLVDEGRKVAIKGPLRISASSAAQRRGRIGRNPNRDGDSYYYSEPTSENNAHHVCWLEASMLLDNMEV
RGGMVAPLYGVEGTKTPVSPGEMRLRDDQRKVFRELVRNCDLPVWLSWQVAKAGLKTNDRKWCFEGPEEHEILNDSGETVKCR
APGGAKKPLRPRWCDERVSSDQSALSEFIKFAEGRRGAAEVLVVLSELPDFLAKKGGEAMDTISVFLHSEEGSRAYRNALSMM
PEAMTIVMLFILAGLLTSGMVIFFMSPKGISRMSMAMGTMAGCGYLMFLGGVKPTHISYVMLIFFVLMVVVIPEPGQQRSIQD
NQVAYLIIGILTLVSAVAANELGMLEKTKEDLFGKKNLIPSSASPWSWPDLDLKPGAAWTVYVGIVTMLSPMLHHWIKVEYGN
LSLSGIAQSASVLSFMDKGIPFMKMNISVIMLLVSGWNSITVMPLLCGIGCAMLHWSLILPGIKAQQSKLAQRRVFHGVAENP
VVDGNPTVDIEEAPEMPALYEKKLALYLLLALSLASVAMCRTPFSLAEGIVLASAALGPLIEGNTSLLWNGPMAVSMTGVMRG
NHYAFVGVMYNLWKMKTGRRGSANGKTLGEVWKRELNLLDKRQFELYKRTDIVEVDRDTARRHLAEGKVDTGVAVSRGTAKLR
WFHERGYVKLEGRVIDLGCGRGGWCYYAAAQKEVSGVKGFTLGRDGHEKPMNVQSLGWNIITFKDKTDIHRLEPVKCDTLLCD
IGESSSSSVTEGERTVRVLDTVEKWLACGVDNFCVKVLAPYMPDVLEKLELLQRRFGGTVIRNPLSRNSTHEMYYVSGARSNV
TFTVNQTSRLLMRRMRRPTGKVTLEADVILPIGTRSVETDKGPLDKEAIEERVERIKSEYMTSWFYDNDNPYRTWHYCGSYVT
KTSGSAASMVNGVIKILTYPWDRIEEVTRMAMTDTTPFGQQRVFKEKVDTRAKDPPAGTRKIMKVVNRWLFRHLAREKNPRLC
TKEEFIAKVRSHAAIGAYLEEQEQWKTANEAVQDPKFWELVDEERKLHQQGRCRTCVYNMMGKREKKLSEFGKAKGSRAIWYM
WLGARYLEFEALGFLNEDHWASRENSGGGVEGIGLQYLGYVIRDLAAMDGGGFYADDTAGWDTRITEADLDDEQEILNYMSPH
HKKLAQAVMEMTYKNKVVKVLRPAPGGKAYMDVISRRDQRGSGQVVTYALNTITNLKVQLIRMAEAEMVIHHQHVQDCDESVL
TRLEAWLTEHGCDRLKRMAVSGDDCVVRPIDDRFGLALSHLNAMSKVRKDISEWQPSKGWNDWENVPFCSHHFHELQLKDGRR
IVVPCREQDELIGRGRVSPGNGWMIKETACLSKAYANMWSLMYFHKRDMRLLSLAVSSAVPTSWVPQGRTTWSIHGKGEWMTT
EDMLEVWNRVWITNNPHMQDKTMVKKWRDVPYLTKRQDKLCGSLIGMTNRATWASHIHLVIHRIRTLIGQEKYTDYLTVMDRY
SVDADLQLGELI
>Dengue1_LAV_TDV
MNNQRKKTGRPSFNMLKRARNRVSTVSQLAKRFSKGLLSGQGPMKLVMAFIAFLRFLAIPPTAGILARWGSFKKNGAIKVLRG
FKKEISNMLNIMNRRKRSVTMLLMLLPTALAFHLTTRGGEPHMIVSKQERGKSLLFKTSAGVNMCTLIAMDLGELCEDTMTYK
CPRITETEPDDVDCWCNATETWVTYGTCSQTGEHRRDKRSVALAPHVGLGLETRTETWMSSEGAWKQIQKVETWALRHPGFTV
IALFLAHAIGTSITQKGIIFILLMLVTPSMAMRCVGIGNRDFVEGLSGATWVDVVLEHGSCVTTMAKDKPTLDIELLKTEVTN
PAVLRKLCIEAKISNTTTDSRCPTQGEATLVEEQDTNFVCRRTFVDRGWGNGCGLFGKGSLITCAKFKCVTKLEGKIVQYENL
KYSVIVTVHTGDQHQVGNETTEHGTTATITPQAPTSEIQLTDYGALTLDCSPRTGLDFNEMVLLTMEKKSWLVHKQWFLDLPL
PWTSGASTSQETWNRQDLLVTFKTAHAKKQEVVVLGSQEGAMHTALTGATEIQSSGTTTIFAGHLKCRLKMDKLTLKGMSYVM
CTGSFKLEKEVAETQHGTVLVQVKYEGTDAPCKIPFSSQDEKGVTQNGRLITANPIVTDKEKPVNIEAEPPFGESYIVVGAGE
KALKLSWFKKGSSIGKMFEATARGARRMAILGDTAWDFGSIGGVFTSVGKLIHQIFGTAYGVLFSGVSWTMKIGIGILLTWLG
LNSRSTSLSMTCIAVGMVTLYLGVMVQADSGCVINWKGRELKCGSGIFVTNEVHTWTEQYKFQADSPKRLSAAIGKAWEEGVC
GIRSATRLENIMWKQISNELNHILLENDMKFTVVVGDVSGILAQGKKMIRPQPMEHKYSWKSWGKAKIIGADVQNTTFIIDGP
NTPECPDNQRAWNIWEVEDYGFGIFTTNIWLKLRDSYTQVCDHRLMSAAIKDSKAVHADMGYWIESEKNETWKLARASFIEVK
TCIWPKSHTLWSNGVLESEMIIPKIYGGPISQHNYRPGYFTQTAGPWHLGKLELDFDLCEGTTVVVDEHCGNRGPSLRTTTVT
GKTIHEWCCRSCTLPPLRFKGEDGCWYGMEIRPVKEKEENLVKSMVSAGSGEVDSFSLGLLCISIMIEEVMRSRWSRKMLMTG
TLAVFLLLTMGQLTWNDLIRLCIMVGANASDKMGMGTTYLALMATFRMRPMFAVGLLFRRLTSREVLLLTVGLSLVASVELPN
SLEELGDGLAMGIMMLKLLTDFQSHQLWATLLSLTFVKTTFSLHYAWKTMAMILSIVSLFPLCLSTTSQKTTWLPVLLGSLGC
KPLTMFLITENKIWGRKSWPLNEGIMAVGIVSILLSSLLKNDVPLAGPLIAGGMLIACYVISGSSADLSLEKAAEVSWEEEAE
HSGASHNILVEVQDDGTMKIKDEERDDTLTILLKATLLAISGVYPMSIPATLFVWYFWQKKKQRSGVLWDTPSPPEVERAVLD
DGIYRILQRGLLGRSQVGVGVFQEGVFHTMWHVTRGAVLMYQGKRLEPSWASVKKDLISYGGGWRFQGSWNAGEEVQVIAVEP

GKNPKNVQTAPGTFKTPEGEVGAIALDFKPGTSGSPIVNREGKIVGLYGNGVVTTSGTYVSAIAQAKASQEGPLPEIEDEVFR
KRNLTIMDLHPGSGKTRRYLPAIVREAIKRKLRTLVLAPTRVVASEMAEALKGMPIRYQTTAVKSEHTGKEIVDLMCHATFTM
RLLSPVRVPNYNMIIMDEAHFTDPASIAARGYISTRVGMGEAAAIFMTATPPGSVEAFPQSNAVIQDEERDIPERSWNSGYDW
ITDFPGKTVWFVPSIKSGNDIANCLRKNGKRVVQLSRKTFDTEYQKTKNNDWDYVVTTDISEMGANFRADRVIDPRRCLKPVI
LKDGPERVILAGPMPVTVASAAQRRGRIGRNQNKEGDQYIYMGQPLKNDEDHAHWTEAKMLLDNINTPEGIIPALFEPEREKS
AAIDGEYRLRGEARKTFVELMRRGDLPVWLSYKVASEGFQYSDRRWCFDGERNNQVLEENMDVEIWTKEGERKKLRPRWLDAR
TYSDPLALREFKEFAAGRRSVSGDLILEIGKLPQHLTQRAQNALDNLVMLHNSEQGGKAYRHAMEELPDTIETLMLLALIAVL
TGGVTLFFLSGRGLGKTSIGLLCVIASSALLWMASVEPHWIAASIILEFFLMVLLIPEPDRQRTPQDNQLAYVVIGLLFMILT
VAANEMGLLETTKKDLGIGHAAAENHHHAAMLDVDLHPASAWTLYAVATTIITPMMRHTIENTTANISLTAIANQAAILMGLD
KGWPISKMDIGVPLLALGCYSQVNPLTLTAAVFMLVAHYAIIGPGLQAKATREAQKRTAAGIMKNPTVDGIVAIDLDPVVYDA
KFEKQLGQIMLLILCTSQILLMRTTWALCESITLATGPLTTLWEGSPGKFWNTTIAVSMANIFRGSYLAGAGLAFSLMKSLGG
GRRGTGAQGETLGEKWKRQLNQLSKSEFNTYKRSGIIEVDRSEAKEGLKRGETTKHAVSRGTAKLRWFVERNLVKPEGKVIDL
GCGRGGWSYYCAGLKKVTEVKGYTKGGPGHEEPIPMATYGWNLVKLYSGKDVFFTPPEKCDTLLCDIGESSPNPTIEEGRTLR
VLKMVEPWLRGNQFCIKILNPYMPSVVETLEQMQRKHGGMLVRNPLSRNSTHEMYWVSCGTGNIVSAVNMTSRMLLNRFTMAH
RKPTYERDVDLGAGTRHVAVEPEVANLDIIGQRIENIKNEHKSTWHYDEDNPYKTWAYHGSYEVKPSGSASSMVNGVVRLLTK
PWDVIPMVTQIAMTDTTPFGQQRVFKEKVDTRTPKAKRGTAQIMEVTARWLWGFLSRNKKPRICTREEFTRKVRSNAAIGAVF
VDENQWNSAKEAVEDERFWDLVHRERELHKQGKCATCVYNMMGKREKKLGEFGKAKGSRAIWYMWLGARFLEFEALGFMNEDH
WFSRENSLSGVEGEGLHKLGYILRDISKIPGGNMYADDTAGWDTRITEDDLQNEAKITDIMEPEHALLATSIFKLTYQNKVVR
VQRPAKNGTVMDVISRRDQRGSGQVGTYGLNTFTNMEAQLIRQMESEGIFSPSELETPNLAERVLDWLKKHGTERLKRMAISG
DDCVVKPIDDRFATALTALNDMGKVRKDIPQWEPSKGWNDWQQVPFCSHHFHQLIMKDGREIVVPCRNQDELVGRARVSQGAG
WSLRETACLGKSYAQMWQLMYFHRRDLRLAANAICSAVPVDWVPTSRTTWSIHAHHQWMTTEDMLSVWNRVWIEENPWMEDKT
HVSSWEDVPYLGKREDQWCGSLIGLTARATWATNIQVAINQVRRLIGNENYLDFMTSMKRFKNESDPEGALW
>Dengue2_LAV_TDV
MNQRKKVVRPPFNMLKRERNRVSTPQGLVKRFSTGLFSGKGPLRMVLAFITFLRVLSIPPTAGILKRWGQLKKNKAIKILIGF
RKEIGRMLNILNGRKRSAGMIIMLIPTVMAFHLTTRNGEPHMIVSRQEKGKSLLFKTEDGVNMCTLMAMDLGELCEDTITYKC
PLLRQNEPEDIDCWCNSTSTWVTYGTCTTTGEHRREKRSVALVPHVGMGLETRTETWMSSEGAWKHAQRIETWILRHPGFTIM
AAILAYTIGTTHFQRALIFILLTAVAPSMTMRCIGISNRDFVEGVSGGSWVDIVLEHGSCVTTMAKNKPTLDFELIKTEAKQP
ATLRKYCIEAKLTNTTTESRCPTQGEPSLNEEQDKRFVCKHSMVDRGWGNGCGLFGKGGIVTCAMFTCKKNMEGKVVQPENLE
YTIVITPHSGEEHAVGNDTGKHGKEIKITPQSSITEAELTGYGTVTMECSPRTGLDFNEMVLLQMENKAWLVHRQWFLDLPLP
WLPGADTQGSNWIQKETLVTFKNPHAKKQDVVVLGSQEGAMHTALTGATEIQMSSGNLLFTGHLKCRLRMDKLQLKGMSYSMC
TGKFKVVKEIAETQHGTIVIRVQYEGDGSPCKIPFEIMDLEKRHVLGRLITVNPIVTEKDSPVNIEAEPPFGDSYIIIGVEPG
QLKLNWFKKGSSIGQMFETTMRGAKRMAILGDTAWDFGSLGGVFTSIGKALHQVFGAIYGAAFSGVSWTMKILIGVIITWIGM
NSRNTSMAMTCIAVGGITLFLGFTVQADMGCVASWSGKELKCGSGIFVVDNVHTWTEQYKFQPESPARLASAILNAHKDGVCG
IRSTTRLENVMWKQITNELNYVLWEGGHDLTVVAGDVKGVLTKGKRALTPPVSDLKYSWKTWGKAKIFTPEARNSTFLIDGPD
TSECPNERRAWNSLEVEDYGFGMFTTNIWMKFREGSSEVCDHRLMSAAIKDQKAVHADMGYWIESSKNQTWQIEKASLIEVKT
CLWPKTHTLWSNGVLESQMLIPKSYAGPFSQHNYRQGYATQTVGPWHLGKLEIDFGECPGTTVTIQEDCDHRGPSLRTTTASG
KLVTQWCCRSCTMPPLRFLGEDGCWYGMEIRPLSEKEENMVKSQVTAGQGTSETFSMGLLCLTLFVEECLRRRVTRKHMILVV
VITLCAIILGGLTWMDLLRALIMLGDTMSGRIGGQIHLAIMAVFKMSPGYVLGVFLRKLTSRETALMVIGMAMTTVLSIPHDL
MELIDGISLGLILLKIVTQFDNTQVGTLALSLTFIRSTMPLVMAWRTIMAVLFVVTLIPLCRTSCLQKQSHWVEITALILGAQ
ALPVYLMTLMKGASRRSWPLNEGIMAVGLVSLLGSALLKNDVPLAGPMVAGGLLLAAYVMSGSSADLSLEKAANVQWDEMADI
TGSSPIIEVKQDEDGSFSIRDVEETNMITLLVKLALITVSGLYPLAIPVTMTLWYMWQVKTQRSGALWDVPSPAATKKAALSE
GVYRIMQRGLFGKTQVGVGIHMEGVFHTMWHVTRGSVICHETGRLEPSWADVRNDMISYGGGWRLGDKWDKEEDVQVLAIEPG
KNPKHVQTKPGLFKTLTGEIGAVTLDFKPGTSGSPIINRKGKVIGLYGNGVVTKSGDYVSAITQAERIGEPDYEVDEDIFRKK
RLTIMDLHPGAGKTKRILPSIVREALKRRLRTLILAPTRVVAAEMEEALRGLPIRYQTPAVKSEHTGREIVDLMCHATFTTRL
LSSTRVPNYNLIVMDEAHFTDPSSVAARGYISTRVEMGEAAAIFMTATPPGATDPFPQSNSPIEDIEREIPERSWNTGFDWIT
DYQGKTVWFVPSIKAGNDIANCLRKSGKKVIQLSRKTFDTEYPKTKLTDWDFVVTTDISEMGANFRAGRVIDPRRCLKPVILP
DGPERVILAGPIPVTPASAAQRRGRIGRNPAQEDDQYVFSGDPLKNDEDHAHWTEAKMLLDNIYTPEGIIPTLFGPEREKTQA
IDGEFRLRGEQRKTFVELMRRGDLPVWLSYKVASAGISYKDREWCFTGERNNQILEENMEVEIWTREGEKKKLRPRWLDARVY

ADPMALKDFKEFASGRKSITLDILTEIASLPTYLSSRAKLALDNIVMLHTTERGGRAYQHALNELPESLETLMLVALLGAMTA
GIFLFFMQGKGIGKLSMGLITIAVASGLLWVAEIQPQWIAASIILEFFLMVLLIPEPEKQRTPQDNQLIYVILTILTIIGLIA
ANEMGLIEKTKTDFGFYQVKTETTILDVDLRPASAWTLYAVATTILTPMLRHTIENTSANLSLAAIANQAAVLMGLGKGWPLH
RMDLGVPLLAMGCYSQVNPTTLTASLVMLLVHYAIIGPGLQAKATREAQKRTAAGIMKNPTVDGITVIDLEPISYDPKFEKQL
GQVMLLVLCAGQLLLMRTTWAFCEVLTLATGPILTLWEGNPGRFWNTTIAVSTANIFRGSYLAGAGLAFSLIKNAQTPRRGTG
TTGETLGEKWKRQLNSLDRKEFEEYKRSGILEVDRTEAKSALKDGSKIKHAVSRGSSKIRWIVERGMVKPKGKVVDLGCGRGG
WSYYMATLKNVTEVKGYTKGGPGHEEPIPMATYGWNLVKLHSGVDVFYKPTEQVDTLLCDIGESSSNPTIEEGRTLRVLKMVE
PWLSSKPEFCIKVLNPYMPTVIEELEKLQRKHGGNLVRCPLSRNSTHEMYWVSGASGNIVSSVNTTSKMLLNRFTTRHRKPTY
EKDVDLGAGTRSVSTETEKPDMTIIGRRLQRLQEEHKETWHYDQENPYRTWAYHGSYEAPSTGSASSMVNGVVKLLTKPWDVI
PMVTQLAMTDTTPFGQQRVFKEKVDTRTPQPKPGTRMVMTTTANWLWALLGKKKNPRLCTREEFISKVRSNAAIGAVFQEEQG
WTSASEAVNDSRFWELVDKERALHQEGKCESCVYNMMGKREKKLGEFGRAKGSRAIWYMWLGARFLEFEALGFLNEDHWFGRE
NSWSGVEGEGLHRLGYILEEIDKKDGDLMYADDTAGWDTRITEDDLQNEELITEQMAPHHKILAKAIFKLTYQNKVVKVLRPT
PRGAVMDIISRKDQRGSGQVGTYGLNTFTNMEVQLIRQMEAEGVITQDDMQNPKGLKERVEKWLKECGVDRLKRMAISGDDCV
VKPLDERFGTSLLFLNDMGKVRKDIPQWEPSKGWKNWQEVPFCSHHFHKIFMKDGRSLVVPCRNQDELIGRARISQGAGWSLR
ETACLGKAYAQMWSLMYFHRRDLRLASMAICSAVPTEWFPTSRTTWSIHAHHQWMTTEDMLKVWNRVWIEDNPNMTDKTPVHS
WEDIPYLGKREDLWCGSLIGLSSRATWAKNIHTAITQVRNLIGKEEYVDYMPVMKRYSAPSESEGVL
>Dengue3_LAV_TDV
MNNQRKKTGKPSINMLKRVRNRVSTGSQLAKRFSRGLLNGQGPMKLVMAFIAFLRFLAIPPTAGVLARWGTFKKSGAIKVLRG
FKKEISNMLSIINRRKKTSLCLMMMLPATLAFHLTSRDGEPRMIVGKNERGKSLLFKTASGINMCTLIAMDLGEMCDDTVTYK
CPLITEVEPEDIDCWCNLTSTWVTYGTCNQAGEHRRDKRSVALAPHVGMGLDTRTQTWMSAEGAWRQVEKVETWAFRHPGFTI
LALFLAHYIGTSLTQKVVIFILLMLVTPSMTMRCVGVGNRDFVEGLSGATWVDVVLEHGGCVTTMAKNKPTLDIELQKTEATQ
LATLRKLCIEGKITNVTTDSRCPTQGEAILPEEQDQNHVCKHTYVDRGWGNGCGLFGKGSLVTCAKFQCLESIEGKVVQHENL
KYTVIITVHTGDQHQVGNETQGVTAEITPQASTVEAILPEYGTLGLECSPRTGLDFNEMILLTMKNKAWMVHRQWFFDLPLPW
TSGATTETPTWNKKELLVTFKNAHAKKQEVVVLGSQEGAMHTALTGATEIQTSGGTSIFAGHLKCRLKMDKLELKGMSYAMCL
NAFVLKKEVSETQHGTILIKVEYKGEDAPCKIPFSTEDGQGKAHNGRLITANPVVTKKEEPVNIEAEPPFGESNIVIGIGDKA
LKINWYKKGSSIGKMFEATARGARRMAILGDTAWDFGSVGGVLNSLGKMVHQIFGSAYTALFSGVSWIMKIGIGVLLTWIGLN
SKNTSMSFSCIVIGIITLYLGAVVQADMGCVINWKGKELKCGSGIFVTNEVHTWTEQYKFQADSPKRLATAIAGAWENGVCGI
RSTTRMENLLWKQIANELNYILWENNIKLTVVVGDIIGVLEQGKRTLTPQPMELKYSWKTWGKAKIVTAETQNSSFIIDGPNT
PECPSASRAWNVWEVEDYGFGVFTTNIWLKLREMYTQLCDHRLMSAAVKDERAVHADMGYWIESQKNGSWKLEKASLIEVKTC
TWPKSHTLWSNGVLESDMIIPKSLAGPISQHNYRPGYHTQTAGPWHLGKLELDFNYCEGTTVVITENCGTRGPSLRTTTVSGK
LIHEWCCRSCTLPPLRYMGEDGCWYGMEIRPINEKEENMVKSLVSAGSGKVDNFTMGVLCLAILFEEVMRGKFGKKHMIAGVL
FTFVLLLSGQITWRDMAHTLIMIGSNASDRMGMGVTYLALIATFKIQPFLALGFFLRKLTSRENLLLGVGLAMATTLQLPEDI
EQMANGIALGLMALKLITQFETYQLWTALVSLMCSNTIFTLTVAWRTATLILAGISLLPVCQSSSMRKTDWLPMAVAAMGVPP
LPLFIFSLKDTLKRRSWPLNEGVMAVGLVSILASSLLRNDVPMAGPLVAGGLLIACYVITGTSADLTVEKAADVTWEEEAEQT
GVSHNLMITVDDDGTMRIKDDETENILTVLLKTALLIVSGIFPYSIPATLLVWHTWQKQTQRSGVLWDVPSPPETQKAELEEG
VYRIKQQGIFGKTQVGVGVQKEGVFHTMWHVTRGAVLTHNGKRLEPNWASVKKDLISYGGGWKLSAQWQKGEEVQVIAVEPGK
NPKNFQTMPGIFQTTTGEIGAIALDFKPGTSGSPIINREGKVLGLYGNGVVTKNGGYVSGIAQTNAEPDGPTPELEEEMFKKR
NLTIMDLHPGSGKTRKYLPAIVREAIKRRLRTLILAPTRVVAAEMEEALKGLPIRYQTTATKSEHTGREIVDLMCHATFTMRL
LSPVRVPNYNLIIMDEAHFTDPASIAARGYISTRVGMGEAAAIFMTATPPGTADAFPQSNAPIQDEERDIPERSWNSGNEWIT
DFAGKTVWFVPSIKAGNDIANCLRKNGKKVIQLSRKTFDTEYQKTKLNDWDFVVTTDISEMGANFKADRVIDPRRCLKPVILT
DGPERVILAGPMPVTVASAAQRRGRVGRNPQKENDQYIFMGQPLNNDEDHAHWTEAKMLLDNINTPEGIIPALFEPEREKSAA
IDGEYRLKGESRKTFVELMRRGDLPVWLAHKVASEGIKYTDRKWCFDGERNNQILEENMDVEIWTKEGEKKKLRPRWLDARTY
SDPLALKEFKDFAAGRKSIALDLVTEIGRVPSHLAHRTRNALDNLVMLHTSEHGGRAYRHAVEELPETMETLLLLGLMILLTG
GAMLFLISGKGIGKTSIGLICVAASSGMLWMADVPLQWIASAIVLEFFMMVLLIPEPEKQRTPQDNQLAYVVIGILTLAAIVA
ANEMGLLETTKRDLGMSKEPGVVSPTSYLDVDLHPASAWTLYAVATTVITPMLRHTIENSTANVSLAAIANQAVVLMGLDKGW
PISKMDLGVPLLALGCYSQVNPLTLTAAVLLLVTHYAIIGPGLQAKATREAQKRTAAGIMKNPTVDGIMTIDLDPVIYDSKFE
KQLGQVMLLVLCAVQLLLMRTSWAFCEVLTLATGPITTLWEGSPGKFWNTTIAVSMANIFRGSYLAGAGLAFSIMKSVGTGKR
GTGSQGETLGEKWKKKLNQLPRKEFDLYKKSGITEVDRTEAKEGLKRGEITHHAVSRGSAKLQWFVERNMVIPEGRVIDLGCG

RGGWSYYCAGLKKVTEVRGYTKGGPGHEEPVPMSTYGWNIVKLMSGKDVFYLPPEKCDTLLCDIGESSPSPTVEESRTIRVLK
MVEPWLKNNQFCIKVLNPYMPTVIEHLERLQRKHGGMLVRNPLSRNSTHEMYWISNGTGNIVSSVNMVSRLLLNRFTMTHRRP
TIEKDVDLGAGTRHVNAEPETPNMDVIGERIRRIKEEHSSTWHYDDENPYKTWAYHGSYEVKATGSASSMINGVVKLLTKPWD
VVPMVTQMAMTDTTPFGQQRVFKEKVDTRTPRPMPGTRKVMEITAEWLWRTLGRNKRPRLCTREEFTKKVRTNAAMGAVFTEE
NQWDSARAAVEDEEFWKLVDRERELHKLGKCGSCVYNMMGKREKKLGEFGKAKGSRAIWYMWLGARYLEFEALGFLNEDHWFS
RENSYSGVEGEGLHKLGYILRDISKIPGGAMYADDTAGWDTRITEDDLHNEEKITQQMDPEHRQLANAIFKLTYQNKVVKVQR
PTPKGTVMDIISRKDQRGSGQVGTYGLNTFTNMEAQLIRQMEGEGVLSKADLENPHLLEKKVTQWLETKGVERLKRMAISGDD
CVVKPIDDRFANALLALNDMGKVRKDIPQWQPSKGWHDWQQVPFCSHHFHELIMKDGRKLVVPCRPQDELIGRARISQGAGWS
LRETACLGKAYAQMWTLMYFHRRDLRLASNAICSAVPVHWVPTSRTTWSIHAHHQWMTTEDMLTVWNRVWIEDNPWMEDKTPV
KTWEDVPYLGKREDQWCGSLIGLTSRATWAQNILTAIQQVRSLIGNEEFLDYMPSMKRFRKEEESEGAIW
>Dengue4_LAV_TDV
MNQRKKVVRPPFNMLKRERNRVSTPQGLVKRFSTGLFSGKGPLRMVLAFITFLRVLSIPPTAGILKRWGQLKKNKAIKILIGF
RKEIGRMLNILNGRKRSTITLLCLIPTVMAFSLSTRDGEPLMIVAKHERGRPLLFKTTEGINKCTLIAMDLGEMCEDTVTYKC
PLLVNTEPEDIDCWCNLTSTWVMYGTCTQSGERRREKRSVALTPHSGMGLETRAETWMSSEGAWKHAQRVESWILRNPGFALL
AGFMAYMIGQTGIQRTVFFVLMMLVAPSYGMRCVGVGNRDFVEGVSGGAWVDLVLEHGGCVTTMAQGKPTLDFELTKTTAKEV
ALLRTYCIEASISNITTATRCPTQGEPYLKEEQDQQYICRRDVVDRGWGNGCGLFGKGGVVTCAKFSCSGKITGNLVQIENLE
YTVVVTVHNGDTHAVGNDTSNHGVTAMITPRSPSVEVKLPDYGELTLDCEPRSGIDFNEMILMKMKKKTWLVHKQWFLDLPLP
WTAGADTSEVHWNYKERMVTFKVPHAKRQDVTVLGSQEGAMHSALAGATEVDSGDGNHMFAGHLKCKVRMEKLRIKGMSYTMC
SGKFSIDKEMAETQHGTTVVKVKYEGAGAPCKVPIEIRDVNKEKVVGRIISSTPLAENTNSVTNIELEPPFGDSYIVIGVGNS
ALTLHWFRKGSSIGKMFESTYRGAKRMAILGETAWDFGSVGGLFTSLGKAVHQVFGSVYTTMFGGVSWMIRILIGFLVLWIGT
NSRNTSMAMTCIAVGGITLFLGFTVQADMGCVASWSGKELKCGSGIFVVDNVHTWTEQYKFQPESPARLASAILNAHKDGVCG
IRSTTRLENVMWKQITNELNYVLWEGGHDLTVVAGDVKGVLTKGKRALTPPVSDLKYSWKTWGKAKIFTPEARNSTFLIDGPD
TSECPNERRAWNSLEVEDYGFGMFTTNIWMKFREGSSEVCDHRLMSAAIKDQKAVHADMGYWIESSKNQTWQIEKASLIEVKT
CLWPKTHTLWSNGVLESQMLIPKSYAGPFSQHNYRQGYATQTVGPWHLGKLEIDFGECPGTTVTIQEDCDHRGPSLRTTTASG
KLVTQWCCRSCTMPPLRFLGEDGCWYGMEIRPLSEKEENMVKSQVTAGQGTSETFSMGLLCLTLFVEECLRRRVTRKHMILVV
VITLCAIILGGLTWMDLLRALIMLGDTMSGRIGGQIHLAIMAVFKMSPGYVLGVFLRKLTSRETALMVIGMAMTTVLSIPHDL
MELIDGISLGLILLKIVTQFDNTQVGTLALSLTFIRSTMPLVMAWRTIMAVLFVVTLIPLCRTSCLQKQSHWVEITALILGAQ
ALPVYLMTLMKGASRRSWPLNEGIMAVGLVSLLGSALLKNDVPLAGPMVAGGLLLAAYVMSGSSADLSLEKAANVQWDEMADI
TGSSPIIEVKQDEDGSFSIRDVEETNMITLLVKLALITVSGLYPLAIPVTMTLWYMWQVKTQRSGALWDVPSPAATKKAALSE
GVYRIMQRGLFGKTQVGVGIHMEGVFHTMWHVTRGSVICHETGRLEPSWADVRNDMISYGGGWRLGDKWDKEEDVQVLAIEPG
KNPKHVQTKPGLFKTLTGEIGAVTLDFKPGTSGSPIINRKGKVIGLYGNGVVTKSGDYVSAITQAERIGEPDYEVDEDIFRKK
RLTIMDLHPGAGKTKRILPSIVREALKRRLRTLILAPTRVVAAEMEEALRGLPIRYQTPAVKSEHTGREIVDLMCHATFTTRL
LSSTRVPNYNLIVMDEAHFTDPSSVAARGYISTRVEMGEAAAIFMTATPPGATDPFPQSNSPIEDIEREIPERSWNTGFDWIT
DYQGKTVWFVPSIKAGNDIANCLRKSGKKVIQLSRKTFDTEYPKTKLTDWDFVVTTDISEMGANFRAGRVIDPRRCLKPVILP
DGPERVILAGPIPVTPASAAQRRGRIGRNPAQEDDQYVFSGDPLKNDEDHAHWTEAKMLLDNIYTPEGIIPTLFGPEREKTQA
IDGEFRLRGEQRKTFVELMRRGDLPVWLSYKVASAGISYKDREWCFTGERNNQILEENMEVEIWTREGEKKKLRPRWLDARVY
ADPMALKDFKEFASGRKSITLDILTEIASLPTYLSSRAKLALDNIVMLHTTERGGRAYQHALNELPESLETLMLVALLGAMTA
GIFLFFMQGKGIGKLSMGLITIAVASGLLWVAEIQPQWIAASIILEFFLMVLLIPEPEKQRTPQDNQLIYVILTILTIIGLIA
ANEMGLIEKTKTDFGFYQVKTETTILDVDLRPASAWTLYAVATTILTPMLRHTIENTSANLSLAAIANQAAVLMGLGKGWPLH
RMDLGVPLLAMGCYSQVNPTTLTASLVMLFVHYAIIGPGLQAKATREAQKRTAAGIMKNPTVDGITVIDLEPISYDPKFEKQL
GQVMLLVLCAGQLLLMRTTWAFCEVLTLATGPILTLWEGNPGRFWNTTIAVSTANIFRGSYLAGAGLAFSLIKNAQTPRRGTG
TTGETLGEKWKRQLNSLDRKEFEEYKRSGILEVDRTEAKSALKDGSKIKHAVSRGSSKIRWIVERGMVKPKGKVVDLGCGRGG
WSYYMATLKNVTEVKGYTKGGPGHEEPIPMATYGWNLVKLHSGVDVFYKPTEQVDTLLCDIGESSSNPTIEEGRTLRVLKMVE
PWLSSKPEFCIKVLNPYMPTVIEELEKLQRKHGGNLVRCPLSRNSTHEMYWVSGASGNIVSSVNTTSKMLLNRFTTRHRKPTY
EKDVDLGAGTRSVSTETEKPDMTIIGRRLQRLQEEHKETWHYDQENPYRTWAYHGSYEAPSTGSASSMVNGVVKLLTKPWDVI
PMVTQLAMTDTTPFGQQRVFKEKVDTRTPQPKPGTRMVMTTTANWLWALLGKKKNPRLCTREEFISKVRSNAAIGAVFQEEQG
WTSASEAVNDSRFWELVDKERALHQEGKCESCVYNMMGKREKKLGEFGRAKGSRAIWYMWLGARFLEFEALGFLNEDHWFGRE
NSWSGVEGEGLHRLGYILEEIDKKDGDLMYADDTAGWDTRITEDDLQNEELITEQMAPHHKILAKAIFKLTYQNKVVKVLRPT

PRGAVMDIISRKDQRGSGQVGTYGLNTFTNMEVQLIRQMEAEGVITQDDMQNPKGLKERVEKWLKECGVDRLKRMAISGDDCV
VKPLDERFGTSLLFLNDMGKVRKDIPQWEPSKGWKNWQEVPFCSHHFHKIFMKDGRSLVVPCRNQDELIGRARISQGAGWSLR
ETACLGKAYAQMWSLMYFHRRDLRLASMAICSAVPTEWFPTSRTTWSIHAHHQWMTTEDMLKVWNRVWIEDNPNMTDKTPVHS
WEDIPYLGKREDLWCGSLIGLSSRATWAKNIHTAITQVRNLIGKEEYVDYMPVMKRYSAPSESEGVL
>Dengue1_TAK_003
MNNQRKKAKNTPFNMLKRERNRVSTVQQLTKRFSLGMLQGRGPLKLFMALVAFLRFLTIPPTAGILKRWGTIKKSKAINVLRG
FRKEIGRMLNILNRRRRSAGMIIMLIPTVMAFHLTTRGGEPHMIVSKQERGKSLLFKTSAGVNMCTLIAMDLGELCEDTMTYK
CPRITEAEPDDVDCWCNATDTWVTYGTCSQTGEHRRDKRSVALAPHVGLGLETRAETWMSSEGAWKQIQKVETWALRHPGFTV
IALFLAHAIGTSITQKGIIFILLMLVTPSMAMRCVGIGNRDFVEGLSGATWVDVVLEHGSCVTTMAKNKPTLDIELLKTEVTN
PAVLRKLCIEAKISNTTTDSRCPTQGEATLVEEQDANFVCRRTFVDRGWGNGCGLFGKGSLITCAKFKCVTKLEGKIVQYENL
KYSVIVTVHTGDQHQVGNETTEHGTTATITPQAPTSEIQLTDYGTLTLDCSPRTGLDFNEMVLLTMKERSWLVHKQWFLDLPL
PWTSGASTSQETWNRQDLLVTFKTAHAKKQEVVVLGSQEGAMHTALTGATEIQTSGTTTIFAGHLKCRLKMDKLTLKGMSYVM
CTGSFKLEKEVAETQHGTVLVQVKYEGTDAPCKIPFSTQDEKGATQNGRLITANPIVTDKEKPVNIEAEPPFGESYIVVGAGE
KALKLSWFKKGSSIGKMFEATARGARRMAILGDTAWDFGSIGGVFTSMGKLVHQVFGTAYGVLFSGVSWTMKIGIGILLTWLG
LNSRNTSLSMMCIAVGMVTLYLGVMVQADSGCVVSWKNKELKCGSGIFITDNVHTWTEQYKFQPESPSKLASAIQKAHEEDIC
GIRSVTRLENLMWKQITPELNHILSENEVKLTIMTGDIKGIMQAGKRSLRPQPTELKYSWKTWGKAKMLSTESHNQTFLIDGP
ETAECPNTNRAWNSLEVEDYGFGVFTTNIWLKLKEKQDVFCDSKLMSAAIKDNRAVHADMGYWIESALNDTWKIEKASFIEVK
NCHWPKSHTLWSNGVLESEMIIPKNLAGPVSQHNYRPGYHTQITGPWHLGKLEMDFDFCDGTTVVVTEDCGNRGPSLRTTTAS
GKLITEWCCRSCTLPPLRYRGEDGCWYGMEIRPLKEKEENLVNSLVTAGHGQVDNFSLGVLGMALFLEEMLRTRVGTKHAILL
VAVSFVTLITGNMSFRDLGRVMVMVGATMTDDIGMGVTYLALLAAFKVRPTFAAGLLLRKLTSKELMMTTIGIVLLSQSTIPE
TILELTDALALGMMVLKMVRNMEKYQLAVTIMAILCVPNAVILQNAWKVSCTILAVVSVSPLFLTSSQQKTDWIPLALTIKGL
NPTAIFLTTLSRTSKKRSWPLNEAIMAVGMVSILASSLLKNDIPMTGPLVAGGLLTVCYVLTGRSADLELERAADVKWEDQAE
ISGSSPILSITISEDGSMSIKNEEEEQTLTILIRTGLLVISGLFPVSIPITAAAWYLWEVKKQRAGVLWDVPSPPPMGKAELE
DGAYRIKQKGILGYSQIGAGVYKEGTFHTMWHVTRGAVLMHKGKRIEPSWADVKKDLISYGGGWKLEGEWKEGEEVQVLALEP
GKNPRAVQTKPGLFKTNAGTIGAVSLDFSPGTSGSPIIDKKGKVVGLYGNGVVTRSGAYVSAIAQTEKSIEDNPEIEDDIFRK
RRLTIMDLHPGAGKTKRYLPAIVREAIKRGLRTLILAPTRVVAAEMEEALRGLPIRYQTPAIRAVHTGREIVDLMCHATFTMR
LLSPVRVPNYNLIIMDEAHFTDPASIAARGYISTRVEMGEAAGIFMTATPPGSRDPFPQSNAPIIDEEREIPERSWNSGHEWV
TDFKGKTVWFVPSIKAGNDIAACLRKNGKKVIQLSRKTFDSEYVKTRTNDWDFVVTTDISEMGANFKAERVIDPRRCMKPVIL
TDGEERVILAGPMPVTHSSAAQRRGRIGRNPKNENDQYIYMGEPLENDEDCAHWKEAKMLLDNINTPEGIIPSMFEPEREKVD
AIDGEYRLRGEARKTFVDLMRRGDLPVWLAYRVAAEGINYADRRWCFDGVKNNQILEENVEVEIWTKEGERKKLKPRWLDARI
YSDPLALKEFKEFAAGRKSLTLNLITEMGRLPTFMTQKARDALDNLAVLHTAEAGGRAYNHALSELPETLETLLLLTLLATVT
GGIFLFLMSARGIGKMTLGMCCIITASILLWYAQIQPHWIAASIILEFFLIVLLIPEPEKQRTPQDNQLTYVVIAILTVVAAT
MANEMGFLEKTKKDLGLGSIATQQPESNILDIDLRPASAWTLYAVATTFVTPMLRHSIENSSVNVSLTAIANQATVLMGLGKG
WPLSKMDIGVPLLAIGCYSQVNPITLTAALFLLVAHYAIIGPGLQAKATREAQKRAAAGIMKNPTVDGITVIDLDPIPYDPKF
EKQLGQVMLLVLCVTQVLMMRTTWALCEALTLATGPISTLWEGNPGRFWNTTIAVSMANIFRGSYLAGAGLLFSIMKNTTNTR
RGTGNIGETLGEKWKSRLNALGKSEFQIYKKSGIQEVDRTLAKEGIKRGETDHHAVSRGSAKLRWFVERNMVTPEGKVVDLGC
GRGGWSYYCGGLKNVREVKGLTKGGPGHEEPIPMSTYGWNLVRLQSGVDVFFIPPEKCDTLLCDIGESSPNPTVEAGRTLRVL
NLVENWLNNNTQFCIKVLNPYMPSVIEKMEALQRKYGGALVRNPLSRNSTHEMYWVSNASGNIVSSVNMISRMLINRFTMRYK
KATYEPDVDLGSGTRNIGIESEIPNLDIIGKRIEKIKQEHETSWHYDQDHPYKTWAYHGSYETKQTGSASSMVNGVVRLLTKP
WDVVPMVTQMAMTDTTPFGQQRVFKEKVDTRTQEPKEGTKKLMKITAEWLWKELGKKKTPRMCTREEFTRKVRSNAALGAIFT
DENKWKSAREAVEDSRFWELVDKERNLHLEGKCETCVYNMMGKREKKLGEFGKAKGSRAIWYMWLGARFLEFEALGFLNEDHW
FSRENSLSGVEGEGLHKLGYILRDVSKKEGGAMYADDTAGWDTRITLEDLKNEEMVTNHMEGEHKKLAEAIFKLTYQNKVVRV
QRPTPRGTVMDIISRRDQRGSGQVGTYGLNTFTNMEAQLIRQMEGEGVFKSIQHLTITEEIAVQNWLARVGRERLSRMAISGD
DCVVKPLDDRFASALTALNDMGKIRKDIQQWEPSRGWNDWTQVPFCSHHFHELIMKDGRVLVVPCRNQDELIGRARISQGAGW
SLRETACLGKSYAQMWSLMYFHRRDLRLAANAICSAVPSHWVPTSRTTWSIHAKHEWMTTEDMLTVWNRVWIQENPWMEDKTP
VESWEEIPYLGKREDQWCGSLIGLTSRATWAKNIQAAINQVRSLIGNEEYTDYMPSMKRFRREEEEAGVLW
>Dengue2_TAK_003
MNNQRKKAKNTPFNMLKRERNRVSTVQQLTKRFSLGMLQGRGPLKLFMALVAFLRFLTIPPTAGILKRWGTIKKSKAINVLRG

FRKEIGRMLNILNRRRRSAGMIIMLIPTVMAFHLTTRNGEPHMIVSRQEKGKSLLFKTEVGVNMCTLMAMDLGELCEDTITYK
CPLLRQNEPEDIDCWCNSTSTWVTYGTCTTMGEHRREKRSVALVPHVGMGLETRTETWMSSEGAWKHVQRIETWILRHPGFTM
MAAILAYTIGTTHFQRALIFILLTAVTPSMTMRCIGMSNRDFVEGVSGGSWVDIVLEHGSCVTTMAKNKPTLDFELIKTEAKQ
PATLRKYCIEAKLTNTTTESRCPTQGEPSLNEEQDKRFVCKHSMVDRGWGNGCGLFGKGGIVTCAMFRCKKNMEGKVVQPENL
EYTIVITPHSGEEHAVGNDTGKHGKEIKITPQSSITEAELTGYGTVTMECSPRTGLDFNEMVLLQMENKAWLVHRQWFLDLPL
PWLPGADTQGSNWIQKETLVTFKNPHAKKQDVVVLGSQEGAMHTALTGATEIQMSSGNLLFTGHLKCRLRMDKLQLKGMSYSM
CTGKFKVVKEIAETQHGTIVIRVQYEGDGSPCKIPFEIMDLEKRHVLGRLITVNPIVTEKDSPVNIEAEPPFGDSYIIIGVEP
GQLKLNWFKKGSSIGQMFETTMRGAKRMAILGDTAWDFGSLGGVFTSIGKALHQVFGAIYGAAFSGVSWTMKILIGVIITWIG
MNSRSTSLSVTLVLVGIVTLYLGVMVQADSGCVVSWKNKELKCGSGIFITDNVHTWTEQYKFQPESPSKLASAIQKAHEEDIC
GIRSVTRLENLMWKQITPELNHILSENEVKLTIMTGDIKGIMQAGKRSLRPQPTELKYSWKTWGKAKMLSTESHNQTFLIDGP
ETAECPNTNRAWNSLEVEDYGFGVFTTNIWLKLKEKQDVFCDSKLMSAAIKDNRAVHADMGYWIESALNDTWKIEKASFIEVK
NCHWPKSHTLWSNGVLESEMIIPKNLAGPVSQHNYRPGYHTQITGPWHLGKLEMDFDFCDGTTVVVTEDCGNRGPSLRTTTAS
GKLITEWCCRSCTLPPLRYRGEDGCWYGMEIRPLKEKEENLVNSLVTAGHGQVDNFSLGVLGMALFLEEMLRTRVGTKHAILL
VAVSFVTLITGNMSFRDLGRVMVMVGATMTDDIGMGVTYLALLAAFKVRPTFAAGLLLRKLTSKELMMTTIGIVLLSQSTIPE
TILELTDALALGMMVLKMVRNMEKYQLAVTIMAILCVPNAVILQNAWKVSCTILAVVSVSPLFLTSSQQKTDWIPLALTIKGL
NPTAIFLTTLSRTSKKRSWPLNEAIMAVGMVSILASSLLKNDIPMTGPLVAGGLLTVCYVLTGRSADLELERAADVKWEDQAE
ISGSSPILSITISEDGSMSIKNEEEEQTLTILIRTGLLVISGLFPVSIPITAAAWYLWEVKKQRAGVLWDVPSPPPMGKAELE
DGAYRIKQKGILGYSQIGAGVYKEGTFHTMWHVTRGAVLMHKGKRIEPSWADVKKDLISYGGGWKLEGEWKEGEEVQVLALEP
GKNPRAVQTKPGLFKTNAGTIGAVSLDFSPGTSGSPIIDKKGKVVGLYGNGVVTRSGAYVSAIAQTEKSIEDNPEIEDDIFRK
RRLTIMDLHPGAGKTKRYLPAIVREAIKRGLRTLILAPTRVVAAEMEEALRGLPIRYQTPAIRAVHTGREIVDLMCHATFTMR
LLSPVRVPNYNLIIMDEAHFTDPASIAARGYISTRVEMGEAAGIFMTATPPGSRDPFPQSNAPIIDEEREIPERSWNSGHEWV
TDFKGKTVWFVPSIKAGNDIAACLRKNGKKVIQLSRKTFDSEYVKTRTNDWDFVVTTDISEMGANFKAERVIDPRRCMKPVIL
TDGEERVILAGPMPVTHSSAAQRRGRIGRNPKNENDQYIYMGEPLENDEDCAHWKEAKMLLDNINTPEGIIPSMFEPEREKVD
AIDGEYRLRGEARKTFVDLMRRGDLPVWLAYRVAAEGINYADRRWCFDGVKNNQILEENVEVEIWTKEGERKKLKPRWLDARI
YSDPLALKEFKEFAAGRKSLTLNLITEMGRLPTFMTQKARDALDNLAVLHTAEAGGRAYNHALSELPETLETLLLLTLLATVT
GGIFLFLMSARGIGKMTLGMCCIITASILLWYAQIQPHWIAASIILEFFLIVLLIPEPEKQRTPQDNQLTYVVIAILTVVAAT
MANEMGFLEKTKKDLGLGSIATQQPESNILDIDLRPASAWTLYAVATTFVTPMLRHSIENSSVNVSLTAIANQATVLMGLGKG
WPLSKMDIGVPLLAIGCYSQVNPITLTAALFLLVAHYAIIGPGLQAKATREAQKRAAAGIMKNPTVDGITVIDLDPIPYDPKF
EKQLGQVMLLVLCVTQVLMMRTTWALCEALTLATGPISTLWEGNPGRFWNTTIAVSMANIFRGSYLAGAGLLFSIMKNTTNTR
RGTGNIGETLGEKWKSRLNALGKSEFQIYKKSGIQEVDRTLAKEGIKRGETDHHAVSRGSAKLRWFVERNMVTPEGKVVDLGC
GRGGWSYYCGGLKNVREVKGLTKGGPGHEEPIPMSTYGWNLVRLQSGVDVFFIPPEKCDTLLCDIGESSPNPTVEAGRTLRVL
NLVENWLNNNTQFCIKVLNPYMPSVIEKMEALQRKYGGALVRNPLSRNSTHEMYWVSNASGNIVSSVNMISRMLINRFTMRYK
KATYEPDVDLGSGTRNIGIESEIPNLDIIGKRIEKIKQEHETSWHYDQDHPYKTWAYHGSYETKQTGSASSMVNGVVRLLTKP
WDVVPMVTQMAMTDTTPFGQQRVFKEKVDTRTQEPKEGTKKLMKITAEWLWKELGKKKTPRMCTREEFTRKVRSNAALGAIFT
DENKWKSAREAVEDSRFWELVDKERNLHLEGKCETCVYNMMGKREKKLGEFGKAKGSRAIWYMWLGARFLEFEALGFLNEDHW
FSRENSLSGVEGEGLHKLGYILRDVSKKEGGAMYADDTAGWDTRITLEDLKNEEMVTNHMEGEHKKLAEAIFKLTYQNKVVRV
QRPTPRGTVMDIISRRDQRGSGQVGTYGLNTFTNMEAQLIRQMEGEGVFKSIQHLTITEEIAVQNWLARVGRERLSRMAISGD
DCVVKPLDDRFASALTALNDMGKIRKDIQQWEPSRGWNDWTQVPFCSHHFHELIMKDGRVLVVPCRNQDELIGRARISQGAGW
SLRETACLGKSYAQMWSLMYFHRRDLRLAANAICSAVPSHWVPTSRTTWSIHAKHEWMTTEDMLTVWNRVWIQENPWMEDKTP
VESWEEIPYLGKREDQWCGSLIGLTSRATWAKNIQAAINQVRSLIGNEEYTDYMPSMKRFRREEEEAGVLW
>Dengue3_TAK_003
MNNQRKKAKNTPFNMLKRERNRVSTVQQLTKRFSLGMLQGRGPLKLFMALVAFLRFLTIPPTAGILKRWGTIKKSKAINVLRG
FRKEIGRMLNILNRRRRSAGMIIMLIPTVMAFHLTSRDGEPRMIVGKNERGKSLLFKTASGINMCTLIAMDLGEMCDDTVTYK
CPHITEVEPEDIDCWCNLTSTWVTYGTCNQAGERRRDKRSVALAPHVGMGLDTRTQTWMSAEGAWRQVEKVETWALRHPGFTI
LALFLAHYIGTSLTQKVVIFILLMLVTPSMTMRCVGVGNRDFVEGLSGATWVDVVLEHGGCVTTMAKNKPTLDIELQKTEATQ
LATLRKLCIEGKITNITTDSRCPTQGEAVLPEEQDQNYVCKHTYVDRGWGNGCGLFGKGSLVTCAKFQCLEPIEGKVVQYENL
KYTVIITVHTGDQHQVGNETQGVTAEITPQASTTEAILPEYGTLGLECSPRTGLDFNEMILLTMKNKAWMVHRQWFFDLPLPW
TSGATTETPTWNRKELLVTFKNAHAKKQEVVVLGSQEGAMHTALTGATEIQNSGGTSIFAGHLKCRLKMDKLELKGMSYAMCT

NTFVLKKEVSETQHGTILIKVEYKGEDAPCKIPFSTEDGQGKAHNGRLITANPVVTKKEEPVNIEAEPPFGESNIVIGIGDNA
LKINWYKKGSSIGKMFEATARGARRMAILGDTAWDFGSVGGVLNSLGKMVHQIFGSAYTALFSGVSWVMKIGIGVLLTWIGLN
SKNTSMSFSCIAIGIITLYLGAVVQADSGCVVSWKNKELKCGSGIFITDNVHTWTEQYKFQPESPSKLASAIQKAHEEDICGI
RSVTRLENLMWKQITPELNHILSENEVKLTIMTGDIKGIMQAGKRSLRPQPTELKYSWKTWGKAKMLSTESHNQTFLIDGPET
AECPNTNRAWNSLEVEDYGFGVFTTNIWLKLKEKQDVFCDSKLMSAAIKDNRAVHADMGYWIESALNDTWKIEKASFIEVKNC
HWPKSHTLWSNGVLESEMIIPKNLAGPVSQHNYRPGYHTQITGPWHLGKLEMDFDFCDGTTVVVTEDCGNRGPSLRTTTASGK
LITEWCCRSCTLPPLRYRGEDGCWYGMEIRPLKEKEENLVNSLVTAGHGQVDNFSLGVLGMALFLEEMLRTRVGTKHAILLVA
VSFVTLITGNMSFRDLGRVMVMVGATMTDDIGMGVTYLALLAAFKVRPTFAAGLLLRKLTSKELMMTTIGIVLLSQSTIPETI
LELTDALALGMMVLKMVRNMEKYQLAVTIMAILCVPNAVILQNAWKVSCTILAVVSVSPLFLTSSQQKTDWIPLALTIKGLNP
TAIFLTTLSRTSKKRSWPLNEAIMAVGMVSILASSLLKNDIPMTGPLVAGGLLTVCYVLTGRSADLELERAADVKWEDQAEIS
GSSPILSITISEDGSMSIKNEEEEQTLTILIRTGLLVISGLFPVSIPITAAAWYLWEVKKQRAGVLWDVPSPPPMGKAELEDG
AYRIKQKGILGYSQIGAGVYKEGTFHTMWHVTRGAVLMHKGKRIEPSWADVKKDLISYGGGWKLEGEWKEGEEVQVLALEPGK
NPRAVQTKPGLFKTNAGTIGAVSLDFSPGTSGSPIIDKKGKVVGLYGNGVVTRSGAYVSAIAQTEKSIEDNPEIEDDIFRKRR
LTIMDLHPGAGKTKRYLPAIVREAIKRGLRTLILAPTRVVAAEMEEALRGLPIRYQTPAIRAVHTGREIVDLMCHATFTMRLL
SPVRVPNYNLIIMDEAHFTDPASIAARGYISTRVEMGEAAGIFMTATPPGSRDPFPQSNAPIIDEEREIPERSWNSGHEWVTD
FKGKTVWFVPSIKAGNDIAACLRKNGKKVIQLSRKTFDSEYVKTRTNDWDFVVTTDISEMGANFKAERVIDPRRCMKPVILTD
GEERVILAGPMPVTHSSAAQRRGRIGRNPKNENDQYIYMGEPLENDEDCAHWKEAKMLLDNINTPEGIIPSMFEPEREKVDAI
DGEYRLRGEARKTFVDLMRRGDLPVWLAYRVAAEGINYADRRWCFDGVKNNQILEENVEVEIWTKEGERKKLKPRWLDARIYS
DPLALKEFKEFAAGRKSLTLNLITEMGRLPTFMTQKARDALDNLAVLHTAEAGGRAYNHALSELPETLETLLLLTLLATVTGG
IFLFLMSARGIGKMTLGMCCIITASILLWYAQIQPHWIAASIILEFFLIVLLIPEPEKQRTPQDNQLTYVVIAILTVVAATMA
NEMGFLEKTKKDLGLGSIATQQPESNILDIDLRPASAWTLYAVATTFVTPMLRHSIENSSVNVSLTAIANQATVLMGLGKGWP
LSKMDIGVPLLAIGCYSQVNPITLTAALFLLVAHYAIIGPGLQAKATREAQKRAAAGIMKNPTVDGITVIDLDPIPYDPKFEK
QLGQVMLLVLCVTQVLMMRTTWALCEALTLATGPISTLWEGNPGRFWNTTIAVSMANIFRGSYLAGAGLLFSIMKNTTNTRRG
TGNIGETLGEKWKSRLNALGKSEFQIYKKSGIQEVDRTLAKEGIKRGETDHHAVSRGSAKLRWFVERNMVTPEGKVVDLGCGR
GGWSYYCGGLKNVREVKGLTKGGPGHEEPIPMSTYGWNLVRLQSGVDVFFIPPEKCDTLLCDIGESSPNPTVEAGRTLRVLNL
VENWLNNNTQFCIKVLNPYMPSVIEKMEALQRKYGGALVRNPLSRNSTHEMYWVSNASGNIVSSVNMISRMLINRFTMRYKKA
TYEPDVDLGSGTRNIGIESEIPNLDIIGKRIEKIKQEHETSWHYDQDHPYKTWAYHGSYETKQTGSASSMVNGVVRLLTKPWD
VVPMVTQMAMTDTTPFGQQRVFKEKVDTRTQEPKEGTKKLMKITAEWLWKELGKKKTPRMCTREEFTRKVRSNAALGAIFTDE
NKWKSAREAVEDSRFWELVDKERNLHLEGKCETCVYNMMGKREKKLGEFGKAKGSRAIWYMWLGARFLEFEALGFLNEDHWFS
RENSLSGVEGEGLHKLGYILRDVSKKEGGAMYADDTAGWDTRITLEDLKNEEMVTNHMEGEHKKLAEAIFKLTYQNKVVRVQR
PTPRGTVMDIISRRDQRGSGQVGTYGLNTFTNMEAQLIRQMEGEGVFKSIQHLTITEEIAVQNWLARVGRERLSRMAISGDDC
VVKPLDDRFASALTALNDMGKIRKDIQQWEPSRGWNDWTQVPFCSHHFHELIMKDGRVLVVPCRNQDELIGRARISQGAGWSL
RETACLGKSYAQMWSLMYFHRRDLRLAANAICSAVPSHWVPTSRTTWSIHAKHEWMTTEDMLTVWNRVWIQENPWMEDKTPVE
SWEEIPYLGKREDQWCGSLIGLTSRATWAKNIQAAINQVRSLIGNEEYTDYMPSMKRFRREEEEAGVLW
>Dengue4_TAK_003
MNNQRKKAKNTPFNMLKRERNRVSTVQQLTKRFSLGMLQGRGPLKLFMALVAFLRFLTIPPTAGILKRWGTIKKSKAINVLRG
FRKEIGRMLNILNRRRRSAGMIIMLIPTVMAFHLTTRNGEPHMIVSRQEKGKSLLFKTEVGVNMCTLMAMDLGELCEDTITYK
CPLLRQNEPEDIDCWCNSTSTWVTYGTCTTMGEHRREKRSVALVPHVGMGLETRTETWMSSEGAWKHVQRIETWILRHPGFTM
MAAILAYTIGTTHFQRALIFILLTAVTPSMTMRCVGVGNRDFVEGVSGGAWVDLVLEHGGCVTTMAQGKPTLDFELTKTTAKE
VALLRTYCIEASISNITTATRCPTQGEPYLKEEQDQQYICRRDVVDRGWGNGCGLFGKGGVVTCAKFSCSGKITGNLVQIENL
EYTVVVTVHNGDTHAVGNDTSNHGVTATITPRSPSVEVKLPDYGELTLDCEPRSGIDFNEMILMKMKKKTWLVHKQWFLDLPL
PWTAGADTSEVHWNYKERMVTFKVPHAKRQDVTVLGSQEGAMHSALAGATEVDSGDGNHMFAGHLKCKVRMEKLRIKGMSYTM
CPGKFSIDKEMAETQHGTTVVKVKYEGAGAPCKVPIEIRDVNKEKVVGRIISSTPLAENTNSATNIELEPPFGDSYIVIGVGN
SALTLHWFRKGSSIGKMFESTYRGAKRMAILGETAWDFGSVGGLLTSLGKAVHQVFGSVYTTMFGGVSWMIRILIGFLVLWIG
TNSRNTSMAMTCIAVGGITLFLGFTVQADSGCVVSWKNKELKCGSGIFITDNVHTWTEQYKFQPESPSKLASAIQKAHEEDIC
GIRSVTRLENLMWKQITPELNHILSENEVKLTIMTGDIKGIMQAGKRSLRPQPTELKYSWKTWGKAKMLSTESHNQTFLIDGP
ETAECPNTNRAWNSLEVEDYGFGVFTTNIWLKLKEKQDVFCDSKLMSAAIKDNRAVHADMGYWIESALNDTWKIEKASFIEVK
NCHWPKSHTLWSNGVLESEMIIPKNLAGPVSQHNYRPGYHTQITGPWHLGKLEMDFDFCDGTTVVVTEDCGNRGPSLRTTTAS

GKLITEWCCRSCTLPPLRYRGEDGCWYGMEIRPLKEKEENLVNSLVTAGHGQVDNFSLGVLGMALFLEEMLRTRVGTKHAILL
VAVSFVTLITGNMSFRDLGRVMVMVGATMTDDIGMGVTYLALLAAFKVRPTFAAGLLLRKLTSKELMMTTIGIVLLSQSTIPE
TILELTDALALGMMVLKMVRNMEKYQLAVTIMAILCVPNAVILQNAWKVSCTILAVVSVSPLFLTSSQQKTDWIPLALTIKGL
NPTAIFLTTLSRTSKKRSWPLNEAIMAVGMVSILASSLLKNDIPMTGPLVAGGLLTVCYVLTGRSADLELERAADVKWEDQAE
ISGSSPILSITISEDGSMSIKNEEEEQTLTILIRTGLLVISGLFPVSIPITAAAWYLWEVKKQRAGVLWDVPSPPPMGKAELE
DGAYRIKQKGILGYSQIGAGVYKEGTFHTMWHVTRGAVLMHKGKRIEPSWADVKKDLISYGGGWKLEGEWKEGEEVQVLALEP
GKNPRAVQTKPGLFKTNAGTIGAVSLDFSPGTSGSPIIDKKGKVVGLYGNGVVTRSGAYVSAIAQTEKSIEDNPEIEDDIFRK
RRLTIMDLHPGAGKTKRYLPAIVREAIKRGLRTLILAPTRVVAAEMEEALRGLPIRYQTPAIRAVHTGREIVDLMCHATFTMR
LLSPVRVPNYNLIIMDEAHFTDPASIAARGYISTRVEMGEAAGIFMTATPPGSRDPFPQSNAPIIDEEREIPERSWNSGHEWV
TDFKGKTVWFVPSIKAGNDIAACLRKNGKKVIQLSRKTFDSEYVKTRTNDWDFVVTTDISEMGANFKAERVIDPRRCMKPVIL
TDGEERVILAGPMPVTHSSAAQRRGRIGRNPKNENDQYIYMGEPLENDEDCAHWKEAKMLLDNINTPEGIIPSMFEPEREKVD
AIDGEYRLRGEARKTFVDLMRRGDLPVWLAYRVAAEGINYADRRWCFDGVKNNQILEENVEVEIWTKEGERKKLKPRWLDARI
YSDPLALKEFKEFAAGRKSLTLNLITEMGRLPTFMTQKARDALDNLAVLHTAEAGGRAYNHALSELPETLETLLLLTLLATVT
GGIFLFLMSARGIGKMTLGMCCIITASILLWYAQIQPHWIAASIILEFFLIVLLIPEPEKQRTPQDNQLTYVVIAILTVVAAT
MANEMGFLEKTKKDLGLGSIATQQPESNILDIDLRPASAWTLYAVATTFVTPMLRHSIENSSVNVSLTAIANQATVLMGLGKG
WPLSKMDIGVPLLAIGCYSQVNPITLTAALFLLVAHYAIIGPGLQAKATREAQKRAAAGIMKNPTVDGITVIDLDPIPYDPKF
EKQLGQVMLLVLCVTQVLMMRTTWALCEALTLATGPISTLWEGNPGRFWNTTIAVSMANIFRGSYLAGAGLLFSIMKNTTNTR
RGTGNIGETLGEKWKSRLNALGKSEFQIYKKSGIQEVDRTLAKEGIKRGETDHHAVSRGSAKLRWFVERNMVTPEGKVVDLGC
GRGGWSYYCGGLKNVREVKGLTKGGPGHEEPIPMSTYGWNLVRLQSGVDVFFIPPEKCDTLLCDIGESSPNPTVEAGRTLRVL
NLVENWLNNNTQFCIKVLNPYMPSVIEKMEALQRKYGGALVRNPLSRNSTHEMYWVSNASGNIVSSVNMISRMLINRFTMRYK
KATYEPDVDLGSGTRNIGIESEIPNLDIIGKRIEKIKQEHETSWHYDQDHPYKTWAYHGSYETKQTGSASSMVNGVVRLLTKP
WDVVPMVTQMAMTDTTPFGQQRVFKEKVDTRTQEPKEGTKKLMKITAEWLWKELGKKKTPRMCTREEFTRKVRSNAALGAIFT
DENKWKSAREAVEDSRFWELVDKERNLHLEGKCETCVYNMMGKREKKLGEFGKAKGSRAIWYMWLGARFLEFEALGFLNEDHW
FSRENSLSGVEGEGLHKLGYILRDVSKKEGGAMYADDTAGWDTRITLEDLKNEEMVTNHMEGEHKKLAEAIFKLTYQNKVVRV
QRPTPRGTVMDIISRRDQRGSGQVGTYGLNTFTNMEAQLIRQMEGEGVFKSIQHLTITEEIAVQNWLARVGRERLSRMAISGD
DCVVKPLDDRFASALTALNDMGKIRKDIQQWEPSRGWNDWTQVPFCSHHFHELIMKDGRVLVVPCRNQDELIGRARISQGAGW
SLRETACLGKSYAQMWSLMYFHRRDLRLAANAICSAVPSHWVPTSRTTWSIHAKHEWMTTEDMLTVWNRVWIQENPWMEDKTP
VESWEEIPYLGKREDQWCGSLIGLTSRATWAKNIQAAINQVRSLIGNEEYTDYMPSMKRFRREEEEAGVLW
