# Supplementary material for: Comparison of Neutralizing Dengue Virus B Cell Epitopes and Protective T Cell Epitopes With Those in Three Main Dengue Virus Vaccines
Source: Front Immunol. 2021 Aug 20;12:715136. doi: 10.3389/fimmu.2021.715136 (PMC8417696; doi:10.3389/fimmu.2021.715136)
Supplement: Supplementary file 2 [file DataSheet_2.docx]

**A method to calculate the score resulting of multiple epitopes with different conservation percentages**

Conservancy is defined as the fraction of protein sequences that contain the epitope. It can be calculated in two ways: linearly or discontinuously [1].

The vaccines studied here are constituted of multiple epitopes, each one presenting a different fraction of conservation through the four DENV serotypes as described in the main text. How to give scores to vaccine formulations based on conservation of their epitopes?

We have devised a method to calculate the score based on the following assumptions:

1. Greater conservation for epitopes contributes significantly to total immune activation levels [2].
2. *In vitro* experiments for HIV have shown that point mutations in epitopes are able to cause loss of neutralizing capabilities of antibodies [3].
3. Also, for HIV, match or near-match between the epitope induced by vaccination and the infecting viral strain must occur in order to have an effective vaccine [4]. This means that a near-match can also be effective. The authors claim that this might be generic for other viruses.
4. A simple procedure to describe saturation mutagenesis is well described by Derbyshire [5]. The prediction has a bell shape (probabilistic approach), which can be better approximated by an exponential function than a linear one, where high probabilities of epitope binding to antibodies or HLA molecules are reached when the coverage is 100%, decreasing to zero as mutations are inputted.
5. The main objective in this issue was to compare the levels of conservation of epitopes among vaccines. So an approximate function is enough. Given that the likelihood to elicit the immune response decreases as amino acid replacements take place in epitopes, an exponential function is better than a linear one and it also solves conflict like: “which is better, two epitopes with 50% conservation or only one with 100% conservation?”.

In order to define a simple function score, we set two points (0,100) and fit an exponential function:

| 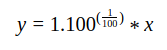 | **(Equation 1)** |
| --- | --- |

**References**

[1] Bui H. H,Sidney J, Li W, Fusseder N, Sette A., 2007. Development of an epitope conservancy analysis tool to facilitate the design of epitope-based diagnostics and vaccines. BMC Bioinformatics 8(1):361.

[2] Derbyshire, Keith M., Joseph J. Salvo, and Nigel DF Grindley. "A simple and efficient procedure for saturation mutagenesis using mixed oligodeoxynucleotides."*Gene*46.2-3 (1986): 145-152.

[3] Westernberg, Luise, et al. "T-cell epitope conservation across allergen species is a major determinant of immunogenicity."*Journal of Allergy and Clinical Immunology*138.2 (2016): 571-578.

[4] Li, Fusheng, et al. "Mapping HIV-1 vaccine induced T-cell responses: bias towards less-conserved regions and potential impact on vaccine efficacy in the Step study."*PloS one*6.6 (2011): e20479.

[5] Derbyshire, Keith M., Joseph J. Salvo, and Nigel DF Grindley. "A simple and efficient procedure for saturation mutagenesis using mixed oligodeoxynucleotides."*Gene*46.2-3 (1986): 145-152.
