# Supplementary material for: Comparison of Neutralizing Dengue Virus B Cell Epitopes and Protective T Cell Epitopes With Those in Three Main Dengue Virus Vaccines
Source: Front Immunol. 2021 Aug 20;12:715136. doi: 10.3389/fimmu.2021.715136 (PMC8417696; doi:10.3389/fimmu.2021.715136)
Supplement: Supplementary file 3 [file DataSheet_3.docx]

Supplementary table 1. Survey of polyprotein sequences of circulating DENV1-4 viruses.

| **World Regions** | **Access number / Country** | | | |
| --- | --- | --- | --- | --- |
|  | **DENV1** | **DENV2** | **DENV3** | **DENV4** |
|  |  |  |  |  |
| **The Americas** | AFJ91714_USA | AEH59348_USA | AAM51538_Martinique | ACS32013_Puerto_Rico |
|  | ALJ53459_USA | AEH59347_USA | AHG23270_Granada | ACS32019_Puerto_Rico |
|  | ACF49259_USA | AEH59346_USA | ACQ44481_Mexico | ALJ53458_Haiti |
|  | AHI43752_Mexico | AEH59342_USA | ACQ44480_Mexico | AEW50183_Brazil |
|  | AHI43751_Mexico | AET72454_USA | ACQ44479_Mexico | ANK35835_Brazil |
|  | AHG23208_Mexico | AHI43753_Mexico | AMH87207_Cuba | ACW83012_Colombia |
|  | AHG23209_Mexico | AHI43694_Mexico | AHG23233_Puerto_Rico | ACW83011_Colombia |
|  | AHG23193_Mexico | AHI43693_Mexico | AHG23230_Puerto_Rico | AET43237_Venezuela |
|  | AHI43732_Mexico | AHI43692_Mexico | AHG23221_Puerto_Rico | ACW82933_Venezuela |
|  | AHI43729_Mexico | AHI43691_Mexico | AHC98458_Nicaragua |  |
|  | ABG75766_Hawaii | AAG30730_Martinique | AHC98457_Nicaragua |  |
|  | ARQ80406_Saint_Barthelemy | AIK23226_Cuba | ABV03585_Brazil |  |
|  | AHI43749_Puerto_Rico | ACK57817_Guadeloupe | AFK83762_Brazil |  |
|  | AHI43750_Puerto_Rico | AHG23133_Puerto_Rico | AHX22016_Peru |  |
|  | AHI43748_Puerto_Rico | AER45462_Guatemala | AHI43684_Peru |  |
|  | AHI43747_Puerto_Rico | AGX15388_Peru | AHG23252_Venezuela |  |
|  | AHC98446_Nicaragua | AHG23138_Nicaragua | ACW82877_Venezuela |  |
|  | AHC98445_Nicaragua | AHG23135_Nicaragua | AFK83764_Paraguay |  |
|  | AHC98444_Nicaragua | ACQ44488_Honduras | AFK83763_Paraguay |  |
|  | AHC98443_Nicaragua | APW84878_Haiti | ADA60766_Colombia |  |
|  | AET43248_El_Salvador | AOE23002_Haiti | ACQ44496_Ecuador |  |
|  | ALJ53459_Haiti | BAD36760_Dominican_Republic |  |  |
|  | ATJ00092_Ecuador | BAD36759_Dominican_Republic |  |  |
|  | AGN94879_Brazil | AGN94892_Brazil |  |  |
|  | AGN94878_Brazil | AGN94891_Brazil |  |  |
|  | AGN94877_Brazil | AGN94890_Brazil |  |  |
|  | AET43246_Venezuela | AGN94889_Brazil |  |  |
|  | AET43256_Venezuela | AET43238_Venezuela |  |  |
|  | AEA50936_Venezuela | ACH61726_Colombia |  |  |
|  | AHI43687_Colombia | ACH61724_Colombia |  |  |
|  | AHI43686_Colombia | AET43238_Venezuela |  |  |
|  | AHF50492_Argentina |  |  |  |
|  | AHF50491_Argentina |  |  |  |
|  | AHF50491_Argentina |  |  |  |
|  | ABO38807_French_Guiana |  |  |  |
|  |  |  |  |  |
| **Europe** | AIN75463_Germany |  |  |  |
|  |  |  |  |  |
| **Asia** | AGT63075_Laos | ARO84705_Malaysia | ALS05358_Philippines | AHG23274_Cambodia |
|  | AGT63074_Laos | ARO84704_Malaysia | AIH13925_Saudi_Arabia | ACW82884_Philippines |
|  | AIE17470_Sri_Lanka | AHG25313_Philippines | AHC72431_Pakistan | AEX09561_India |
|  | AKC32653_Sri_Lanka | AHG23170_Cambodia | AAX19004_Thailand | AHN50410_Sri_Lanka |
|  | AHG23212_Vietnam | AHG23169_Cambodia | AAW66608_Thailand | AGE13482_Singapore |
|  | ACO06148_Thailand | AII99332_India | ASN77913_China | BAU45389_Japan |
|  | AMN88556_Brunei | BAX09288_Thailand | ALJ02589_China | ALI16138_South_Korea |
|  | AET43254_Cambodia | ACS32039_Sri_Lanka | AFI55000_Sri_Lanka | ALL54587_Thailand |
|  | ARO84721_Malaysia | ACS32038_Sri_Lanka | AAM51537_Sri_Lanka | AKQ62916_China |
|  | AUA17938_Taiwan | AHB51029_China | AAT69740_Indonesia | AHG06383_Indonesia |
|  | AHL19966_China | ASN77915_Myanmar | AHG23242_India | AHC72432_Pakistan |
|  | ARM59244_Singapore | AIE17400_Pakistan | AHG23225_Vietnam | AAW30973_Taiwan |
|  | BAQ08293_Japan | AAF18447_China | SNQ41864_Malaysia | ABR13879_Malaysia |
|  | AJP08954_India | ARM59246_Singapore | ARM59248_Singapore |  |
|  | BAU61357_Philippines | AAW51407_Indonesia | BAE48725_East_Timor |  |
|  | ALI16134_South_Korea | AIH13924_Saudi_Arabia | AHG23229_Cambodia |  |
|  | AIG59667_Saudi_Arabia | AHZ61501_Taiwan | AAW23129_Taiwan |  |
|  | BAD42414_Indonesia | ABW06614_Brunei | AUZ41826_Laos |  |
|  | AAW28114_Myanmar | AGO67249_Vietnam | ACL98978_Vietnam |  |
|  |  |  | ABD65876_Bangladesh |  |
|  |  |  |  |  |
| **Africa** | AAK60418_Djibouti | ATO98049_Tanzania | ACQ44384_Mozambique |  |
|  | ABC07334_Comoros | AQV12084_Burkina_Faso |  |  |
|  |  | BAU45374_East_Africa |  |  |
|  |  | ABR13877_Senegal |  |  |
|  |  |  |  |  |
| **Oceania** | AMN88557_Australia | AAK67712_Australia | AFN80339_Australia | AFY10039_South_Pacific_Islands |
|  | AFY10029_New_Caledonia | ACQ44517_Papua_New_Guinea | AFN80338_Australia | AFY10035_New_Caledonia |
|  | AAB70696_Nauru_Island | ACJ04201_Papua_New_Guinea | AFY10053_South_Pacific_Island | AFY10030_French_Polynesia |
|  | ACI48993_Chile_Easter_Island | AAV70829_Tonga | AAW66477_French_Polynesia |  |
|  |  | ADK26435_Guam |  |  |

Supplementary table 2. B cell epitopes, recovered from the IEDB, with positive neutralization tests.

| **Validation source** | **IEDB ID** | **Epitope strings** |
| --- | --- | --- |
|  |  |  |
| **DENV1** | 167478 | K73, G300 |
|  | 173906 | T331, N332, Q411, Y412, L415, K416, G439, T440, T445, P446, Q447, E452, I453, L455, G554, T555, L588, K590, E664, K665 |
|  | 224587 | L46, K47, E49, T51, N52, K136, S138, S155, T156, E157, T160, T161, A162, T163, T165, T170, T171, E172, I173, Q174, T176, D177, S273, G274, T275, T276 |
|  | 240945 | E49, K64, Q77, W101, L107, V122, N134, I161, A162, P169, T200, K202, K295, L308, E309, Q323, W391, F392 |
|  | 240946 | E49, K64, Q77, W101, N134, I161, A162, P169, T200, K202, E203, K310, Q323, W391, F392 |
|  | 240947 | E49, K64, Q77, W101, V122, N134, I161, A162, P169, T200, K202, E203, K310, Q323, W391, F392 |
|  | 240948 | E49, K64, Q77, W101, V122, N153, T155, I161, A162, P169, T200, K202, K202, E203, K310, Q323, W391, F392 |
|  | 240949 | E49, K64, Q77, W101, V122, N153, T155, I161, A162, P169, T200, K202, K310, Q323, W391, F392 |
|  | 240950 | E49, Q77, L107, N134, I161, A162, Q167, P169, Q174, E203 |
|  | 240951 | E49, Q77, W101, N134, I161, A162, P169, T200, E203, Q323, W391, F392 |
|  | 240952 | E49, Q77, W101, V122, N153, T155, I161, A162, P169, T200, K202, Q323, W391, F392 |
|  | 240953 | W101 |
|  | 240954 | W101, L107 |
|  | 504078 | W101, L107, G111 |
|  | 504083 | R73, G78, E79 |
|  | 504136 | F586, K587, L588, E589, K590, E591, V592, A593, E594, T595, G598, T599, V600, L601, V602, Q603, V604, K605, E642, I647, E648, S670, F672, K674, G675, S676, S677, I678, G679, K680 |
|  | 591574 | L46, K47, E49, T51, N52, K136, S138, S155, T156, E157, T160, T161, A162, T163, T165, T170, T171, E172, I173, Q174, T176, D177, S273, G274, T275, T276 |
|  | 745514 | E49, K64, Q77, W101, V122, N153, T155, I161, A162, P169, T200, K202, K310, Q323, W391, F392 |
|  | 745515 | E49, Q77, W101, N134, I161, A162, P169, T200, E203, Q323, W391, F392 |
|  | 224587 | L46, K47, E49, T51, N52, K136, S138, S155, T156, E157, T160, T161, A162, T163, T165, T170, T171, E172, I173, Q174, T176, D177, S273, G274, T275, T276 |
|  |  |  |
| **DENV2** | 167479 | R228 |
|  | 240770 | A: H438, S554, V589, K590, E591 |
|  | 240773 | A: T350, E351, S352, C354, Q357, S361, L362, N363, E364, R379, W381, G382, N383, G384, C385, G386, I393, T395, K526, K527, Q528 |
|  | 433721 | A: N347, T348, T349, T350, A351, S352, R353, C354, W381, G382, N383, G384, I393, K527 |
|  | 504074 | N103, G104, G111 |
|  | 504134 | F586, K587, V588, V589, K590, E591, I592, A593, E594, T595, H597, T599, I600, V601, R603, Q605, V645, N646, I647, E648, S676, S677 |
|  | 540687 | K305, K307, K310 |
|  | 540688 | K305, K310, E311 |
|  | 540689 | T303, G304, K307 |
|  | 540690 | V382, P384 |
|  | 753469 | K307, V309, K310, Q316, G318, D362, S363, P364 |
|  | 753470 | K307, V309, Q316, D362, P364 |
|  | 753471 | K160, E161, I162, K163, I170, T171, A173, E174, T176, G177, T180 |
|  |  |  |
| **DENV3** | 178101 | W101, G109 |
|  | 178102 | W101, L107, G109 |
|  | 196270 | K140 |
|  | 196271 | L117, S119, E123, K140 |
|  | 241577 | A50, T51, Q52, L53, A54, T55, R73, C74, W101, G106, E126, K128, V130, Q131, E133, N134, Q148, L196, T198, T274, I276, K307, K308, E309 |
|  | 489869 | F115, K140 |
|  | 489872 | L117, S119 |
|  | 489874 | S119, G216 |
|  | 504071 | W101 |
|  | 504072 | W101, G106 |
|  | 504073 | W101, G111 |
|  | 504117 | V585, L586, K587, K588, E589, V590, S591, E592, G596, T597, I598, L599, I600, K601, V602, E603, V643, N644, I645, I667, W669, S674 |
|  | 538524 | Q52, L53, E126, K128, E133, L135, A203 |
|  |  |  |
| **DENV4** | 196291 | K139 |
|  | 504135 | F585, S586, I587, D588, K589, E590, M591, A592, E593, T594, T599, V600, V601, K602, V603, K604, E606, N641, V643, T644, N645, I646, L668, H669, W670, G678, K679 |
|  | 591353 | K330, K403, K479, K481 |
|  | 591354 | K330, V332, K403, L414, K479, K513 |

Supplementary table 3. T cell epitopes with protective correlates selected from the articles.

| **Reference** | **ID** ^a^ | **Epitopes** | **Location** | **HLA allele** | **Characterization source** |
| --- | --- | --- | --- | --- | --- |
| **(WEISKOPF et al., 2015)** |  |  |  |  |  |
|  | 001 | FNMLKRARNR | C 14-23 | A*3301 | DENV1 |
|  | 002 | MLKRERNRV | C 16-24 | B*0801 | DENV1 |
|  | 003 | KEISSMLNIM | C 87-96 | B*4001 | DENV1 |
|  | 004 | TEVTNPAVL | E 329-337 | B*4001 | DENV1 |
|  | 005 | PTSEIQLTDY | E 450-459 | A*0101 | DENV1 |
|  | 006 | LTDYGALTL | E 456-464 | A*0101 | DENV1 |
|  | 007 | RPGYHTQTA | NS1 1033-1041 | B*0702 | DENV1 |
|  | 008 | FRRLTSREVL | NS2A 1221-1230 | B*0801 | DENV1 |
|  | 009 | LTDFQSHQL | NS2A 1265-1273 | A*0101 | DENV1 |
|  | 010 | GPLVAGGLL | NS4B 1377-1385 | B*0702 | DENV1 |
|  | 011 | IYRILQRGLL | NS3 1499-1508 | B*0801 | DENV1 |
|  | 012 | NPEIEDDIF | NS3 1653-1661 | B*3501 | DENV1 |
|  | 013 | AIKRKLRTL | NS3 1689-1697 | B*0801 | DENV1 |
|  | 014 | TPEGIIPSM | NS3 1978-1986 | B*0702, B*3501 | DENV1 |
|  | 015 | RPRWLDART | NS3 2070-2078 | B*0702 | DENV1 |
|  | 016 | AGRRSVSGDL | NS3 2093-2102 | B*0801 | DENV1 |
|  | 017 | NPLTLTAAV | NS4B 2350-2358 | B*0702 | DENV1 |
|  | 018 | SPGKFWNTTI | NS4B 2455-2464 | B*0702 | DENV1 |
|  | 019 | IPMVTQIAM | NS5 2830-2838 | B*0702 | DENV1 |
|  | 020 | VEDERFWDL | NS5 2921-2929 | B*4001 | DENV1 |
|  | 021 | TVMDVISRR | NS5 3083-3091 | A*6801 | DENV1 |
|  | 022 | REIVVPCRNQ | NS5 3218-3227 | B*4001 | DENV1 |
|  | 023 | TWSIHAHHQW | NS5 3290-3299 | A*2301 | DENV1 |
|  | 024 | GPMKLVMAFI | C 43-52 | B*0702 | DENV1, 3 |
|  | 025 | GPWHLGKLEL | NS1 1042-1051 | B*0702 | DENV1, 3 |
|  | 026 | KPGTSGSPI | NS3 1608-1616 | B*0702 | DENV1, 3 |
|  | 027 | RVIDPRRCL | NS3 1899-1907 | B*0702 | DENV1, 3 |
|  | 028 | DPRRCLKPV | NS3 1902-1910 | B*0702 | DENV1, 3 |
|  | 029 | TPEGIIPALF | NS3 1978-1987 | B*0702, B*3501, B*5301 | DENV1, 3 |
|  | 030 | EAKQPATLR | E 330-338 | A*3301, A*6801 | DENV2 |
|  | 031 | ILIGVIITW | E 738-746 | B*5701, B*5801 | DENV2 |
|  | 032 | VTYECPLLV | M 164-172 | A*0201 | DENV2 |
|  | 033 | IQKETLVTF | E 513-521 | A*2301, B*1501 | DENV2 |
|  | 034 | IQMSSGNLLF | E 551-560 | A*2301 | DENV2 |
|  | 035 | SYSMCTGKF | E 579-587 | A*2301 | DENV2 |
|  | 036 | GAAFSGVSW | E 726-734 | B*5801 | DENV2 |
|  | 037 | HPGFTILALF | M 245-254 | B*3501 | DENV3 |
|  | 038 | MLVTPSMTM | M 274-282 | B*3501 | DENV3 |
|  | 039 | LPEYGTLGLE | E 456-465 | B*3501 | DENV3 |
|  | 040 | LPWTSGATT | E 499-507 | B*3501 | DENV3 |
|  | 041 | MSYAMCTNTF | E 578-587 | B*3501 | DENV3 |
|  | 042 | RSCTLPPLRY | NS1 1090-1099 | A*0101 | DENV3 |
|  | 043 | RPINEKEENM | NS1 1112-1121 | B*3501 | DENV3 |
|  | 044 | LAILFEEVM | NS2A 1143-1151 | B*3501 | DENV3 |
|  | 045 | MIAGVFFTF | NS2A 1160-1168 | B*3501 | DENV3 |
|  | 046 | DPASIAARGY | NS3 1768-1777 | B*3501 | DENV3 |
|  | 047 | MEGVFHTMW | NS3 1519-1527 | B*4403 | DENV4 |
|  | 048 | GEIGAVTLDF | NS3 1598-1607 | B*4001, B*4403 | DENV4 |
|  | 049 | LEENMEVEIW | NS3 2051-2060 | B*4403, B*4402 | DENV4 |
|  | 050 | GERKKLKPRW | NS3 2064-2073 | B*4403 | DENV4 |
|  | 051 | IAVASGLLW | NS4A 2184-2192 | B*5701 | DENV4 |
|  | 052 | RSNAAIGAVF | NS5 2899-2908 | B*1501 | DENV4 |
|  | 053 | YAQMWSLMY | NS5 3254-3262 | B*1501, B*5301 | DENV4 |
|  | 054 | MVLALITFL | C 45-53 | A*0201 | DENV4 |
|  | 055 | NYKERMVTF | E 513-521 | A*2402 | DENV4 |
|  | 056 | PPASDLKYSW | NS1 880-890 | B*5301 | DENV4 |
|  | 057 | IEKASLIEV | NS1 988-996 | B*4001 | DENV4 |
|  | 058 | IPHDLMELI | NS2A 1244-1252 | B*5301 | DENV4 |
|  | 059 | YPLAIPVTM | NS2B 1457-1465 | B*5301 | DENV4 |
|  | 060 | YSDPLALRE | NS3 2079-2087 | A*0101 | DENV4 |
|  | 061 | LGKSYAQMW | NS5 3250-3258 | B*5701 | DENV4 |
|  | 062 | ITLLCLIPTV | C 104-113 | A*0201 | DENV4 |
|  | 063 | ALPVYLMTL | NS2A 1332-1340 | A*0201 | DENV4 |
|  | 064 | EETNMITLL | NS2B 1437-1445 | B*4001, B*4403 | DENV4 |
|  | 065 | ALSEGVYRI | NS3 1494-1502 | A*0201 | DENV4 |
|  | 066 | TPRMCTREEF | NS5 2885-2894 | B*0702, B*5301 | DENV1-4 |
|  | 067 | GLYGNGVVTK | NS3 1625-1634 | A*0301 | DENV1-4 |
|  | 068 | TTWEDVPYL | NS5 3327-3338 | A*0201 | DENV1-4 |
|  | 069 | IAVSMANIF | NS4B 2464-2472 | B*3501 | DENV1-4 |
|  | 070 | KVRKDIPQW | NS5 3182-3190 | B*5701, B*5801 | DENV1-4 |
|  | 071 | GLFGKTQVGV | NS3 1506-1515 | A*0201 | DENV1-4 |
|  | 072 | ETACLGKSY | NS5 3246-3254 | A*2601 | DENV1-4 |
|  | 073 | HTWTEQYKF | NS1 802-810 | B*5701 | DENV1-4 |
|  | 074 | LPVYLMTLMK | NS2A 1333-1342 | B*3501 | DENV1-4 |
|  | 075 | ALWYVWQVK | NS2B 14631472 | A*0301 | DENV1-4 |
|  | 076 | VLDDGIYRI | NS3 1494-1502 | A*0201 | DENV1-4 |
|  | 077 | GTSGSPIINK | NS3 160-1617 | A*0301, A*1101 | DENV1-4 |
|  | 078 | APTRVVAAEM | NS3 1700-1709 | B*0702, B*3501 | DENV1-4 |
|  | 079 | VPNYNLIVM | NS3 1753-1761 | B*3501 | DENV1-4 |
|  | 080 | EERDIPERSW | NS3 1813-1822 | B*4402 | DENV1-4 |
|  | 081 | DISEMGANF | NS3 1887-1895 | A*2601 | DENV1-4 |
|  | 082 | RVIDPRRCLK | NS3 1899-1908 | A*0301 | DENV1-4 |
|  | 083 | EFRLRGEQR | NS3 2000-2008 | A*3301 | DENV1-4 |
|  | 084 | MANIFRGSY | NS4B 2468-2476 | B*3501 | DENV1-4 |
|  | 085 | IMKSVGTGK | NS4B 2486-2494 | A*0301 | DENV1-4 |
|  | 086 | ATYGWNLVK | NS5 2612-2620 | A*0301 | DENV1-4 |
|  | 087 | ASSMVNGVVR | NS5 2812-2821 | B*5701 | DENV1-4 |
|  | 088 | TPFGQQRVF | NS5 2842-2850 | B*3501 | DENV1-4 |
|  | 089 | KLGEFGRAK | NS5 2956-2964 | A*0301 | DENV1-4 |
|  | 090 | KAKGSRAIW | NS5 2962-2970 | B*5701 | DENV1-4 |
|  | 091 | RFLEFEALGF | NS5 2977-2986 | A*2301 | DENV1-4 |
|  | 092 | ALLALNDMGK | NS5 3173-3182 | A*0301 | DENV1-4 |
|  | 093 | WSIHAHHQW | NS5 3291-3299 | B*5701 | DENV1-4 |
|  | 094 | MVHQIFGSAY | E 716-725 | B*1501, B*3501 | DENV1-4 |
| **(YAUCH et al., 2009)** |  |  |  |  |  |
|  | 095 | VAFLRFLTI | C 51-59 | DR5 | DENV2 |
|  | 096 | FSLGVLGM | NS2A 8-15 | DR5 | DENV2 |
|  | 097 | YSQVNPITL | NS4B 99-107 | DR5 | DENV2 |
|  | 098 | RMLINRFTM | NS5 237-245 | DR5 | DENV2 |
| **(YAUCH et al., 2010)** |  |  |  |  |  |
|  | 099 | GLFPVSLPITAAAWY | NS2B 108-122 | DR5 | DENV2 |
|  | 100 | GKTKRYLPAIVREAI | NS3 198-212 | DR5 | DENV2 |
|  | 101 | GLPIRYQTPAIRAEH | NS3 237-51 | DR5 | DENV2 |
| **(GRIFONI et al., 2017)** |  |  |  |  |  |
|  | 102 | GPMKLVMAFI | C 43–52 | B * 0702 | DENV2 |
|  | 103 | MSSGNLLFTG | E 553–562 | B * 5801 | DENV2 |
|  | 104 | RLITVNPIV | E 631–639 | A * 0201/0203 | DENV2 |
|  | 105 | AIYGAAFSGV | E 723–732 | A * 0201/0203/0206 | DENV2 |
|  | 106 | ILIGVIITW | E 738–746 | B * 5801 | DENV2 |
|  | 107 | ILIGVVITW | E 738–746 | B * 5801 | DENV2 |
|  | 108 | RAVHADMGY | NS1 968–976 | B * 5801 | DENV2 |
|  | 109 | GPWHLGKLEL | NS1 1042-1051 | B * 0702 | DENV2 |
|  | 110 | FLEEMLRTRV | NS2A 1146–1155 | A * 0201 | DENV2 |
|  | 111 | ILLVAVSFV | NS2A 1161–1169 | A * 0201 | DENV2 |
|  | 112 | TMTDDIGMGV | NS2A 1191-1200 | A * 0201 | DENV2 |
|  | 113 | MMATIGIALL | NS2A 1230-1239 | A * 0201 | DENV2 |
|  | 114 | LSIPHDLMEF | NS2A 1242–1251 | A * 0101 | DENV2 |
|  | 115 | LIDGISLGL | NS2A 1251-1259 | A * 0101 | DENV2 |
|  | 116 | YQLAVTIMA | NS2A 1271-1279 | A * 0201/0206 | DENV2 |
|  | 117 | TAAAWYLWEV | NS2B 1464–1473 | A * 0201/0206/6802 | DENV2 |
|  | 118 | SEMGANFKA | NS3 1889–1897 | B * 4403 | DENV2 |
|  | 119 | DPRRCLKPV | NS3 1902-1910 | B * 0702 | DENV2 |
|  | 120 | TPEGIIPALF | NS3 1978–1987 | B * 0702/3501/5301 | DENV2 |
|  | 121 | RPRWLDART | NS3 2070–2078 | B * 0702 | DENV2 |
|  | 122 | LEFFLMVLLI | NS4A 2207–2216 | B * 4403 | DENV2 |
|  | 123 | YVVIAILTVV | NS4B 2232–2241 | A * 0201/6802 | DENV2 |
|  | 124 | RPASAWTLY | NS4B 2280–2288 | B * 0702 | DENV2 |
|  | 125 | TLYAVATTFV | NS4B 2286–2295 | A * 0201 | DENV2 |
|  | 126 | LMGLGKGWPL | NS4B 2322–2331 | A * 0201 | DENV2 |
|  | 127 | LLLVAHYAI | NS4B 2359–2367 | A * 0201 | DENV2 |
|  | 128 | MLLILCVTQV | NS4B 2419–2428 | A * 0201 | DENV2 |
|  | 129 | LLILCVTQV | NS4B 2420–2428 | A * 0201 | DENV2 |
|  | 130 | VLNPYMPSV | NS5 2677–2685 | A * 0201 | DENV2 |
|  | 131 | IPMSTYGWNL | NS5 2609–2618 | B * 0702 | DENV2 |
|  | 132 | IPMATYGWNL | NS5 2609–2618 | B * 0702 | DENV2 |
|  | 133 | EAVEDGRFWE | NS5 2919–2928 | B * 5801 | DENV2 |
|  | 134 | VEDEDFWKL | NS5 2921–2929 | B * 4001 | DENV2 |
|  | 135 | KLAEAIFKL | NS5 3058-3066 | A * 0201 | DENV2 |
|  | 136 | MEVQLVRQM | NS5 3110-3118 | B * 4001 | DENV2 |
|  | 137 | QQVPFCSHHF | NS5 3200-3209 | B * 4403 | DENV2 |
|  | 138 | LRLFMALVAFLRFLT | C 45–59 | DRB1 * 1501 | DENV2 |
|  | 139 | WGTIKKSKAINVLRG | C 70–84 | DRB1 * 0701 | DENV2 |
|  | 140 | WIQKETLVTFKNPHA | E 512–526 | DRB1 * 1501 | DENV2 |
|  | 141 | NRKELLVTFKNAHAK | E 513–527 | DRB1 * 1501 | DENV2 |
|  | 142 | GATEIQMSSGNLLFT | E 547-561 | DRB1 * 0301 | DENV2 |
|  | 143 | ILRHPGFTIMAAILA | M 242–256 | DRB1 * 0701 / DRB1 * 1501 | DENV2 |
|  | 144 | ILRHPGFTLMAAILA | M 242–256 | DRB1 * 1501 | DENV2 |
|  | 145 | FQRVLIFILLTAVAP | M 264–278 | DRB1 * 0701 | DENV2 |
|  | 146 | VKLTIMTGDIKGIMQ | NS1 860-874 | DRB1 * 0301 | DENV2 |
|  | 147 | KLTIMTGDIKGIMQA | NS1 861–875 | DRB1 * 0301 | DENV2 |
|  | 148 | GMGVTYLALLAAYKV | NS2A 1197-1211 | DRB1 * 0101 | DENV2 |
|  | 149 | GVTYLALLAAFKVRP | NS2A 1199–1213 | DRB1 * 0101 | DENV2 |
|  | 150 | LLTIGLSLVASVELP | NS2A 1231–1245 | DRB1 * 0701 | DENV2 |
|  | 151 | LGMMVLKIVRNMEKY | NS2A 1257–1271 | DRB1 * 1202 | DENV2 |
|  | 152 | NTIFTLTVAWRTATL | NS2A 1284-1298 | DRB1 * 0701 | DENV2 |
|  | 153 | IMAVGIVSILLSSLL | NS2B 1355–1369 | DRB1 * 0701 | DENV2 |
|  | 154 | MAVGMVSILASSLLK | NS2B 1356–1370 | DRB1 * 0701 | DENV2 |
|  | 155 | GTFHTMWHVTRGAVL | NS3 1521–1535 | DRB1 * 0701 | DENV2 |
|  | 156 | FHTMWHVTRGAVLTY | NS3 1523–1537 | DRB1 * 0701 | DENV2 |
|  | 157 | FHTMWHVTRGAVLMH | NS3 1523–1537 | DRB1 * 0701 | DENV2 |
|  | 158 | VLMHKGKRIEPSWAD | NS3 1534–1548 | DRB1 * 1301 | DENV2 |
|  | 159 | KGKVVGLYGNGVVTR | NS3 1620–1634 | DRB1 * 1501 | DENV2 |
|  | 160 | GKIVGLYGNGVVTTS | NS3 1621–1635 | DRB1 * 1501 | DENV2 |
|  | 161 | TNCLRKNGKRVIQLS | NS3 1850–1864 | DRB1 * 1301 | DENV2 |
|  | 162 | LGMCCIITASILLWY | NS4A 2179-2193 | DRB1 * 0701 | DENV2 |
|  | 163 | QIMLLILCTSQILLM | NS4B 2417–2431 | DRB1 * 0701 | DENV2 |
|  | 164 | LCAVQLLLMRTSWAL | NS4B 2423–2437 | DRB1 * 1202 | DENV2 |
|  | 165 | RWLWGFLSRNKKPRI | NS5 2874-2888 | DRB1 * 1501 | DENV2 |
| **(ELONG et al., 2016)** |  |  |  |  |  |
|  | 166 | APTRVVASEM | NS3 1700–1709 | B * 0702 | DENV1 |
|  | 167 | LPAIVREAI | NS3 1682–1690 | B * 0702 | DENV1,2,3 |
|  | 168 | RPASAWTLYA | NS4B 2280–2289 | B * 0702 | DENV1,2,4 |
|  | 169 | HPASAWTLYA | NS4B 2280–2289 | B * 0702 | DENV1,3 |
|  | 170 | KPRWLDARI | NS3 2070–2078 | B * 0702 | DENV2 |
|  | 171 | TPRMCTREEF | NS5 2885–2894 | B * 0702 | DENV2 |
|  | 172 | APTRVVAAEM | NS3 1700–1709 | B * 0702 | DENV2,3,4 |
|  | 173 | KPRLCTREEF | NS5 2885–2894 | B * 0702 | DENV3 |
|  | 174 | LPSIVREAL | NS3 1682–1690 | B * 0702 | DENV4 |
|  | 175 | RPKWLDARV | NS3 2070–2078 | B * 0702 | DENV4 |

1. Numbering created by the author to identify the epitope.

Supplementary table 4. Percentage of conservation of B cell epitopes which are target for neutralizing antibodies.

| **Access ID** | **Vaccines** | | | | | | | | | | | | **Circulating viruses** | | | |
| --- | --- | --- | --- | --- | --- | --- | --- | --- | --- | --- | --- | --- | --- | --- | --- | --- |
|  | **Dengvaxia®** | | | | **LAV-TDV** | | | | **TAK-003** | | | |  | | | |
|  | **DENV1** | **DENV2** | **DENV3** | **DENV4** | **DENV1** | **DENV2** | **DENV3** | **DENV4** | **DENV1** | **DENV2** | **DENV3** | **DENV4** | **DENV1** | **DENV2** | **DENV3** | **DENV4** |
| **167478** | 0.00% | 0.00% | 0.00% | 0.00% | 0.00% | 0.00% | 0.00% | 0.00% | 0.00% | 0.00% | 0.00% | 0.00% | 0.00% | 0.00% | 0.00% | 0.00% |
| **173906** | **100.00%** | 0.00% | 0.00% | 0.00% | **100.00%** | 0.00% | 0.00% | 0.00% | **100.00%** | 0.00% | 0.00% | 0.00% | **91.67%** | 0.00% | 0.00% | 0.00% |
| **224587** | 0.00% | 0.00% | 0.00% | 0.00% | 0.00% | 0.00% | 0.00% | 0.00% | 0.00% | 0.00% | 0.00% | 0.00% | **20.00%** | 0.00% | 0.00% | 0.00% |
| **240945** | 0.00% | 0.00% | 0.00% | 0.00% | 0.00% | 0.00% | 0.00% | 0.00% | 0.00% | 0.00% | 0.00% | 0.00% | **63.33%** | 0.00% | 0.00% | 0.00% |
| **240946** | 0.00% | 0.00% | 0.00% | 0.00% | 0.00% | 0.00% | 0.00% | 0.00% | 0.00% | 0.00% | 0.00% | 0.00% | **63.33%** | 0.00% | 0.00% | 0.00% |
| **240947** | 0.00% | 0.00% | 0.00% | 0.00% | 0.00% | 0.00% | 0.00% | 0.00% | 0.00% | 0.00% | 0.00% | 0.00% | **63.33%** | 0.00% | 0.00% | 0.00% |
| **240948** | 0.00% | 0.00% | 0.00% | 0.00% | 0.00% | 0.00% | 0.00% | 0.00% | 0.00% | 0.00% | 0.00% | 0.00% | **63.33%** | 0.00% | 0.00% | 0.00% |
| **240949** | 0.00% | 0.00% | 0.00% | 0.00% | 0.00% | 0.00% | 0.00% | 0.00% | 0.00% | 0.00% | 0.00% | 0.00% | **63.33%** | 0.00% | 0.00% | 0.00% |
| **240950** | 0.00% | 0.00% | 0.00% | 0.00% | 0.00% | 0.00% | 0.00% | 0.00% | 0.00% | 0.00% | 0.00% | 0.00% | **63.33%** | 0.00% | 0.00% | 0.00% |
| **240951** | 0.00% | 0.00% | 0.00% | 0.00% | 0.00% | 0.00% | 0.00% | 0.00% | 0.00% | 0.00% | 0.00% | 0.00% | **63.33%** | 0.00% | 0.00% | 0.00% |
| **240952** | 0.00% | 0.00% | 0.00% | 0.00% | 0.00% | 0.00% | 0.00% | 0.00% | 0.00% | 0.00% | 0.00% | 0.00% | **63.33%** | 0.00% | 0.00% | 0.00% |
| **240953** | 0.00% | 0.00% | 0.00% | 0.00% | 0.00% | 0.00% | 0.00% | 0.00% | 0.00% | 0.00% | 0.00% | 0.00% | 0.00% | 0.00% | 0.00% | 0.00% |
| **240954** | 0.00% | 0.00% | 0.00% | 0.00% | 0.00% | 0.00% | 0.00% | 0.00% | 0.00% | 0.00% | 0.00% | 0.00% | 0.00% | 0.00% | 0.00% | 0.00% |
| **504078** | **100.00%** | **100.00%** | **100.00%** | **100.00%** | **100.00%** | **100.00%** | **100.00%** | **100.00%** | **100.00%** | **100.00%** | **100.00%** | **100.00%** | **100.00%** | **100.00%** | **100.00%** | **100.00%** |
| **504083** | **100.00%** | **100.00%** | **100.00%** | **100.00%** | **100.00%** | **100.00%** | **100.00%** | **100.00%** | **100.00%** | **100.00%** | **100.00%** | **100.00%** | **100.00%** | **100.00%** | **100.00%** | **100.00%** |
| **504136** | 0.00% | 0.00% | 0.00% | 0.00% | **100.00%** | 0.00% | 0.00% | 0.00% | **100.00%** | 0.00% | 0.00% | 0.00% | **55.00%** | 0.00% | 0.00% | 0.00% |
| **591574** | 0.00% | 0.00% | 0.00% | 0.00% | 0.00% | 0.00% | 0.00% | 0.00% | 0.00% | 0.00% | 0.00% | 0.00% | **20.00%** | 0.00% | 0.00% | 0.00% |
| **745514** | 0.00% | 0.00% | 0.00% | 0.00% | 0.00% | 0.00% | 0.00% | 0.00% | 0.00% | 0.00% | 0.00% | 0.00% | **63.33%** | 0.00% | 0.00% | 0.00% |
| **745515** | 0.00% | 0.00% | 0.00% | 0.00% | 0.00% | 0.00% | 0.00% | 0.00% | 0.00% | 0.00% | 0.00% | 0.00% | **63.33%** | 0.00% | 0.00% | 0.00% |
| **224587** | 0.00% | 0.00% | 0.00% | 0.00% | 0.00% | 0.00% | 0.00% | 0.00% | 0.00% | 0.00% | 0.00% | 0.00% | **20.00%** | 0.00% | 0.00% | 0.00% |
| **167479** | 0.00% | 0.00% | 0.00% | 0.00% | 0.00% | 0.00% | 0.00% | 0.00% | 0.00% | 0.00% | 0.00% | 0.00% | 0.00% | 0.00% | 0.00% | 0.00% |
| **240770** | 0.00% | **100.00%** | 0.00% | 0.00% | 0.00% | **100.00%** | 0.00% | 0.00% | 0.00% | **100.00%** | 0.00% | 0.00% | 0.00% | **96.55%** | 0.00% | 0.00% |
| **240773** | 0.00% | **100.00%** | 0.00% | 0.00% | 0.00% | **100.00%** | 0.00% | 0.00% | 0.00% | **100.00%** | 0.00% | 0.00% | 0.00% | **55.17%** | 0.00% | 0.00% |
| **433721** | 0.00% | 0.00% | 0.00% | 0.00% | 0.00% | 0.00% | 0.00% | 0.00% | 0.00% | 0.00% | 0.00% | 0.00% | 0.00% | **25.86%** | 0.00% | 0.00% |
| **504074** | **100.00%** | **100.00%** | **100.00%** | **100.00%** | **100.00%** | **100.00%** | **100.00%** | **100.00%** | **100.00%** | **100.00%** | **100.00%** | **100.00%** | **100.00%** | **100.00%** | **100.00%** | **100.00%** |
| **504134** | 0.00% | **100.00%** | 0.00% | 0.00% | 0.00% | **100.00%** | 0.00% | 0.00% | 0.00% | **100.00%** | 0.00% | 0.00% | 0.00% | **53.45%** | 0.00% | 0.00% |
| **540687** | **100.00%** | **100.00%** | **100.00%** | **100.00%** | 0.00% | **100.00%** | **100.00%** | **100.00%** | **100.00%** | **100.00%** | **100.00%** | **100.00%** | 0.00% | **100.00%** | **100.00%** | **100.00%** |
| **540688** | **100.00%** | **100.00%** | **100.00%** | **100.00%** | 0.00% | **100.00%** | 0.00% | **100.00%** | 0.00% | 100.00% | 0.00% | **100.00%** | **1.67%** | **100.00%** | 0.00% | **100.00%** |
| **540689** | **100.00%** | **100.00%** | 0.00% | 0.00% | **100.00%** | **100.00%** | 0.00% | 0.00% | **100.00%** | **100.00%** | **100.00%** | **100.00%** | **100.00%** | **100.00%** | 0.00% | **32.00%** |
| **540690** | 0.00% | 0.00% | 0.00% | 0.00% | 0.00% | 0.00% | 0.00% | 0.00% | 0.00% | 0.00% | 0.00% | 0.00% | 0.00% | 0.00% | 0.00% | 0.00% |
| **753469** | 0.00% | **100.00%** | 0.00% | 0.00% | 0.00% | **100.00%** | 0.00% | 0.00% | 0.00% | **100.00%** | 0.00% | 0.00% | 0.00% | **98.28%** | 0.00% | 0.00% |
| **753470** | 0.00% | **100.00%** | 0.00% | 0.00% | 0.00% | **100.00%** | 0.00% | 0.00% | 0.00% | **100.00%** | 0.00% | 0.00% | 0.00% | **98.28%** | 0.00% | 0.00% |
| **753471** | 0.00% | **100.00%** | 0.00% | 0.00% | 0.00% | **100.00%** | 0.00% | 0.00% | 0.00% | **100.00%** | 0.00% | 0.00% | 0.00% | **72.41%** | 0.00% | 0.00% |
| **178101** | 0.00% | 0.00% | 0.00% | 0.00% | 0.00% | 0.00% | 0.00% | 0.00% | 0.00% | 0.00% | 0.00% | 0.00% | 0.00% | 0.00% | 0.00% | 0.00% |
| **178102** | **100.00%** | **100.00%** | **100.00%** | **100.00%** | **100.00%** | **100.00%** | **100.00%** | **100.00%** | **100.00%** | **100.00%** | **100.00%** | **100.00%** | **100.00%** | **98.28%** | **100.00%** | **100.00%** |
| **196270** | 0.00% | 0.00% | 0.00% | 0.00% | 0.00% | 0.00% | 0.00% | 0.00% | 0.00% | 0.00% | 0.00% | 0.00% | 0.00% | 0.00% | 0.00% | 0.00% |
| **196271** | 0.00% | 0.00% | **100.00%** | 0.00% | 0.00% | 0.00% | **100.00%** | 0.00% | 0.00% | 0.00% | **100.00%** | 0.00% | 0.00% | 0.00% | **100.00%** | **4.00%** |
| **241577** | 0.00% | 0.00% | **100.00%** | 0.00% | 0.00% | 0.00% | **100.00%** | 0.00% | 0.00% | 0.00% | **100.00%** | 0.00% | 0.00% | 0.00% | **95.65%** | 0.00% |
| **489869** | 0.00% | 0.00% | 0.00% | 0.00% | 0.00% | 0.00% | 0.00% | 0.00% | 0.00% | 0.00% | 0.00% | 0.00% | 0.00% | 0.00% | 0.00% | 0.00% |
| **489872** | 0.00% | 0.00% | 0.00% | 0.00% | 0.00% | 0.00% | 0.00% | 0.00% | 0.00% | 0.00% | 0.00% | 0.00% | 0.00% | 0.00% | 0.00% | 0.00% |
| **489874** | 0.00% | 0.00% | 0.00% | 0.00% | 0.00% | 0.00% | 0.00% | 0.00% | 0.00% | 0.00% | 0.00% | 0.00% | 0.00% | 0.00% | 0.00% | 0.00% |
| **504071** | 0.00% | 0.00% | 0.00% | 0.00% | 0.00% | 0.00% | 0.00% | 0.00% | 0.00% | 0.00% | 0.00% | 0.00% | 0.00% | 0.00% | 0.00% | 0.00% |
| **504072** | 0.00% | 0.00% | 0.00% | 0.00% | 0.00% | 0.00% | 0.00% | 0.00% | 0.00% | 0.00% | 0.00% | 0.00% | 0.00% | 0.00% | 0.00% | 0.00% |
| **504073** | 0.00% | 0.00% | 0.00% | 0.00% | 0.00% | 0.00% | 0.00% | 0.00% | 0.00% | 0.00% | 0.00% | 0.00% | 0.00% | 0.00% | 0.00% | 0.00% |
| **504117** | 0.00% | 0.00% | **100.00%** | 0.00% | 0.00% | 0.00% | **100.00%** | 0.00% | 0.00% | 0.00% | **100.00%** | 0.00% | 0.00% | 0.00% | **97.83%** | 0.00% |
| **538524** | 0.00% | 0.00% | **100.00%** | 0.00% | 0.00% | 0.00% | **100.00%** | 0.00% | 0.00% | 0.00% | **100.00%** | 0.00% | 0.00% | 0.00% | **100.00%** | 0.00% |
| **196291** | 0.00% | 0.00% | 0.00% | 0.00% | 0.00% | 0.00% | 0.00% | 0.00% | 0.00% | 0.00% | 0.00% | 0.00% | 0.00% | 0.00% | 0.00% | 0.00% |
| **504135** | **100.00%** | 0.00% | 0.00% | **100.00%** | 0.00% | 0.00% | 0.00% | **100.00%** | 0.00% | 0.00% | 0.00% | 0.00% | 0.00% | 0.00% | 0.00% | **80.00%** |
| **591353** | **100.00%** | 0.00% | 0.00% | **100.00%** | 0.00% | 0.00% | 0.00% | **100.00%** | 0.00% | 0.00% | 0.00% | **100.00%** | 0.00% | 0.00% | 0.00% | **88.00%** |
| **591354** | **100.00%** | 0.00% | 0.00% | **100.00%** | 0.00% | 0.00% | 0.00% | **100.00%** | 0.00% | 0.00% | 0.00% | **100.00%** | 0.00% | 0.00% | 0.00% | **96.00%** |

Supplementary table 5. Percentage of conservation of T cell epitopes involved in protective immunity.

| ID epitope | **Vaccines** | | | | | | | | | | | | **Vírus circulantes** | | | |
| --- | --- | --- | --- | --- | --- | --- | --- | --- | --- | --- | --- | --- | --- | --- | --- | --- |
|  | **Dengvaxia®** | | | | **LAV-TDV** | | | | **TAK-003** | | | |  |  |  |  |
|  | **DENV1** | **DENV2** | **DENV3** | **DENV4** | **DENV1** | **DENV2** | **DENV3** | **DENV4** | **DENV1** | **DENV2** | **DENV3** | **DENV4** | **DENV1** | **DENV2** | **DENV3** | **DENV4** |
| 001 | 0.00% | 0.00% | 0.00% | 0.00% | **100.00%** | 0.00% | 0.00% | 0.00% | 0.00% | 0.00% | 0.00% | 0.00% | **100.00%** | 0.00% | 0.00% | 0.00% |
| 002 | 0.00% | 0.00% | 0.00% | 0.00% | 0.00% | **100.00%** | 0.00% | **100.00%** | **100.00%** | **100.00%** | **100.00%** | **100.00%** | 0.00% | **100.00%** | 0.00% | **100.00%** |
| 003 | 0.00% | 0.00% | 0.00% | 0.00% | 0.00% | 0.00% | 0.00% | 0.00% | 0.00% | 0.00% | 0.00% | 0.00% | **55.00%** | 0.00% | 0.00% | 0.00% |
| 004 | **100.00%** | 0.00% | 0.00% | 0.00% | **100.00%** | 0.00% | 0.00% | 0.00% | **100.00%** | 0.00% | 0.00% | 0.00% | **78.33%** | 0.00% | 0.00% | 0.00% |
| 005 | **100.00%** | 0.00% | 0.00% | 0.00% | **100.00%** | 0.00% | 0.00% | 0.00% | **100.00%** | 0.00% | 0.00% | 0.00% | **75.00%** | 0.00% | 0.00% | 0.00% |
| 006 | **100.00%** | 0.00% | 0.00% | 0.00% | **100.00%** | 0.00% | 0.00% | 0.00% | 0.00% | 0.00% | 0.00% | 0.00% | **95.00%** | 0.00% | 0.00% | 0.00% |
| 007 | 0.00% | 0.00% | 0.00% | 0.00% | 0.00% | 0.00% | **100.00%** | 0.00% | 0.00% | 0.00% | 0.00% | 0.00% | 0.00% | **86.21%** | **97.83%** | 0.00% |
| 008 | 0.00% | 0.00% | 0.00% | 0.00% | **100.00%** | 0.00% | 0.00% | 0.00% | 0.00% | 0.00% | 0.00% | 0.00% | **98.33%** | 0.00% | 0.00% | 0.00% |
| 009 | 0.00% | 0.00% | 0.00% | 0.00% | **100.00%** | 0.00% | 0.00% | 0.00% | 0.00% | 0.00% | 0.00% | 0.00% | **38.33%** | 0.00% | 0.00% | 0.00% |
| 010 | 0.00% | 0.00% | 0.00% | 0.00% | 0.00% | 0.00% | **100.00%** | 0.00% | **100.00%** | **100.00%** | **100.00%** | **100.00%** | 0.00% | **100.00%** | **97.83%** | 0.00% |
| 011 | 0.00% | 0.00% | 0.00% | 0.00% | **100.00%** | 0.00% | 0.00% | 0.00% | 0.00% | 0.00% | 0.00% | 0.00% | **70.00%** | 0.00% | 0.00% | 0.00% |
| 012 | 0.00% | 0.00% | 0.00% | 0.00% | 0.00% | 0.00% | 0.00% | 0.00% | **100.00%** | **100.00%** | **100.00%** | **100.00%** | 0.00% | **98.28%** | 0.00% | 0.00% |
| 013 | 0.00% | 0.00% | 0.00% | 0.00% | **100.00%** | 0.00% | 0.00% | 0.00% | 0.00% | 0.00% | 0.00% | 0.00% | **91.67%** | 0.00% | 0.00% | 0.00% |
| 014 | 0.00% | 0.00% | 0.00% | 0.00% | 0.00% | 0.00% | 0.00% | 0.00% | **100.00%** | **100.00%** | **100.00%** | **100.00%** | 0.00% | **98.28%** | 0.00% | 0.00% |
| 015 | 0.00% | 0.00% | 0.00% | 0.00% | **100.00%** | 0.00% | **100.00%** | 0.00% | 0.00% | 0.00% | 0.00% | 0.00% | **96.67%** | 0.00% | **100.00%** | 0.00% |
| 016 | 0.00% | 0.00% | 0.00% | 0.00% | **100.00%** | 0.00% | 0.00% | 0.00% | 0.00% | 0.00% | 0.00% | 0.00% | **93.33%** | 0.00% | 0.00% | 0.00% |
| 017 | 0.00% | 0.00% | 0.00% | 0.00% | **100.00%** | 0.00% | **100.00%** | 0.00% | 0.00% | 0.00% | 0.00% | 0.00% | **93.33%** | 0.00% | **97.83%** | 0.00% |
| 018 | 0.00% | 0.00% | 0.00% | 0.00% | **100.00%** | 0.00% | **100.00%** | 0.00% | 0.00% | 0.00% | 0.00% | 0.00% | **98.33%** | 0.00% | **100.00%** | 0.00% |
| 019 | 0.00% | 0.00% | 0.00% | 0.00% | **100.00%** | 0.00% | 0.00% | 0.00% | 0.00% | 0.00% | 0.00% | 0.00% | **96.67%** | 0.00% | 0.00% | 0.00% |
| 020 | 0.00% | 0.00% | 0.00% | 0.00% | **100.00%** | 0.00% | 0.00% | 0.00% | 0.00% | 0.00% | 0.00% | 0.00% | **86.67%** | 0.00% | 0.00% | 0.00% |
| 021 | 0.00% | 0.00% | 0.00% | 0.00% | **100.00%** | 0.00% | 0.00% | 0.00% | 0.00% | 0.00% | 0.00% | 0.00% | **96.67%** | 0.00% | 0.00% | 0.00% |
| 022 | 0.00% | 0.00% | 0.00% | 0.00% | **100.00%** | 0.00% | 0.00% | 0.00% | 0.00% | 0.00% | 0.00% | 0.00% | **100.00%** | 0.00% | 0.00% | 0.00% |
| 023 | 0.00% | 0.00% | 0.00% | 0.00% | **100.00%** | **100.00%** | **100.00%** | **100.00%** | 0.00% | 0.00% | 0.00% | 0.00% | **98.33%** | 0.00% | **100.00%** | **88.00%** |
| 024 | 0.00% | 0.00% | 0.00% | 0.00% | **100.00%** | 0.00% | **100.00%** | 0.00% | 0.00% | 0.00% | 0.00% | 0.00% | **96.67%** | 0.00% | **97.83%** | 0.00% |
| 025 | 0.00% | 0.00% | 0.00% | 0.00% | **100.00%** | 0.00% | **100.00%** | 0.00% | 0.00% | 0.00% | 0.00% | 0.00% | **100.00%** | 0.00% | **95.65%** | 0.00% |
| 026 | 0.00% | 0.00% | 0.00% | 0.00% | **100.00%** | **100.00%** | **100.00%** | **100.00%** | 0.00% | 0.00% | 0.00% | 0.00% | **100.00%** | 0.00% | **100.00%** | **100.00%** |
| 027 | 0.00% | 0.00% | 0.00% | 0.00% | **100.00%** | **100.00%** | **100.00%** | **100.00%** | 0.00% | 0.00% | 0.00% | 0.00% | **100.00%** | 0.00% | **100.00%** | **100.00%** |
| 028 | 0.00% | 0.00% | 0.00% | 0.00% | **100.00%** | **100.00%** | **100.00%** | **100.00%** | 0.00% | 0.00% | 0.00% | 0.00% | **96.67%** | 0.00% | **100.00%** | **100.00%** |
| 029 | 0.00% | 0.00% | 0.00% | 0.00% | **100.00%** | 0.00% | **100.00%** | 0.00% | 0.00% | 0.00% | 0.00% | 0.00% | **100.00%** | 0.00% | **95.65%** | 0.00% |
| 030 | 0.00% | **100.00%** | 0.00% | 0.00% | 0.00% | **100.00%** | 0.00% | 0.00% | 0.00% | **100.00%** | 0.00% | 0.00% | 0.00% | **70.69%** | 0.00% | 0.00% |
| 031 | 0.00% | **100.00%** | 0.00% | 0.00% | 0.00% | **100.00%** | 0.00% | 0.00% | 0.00% | **100.00%** | 0.00% | 0.00% | 0.00% | **67.24%** | 0.00% | 0.00% |
| 032 | 0.00% | 0.00% | 0.00% | 0.00% | 0.00% | 0.00% | 0.00% | 0.00% | 0.00% | 0.00% | 0.00% | 0.00% | 0.00% | 0.00% | 0.00% | **8.00%** |
| 033 | 0.00% | **100.00%** | 0.00% | 0.00% | 0.00% | **100.00%** | 0.00% | 0.00% | 0.00% | **100.00%** | 0.00% | 0.00% | 0.00% | **96.55%** | 0.00% | 0.00% |
| 034 | 0.00% | **100.00%** | 0.00% | 0.00% | 0.00% | **100.00%** | 0.00% | 0.00% | 0.00% | **100.00%** | 0.00% | 0.00% | 0.00% | **93.10%** | 0.00% | 0.00% |
| 035 | 0.00% | **100.00%** | 0.00% | 0.00% | 0.00% | **100.00%** | 0.00% | 0.00% | 0.00% | **100.00%** | 0.00% | 0.00% | 0.00% | **100.00%** | 0.00% | 0.00% |
| 036 | 0.00% | **100.00%** | 0.00% | 0.00% | 0.00% | **100.00%** | 0.00% | 0.00% | 0.00% | **100.00%** | 0.00% | 0.00% | 0.00% | **96.55%** | 0.00% | 0.00% |
| 037 | 0.00% | 0.00% | **100.00%** | 0.00% | 0.00% | 0.00% | **100.00%** | 0.00% | 0.00% | 0.00% | **100.00%** | 0.00% | 0.00% | 0.00% | **100.00%** | 0.00% |
| 038 | 0.00% | 0.00% | 0.00% | 0.00% | 0.00% | 0.00% | **100.00%** | 0.00% | 0.00% | 0.00% | **100.00%** | 0.00% | 0.00% | 0.00% | **89.13%** | 0.00% |
| 039 | 0.00% | 0.00% | **100.00%** | 0.00% | 0.00% | 0.00% | **100.00%** | 0.00% | 0.00% | 0.00% | **100.00%** | 0.00% | 0.00% | 0.00% | **95.65%** | 0.00% |
| 040 | **100.00%** | 0.00% | **100.00%** | 0.00% | 0.00% | 0.00% | **100.00%** | 0.00% | 0.00% | 0.00% | **100.00%** | 0.00% | **5.00%** | 0.00% | **91.30%** | 0.00% |
| 041 | 0.00% | 0.00% | 0.00% | 0.00% | 0.00% | 0.00% | 0.00% | 0.00% | 0.00% | 0.00% | **100.00%** | 0.00% | 0.00% | 0.00% | **63.04%** | 0.00% |
| 042 | 0.00% | 0.00% | 0.00% | 0.00% | 0.00% | 0.00% | **100.00%** | 0.00% | **100.00%** | **100.00%** | **100.00%** | **100.00%** | **3.33%** | **100.00%** | **100.00%** | 0.00% |
| 043 | 0.00% | 0.00% | 0.00% | 0.00% | 0.00% | 0.00% | **100.00%** | 0.00% | 0.00% | 0.00% | 0.00% | 0.00% | 0.00% | 0.00% | **36.96%** | 0.00% |
| 044 | 0.00% | 0.00% | 0.00% | 0.00% | 0.00% | 0.00% | **100.00%** | 0.00% | 0.00% | 0.00% | 0.00% | 0.00% | 0.00% | 0.00% | **91.30%** | 0.00% |
| 045 | 0.00% | 0.00% | 0.00% | 0.00% | 0.00% | 0.00% | 0.00% | 0.00% | 0.00% | 0.00% | 0.00% | 0.00% | 0.00% | 0.00% | **56.52%** | 0.00% |
| 046 | 0.00% | 0.00% | 0.00% | 0.00% | **100.00%** | 0.00% | **100.00%** | 0.00% | **100.00%** | **100.00%** | **100.00%** | **100.00%** | **71.67%** | **100.00%** | **100.00%** | 0.00% |
| 047 | 0.00% | 0.00% | 0.00% | 0.00% | 0.00% | **100.00%** | 0.00% | **100.00%** | 0.00% | 0.00% | 0.00% | 0.00% | 0.00% | 0.00% | 0.00% | **92.00%** |
| 048 | 0.00% | 0.00% | 0.00% | 0.00% | 0.00% | **100.00%** | 0.00% | **100.00%** | 0.00% | 0.00% | 0.00% | 0.00% | 0.00% | 0.00% | 0.00% | **100.00%** |
| 049 | 0.00% | 0.00% | 0.00% | 0.00% | 0.00% | **100.00%** | 0.00% | **100.00%** | 0.00% | 0.00% | 0.00% | 0.00% | 0.00% | **10.34%** | 0.00% | **100.00%** |
| 050 | 0.00% | 0.00% | 0.00% | 0.00% | 0.00% | 0.00% | 0.00% | 0.00% | **100.00%** | **100.00%** | **100.00%** | **100.00%** | 0.00% | **100.00%** | 0.00% | 0.00% |
| 051 | 0.00% | 0.00% | 0.00% | 0.00% | 0.00% | **100.00%** | 0.00% | **100.00%** | 0.00% | 0.00% | 0.00% | 0.00% | 0.00% | 0.00% | 0.00% | **100.00%** |
| 052 | 0.00% | 0.00% | 0.00% | 0.00% | **100.00%** | **100.00%** | 0.00% | **100.00%** | 0.00% | 0.00% | 0.00% | 0.00% | **96.67%** | 0.00% | 0.00% | **96.00%** |
| 053 | 0.00% | 0.00% | 0.00% | 0.00% | 0.00% | **100.00%** | 0.00% | **100.00%** | **100.00%** | **100.00%** | **100.00%** | **100.00%** | 0.00% | **60.34%** | **84.78%** | **100.00%** |
| 054 | 0.00% | 0.00% | 0.00% | 0.00% | 0.00% | 0.00% | 0.00% | 0.00% | 0.00% | 0.00% | 0.00% | 0.00% | 0.00% | 0.00% | 0.00% | **4.00%** |
| 055 | 0.00% | 0.00% | 0.00% | **100.00%** | 0.00% | 0.00% | 0.00% | **100.00%** | 0.00% | 0.00% | 0.00% | **100.00%** | 0.00% | 0.00% | 0.00% | **80.00%** |
| 056 | 0.00% | 0.00% | 0.00% | 0.00% | 0.00% | 0.00% | 0.00% | 0.00% | 0.00% | 0.00% | 0.00% | 0.00% | 0.00% | 0.00% | 0.00% | **24.00%** |
| 057 | 0.00% | 0.00% | 0.00% | 0.00% | 0.00% | **100.00%** | 0.00% | **100.00%** | 0.00% | 0.00% | 0.00% | 0.00% | 0.00% | 0.00% | 0.00% | **80.00%** |
| 058 | 0.00% | 0.00% | 0.00% | 0.00% | 0.00% | **100.00%** | 0.00% | **100.00%** | 0.00% | 0.00% | 0.00% | 0.00% | 0.00% | 0.00% | 0.00% | **68.00%** |
| 059 | 0.00% | 0.00% | 0.00% | 0.00% | 0.00% | **100.00%** | 0.00% | **100.00%** | 0.00% | 0.00% | 0.00% | 0.00% | 0.00% | 0.00% | 0.00% | **84.00%** |
| 060 | 0.00% | 0.00% | 0.00% | 0.00% | **100.00%** | 0.00% | 0.00% | 0.00% | 0.00% | 0.00% | 0.00% | 0.00% | **96.67%** | 0.00% | 0.00% | 0.00% |
| 061 | 0.00% | 0.00% | 0.00% | 0.00% | **100.00%** | 0.00% | 0.00% | 0.00% | **100.00%** | **100.00%** | **100.00%** | **100.00%** | **100.00%** | **100.00%** | 0.00% | 0.00% |
| 062 | 0.00% | 0.00% | 0.00% | 0.00% | 0.00% | 0.00% | 0.00% | **100.00%** | 0.00% | 0.00% | 0.00% | 0.00% | 0.00% | 0.00% | 0.00% | **52.00%** |
| 063 | 0.00% | 0.00% | 0.00% | 0.00% | 0.00% | **100.00%** | 0.00% | **100.00%** | 0.00% | 0.00% | 0.00% | 0.00% | 0.00% | 0.00% | 0.00% | **100.00%** |
| 064 | 0.00% | 0.00% | 0.00% | 0.00% | 0.00% | **100.00%** | 0.00% | **100.00%** | 0.00% | 0.00% | 0.00% | 0.00% | 0.00% | 0.00% | 0.00% | **96.00%** |
| 065 | 0.00% | 0.00% | 0.00% | 0.00% | 0.00% | **100.00%** | 0.00% | **100.00%** | 0.00% | 0.00% | 0.00% | 0.00% | 0.00% | 0.00% | 0.00% | **40.00%** |
| 066 | 0.00% | 0.00% | 0.00% | 0.00% | 0.00% | 0.00% | 0.00% | 0.00% | **100.00%** | **100.00%** | **100.00%** | **100.00%** | 0.00% | **96.55%** | 0.00% | 0.00% |
| 067 | 0.00% | 0.00% | 0.00% | 0.00% | 0.00% | **100.00%** | **100.00%** | **100.00%** | 0.00% | 0.00% | 0.00% | 0.00% | 0.00% | 0.00% | **100.00%** | **96.00%** |
| 068 | 0.00% | 0.00% | 0.00% | 0.00% | 0.00% | 0.00% | 0.00% | 0.00% | 0.00% | 0.00% | 0.00% | 0.00% | 0.00% | 0.00% | **34.78%** | 0.00% |
| 069 | 0.00% | 0.00% | 0.00% | 0.00% | **100.00%** | 0.00% | **100.00%** | 0.00% | **100.00%** | **100.00%** | **100.00%** | **100.00%** | **100.00%** | **100.00%** | **100.00%** | 0.00% |
| 070 | 0.00% | 0.00% | 0.00% | 0.00% | **100.00%** | **100.00%** | **100.00%** | **100.00%** | 0.00% | 0.00% | 0.00% | 0.00% | **98.33%** | 0.00% | **100.00%** | **88.00%** |
| 071 | 0.00% | 0.00% | 0.00% | 0.00% | 0.00% | **100.00%** | 0.00% | **100.00%** | 0.00% | 0.00% | 0.00% | 0.00% | 0.00% | 0.00% | 0.00% | **96.00%** |
| 072 | 0.00% | 0.00% | 0.00% | 0.00% | **100.00%** | 0.00% | 0.00% | 0.00% | **100.00%** | **100.00%** | **100.00%** | **100.00%** | **100.00%** | **100.00%** | 0.00% | 0.00% |
| 073 | 0.00% | 0.00% | 0.00% | 0.00% | **100.00%** | **100.00%** | **100.00%** | **100.00%** | **100.00%** | **100.00%** | **100.00%** | **100.00%** | **100.00%** | **98.28%** | **100.00%** | **96.00%** |
| 074 | 0.00% | 0.00% | 0.00% | 0.00% | 0.00% | **100.00%** | 0.00% | **100.00%** | 0.00% | 0.00% | 0.00% | 0.00% | 0.00% | 0.00% | 0.00% | **100.00%** |
| 075 | 0.00% | 0.00% | 0.00% | 0.00% | 0.00% | 0.00% | 0.00% | 0.00% | 0.00% | 0.00% | 0.00% | 0.00% | 0.00% | 0.00% | 0.00% | **8.00%** |
| 076 | 0.00% | 0.00% | 0.00% | 0.00% | **100.00%** | 0.00% | 0.00% | 0.00% | 0.00% | 0.00% | 0.00% | 0.00% | **86.67%** | 0.00% | 0.00% | 0.00% |
| 077 | 0.00% | 0.00% | 0.00% | 0.00% | 0.00% | 0.00% | 0.00% | 0.00% | 0.00% | 0.00% | 0.00% | 0.00% | 0.00% | 0.00% | **2.17%** | **36.00%** |
| 078 | 0.00% | 0.00% | 0.00% | 0.00% | 0.00% | **100.00%** | **100.00%** | **100.00%** | **100.00%** | **100.00%** | **100.00%** | **100.00%** | 0.00% | **100.00%** | **100.00%** | **100.00%** |
| 079 | 0.00% | 0.00% | 0.00% | 0.00% | 0.00% | **100.00%** | 0.00% | **100.00%** | 0.00% | 0.00% | 0.00% | 0.00% | 0.00% | 0.00% | **4.35%** | **100.00%** |
| 080 | 0.00% | 0.00% | 0.00% | 0.00% | **100.00%** | 0.00% | **100.00%** | 0.00% | 0.00% | 0.00% | 0.00% | 0.00% | **95.00%** | 0.00% | **97.83%** | 0.00% |
| 081 | 0.00% | 0.00% | 0.00% | 0.00% | **100.00%** | **100.00%** | **100.00%** | **100.00%** | **100.00%** | **100.00%** | **100.00%** | **100.00%** | **100.00%** | **100.00%** | **100.00%** | **100.00%** |
| 082 | 0.00% | 0.00% | 0.00% | 0.00% | **100.00%** | **100.00%** | **100.00%** | **100.00%** | 0.00% | 0.00% | 0.00% | 0.00% | **96.67%** | 0.00% | **100.00%** | **100.00%** |
| 083 | 0.00% | 0.00% | 0.00% | 0.00% | 0.00% | **100.00%** | 0.00% | **100.00%** | 0.00% | 0.00% | 0.00% | 0.00% | 0.00% | 0.00% | 0.00% | **100.00%** |
| 084 | 0.00% | 0.00% | 0.00% | 0.00% | **100.00%** | 0.00% | **100.00%** | 0.00% | **100.00%** | **100.00%** | **100.00%** | **100.00%** | **100.00%** | **100.00%** | **100.00%** | 0.00% |
| 085 | 0.00% | 0.00% | 0.00% | 0.00% | 0.00% | 0.00% | **100.00%** | 0.00% | 0.00% | 0.00% | 0.00% | 0.00% | 0.00% | 0.00% | **86.96%** | 0.00% |
| 086 | 0.00% | 0.00% | 0.00% | 0.00% | **100.00%** | **100.00%** | 0.00% | **100.00%** | 0.00% | 0.00% | 0.00% | 0.00% | **91.67%** | 0.00% | 0.00% | **100.00%** |
| 087 | 0.00% | 0.00% | 0.00% | 0.00% | **100.00%** | 0.00% | 0.00% | 0.00% | **100.00%** | **100.00%** | **100.00%** | **100.00%** | **75.00%** | **94.83%** | 0.00% | 0.00% |
| 088 | **100.00%** | **100.00%** | **100.00%** | **100.00%** | **100.00%** | **100.00%** | **100.00%** | **100.00%** | **100.00%** | **100.00%** | **100.00%** | **100.00%** | **100.00%** | **100.00%** | **100.00%** | **100.00%** |
| 089 | 0.00% | 0.00% | 0.00% | 0.00% | 0.00% | **100.00%** | 0.00% | **100.00%** | 0.00% | 0.00% | 0.00% | 0.00% | 0.00% | 0.00% | 0.00% | **100.00%** |
| 090 | **100.00%** | **100.00%** | **100.00%** | 100.00% | **100.00%** | 0.00% | 100.00% | 0.00% | **100.00%** | **100.00%** | **100.00%** | **100.00%** | **100.00%** | **100.00%** | **100.00%** | 0.00% |
| 091 | 0.00% | 0.00% | 0.00% | 0.00% | **100.00%** | **100.00%** | 0.00% | **100.00%** | **100.00%** | **100.00%** | **100.00%** | **100.00%** | **100.00%** | **100.00%** | **2.17%** | **100.00%** |
| 092 | 0.00% | 0.00% | 0.00% | 0.00% | 0.00% | 0.00% | **100.00%** | 0.00% | 0.00% | 0.00% | 0.00% | 0.00% | 0.00% | 0.00% | **93.48%** | 0.00% |
| 093 | 0.00% | 0.00% | 0.00% | 0.00% | **100.00%** | **100.00%** | **100.00%** | **100.00%** | 0.00% | 0.00% | 0.00% | 0.00% | **98.33%** | 0.00% | **100.00%** | **88.00%** |
| 094 | 0.00% | 0.00% | **100.00%** | 0.00% | 0.00% | 0.00% | **100.00%** | 0.00% | 0.00% | 0.00% | 100.00% | 0.00% | 0.00% | 0.00% | **95.65%** | 0.00% |
| 095 | 0.00% | 0.00% | 0.00% | 0.00% | 0.00% | 0.00% | 0.00% | 0.00% | **100.00%** | **100.00%** | **100.00%** | **100.00%** | 0.00% | **100.00%** | 0.00% | 0.00% |
| 096 | 0.00% | 0.00% | 0.00% | 0.00% | 0.00% | 0.00% | 0.00% | 0.00% | **100.00%** | **100.00%** | **100.00%** | **100.00%** | 0.00% | **91.38%** | 0.00% | 0.00% |
| 097 | 0.00% | 0.00% | 0.00% | 0.00% | 0.00% | 0.00% | 0.00% | 0.00% | **100.00%** | **100.00%** | **100.00%** | **100.00%** | 0.00% | **96.55%** | 0.00% | 0.00% |
| 098 | 0.00% | 0.00% | 0.00% | 0.00% | 0.00% | 0.00% | 0.00% | 0.00% | **100.00%** | **100.00%** | **100.00%** | **100.00%** | 0.00% | **100.00%** | 0.00% | 0.00% |
| 099 | 0.00% | 0.00% | 0.00% | 0.00% | 0.00% | 0.00% | 0.00% | 0.00% | 0.00% | 0.00% | 0.00% | 0.00% | 0.00% | **8.62%** | 0.00% | 0.00% |
| 100 | 0.00% | 0.00% | 0.00% | 0.00% | 0.00% | 0.00% | 0.00% | 0.00% | **100.00%** | **100.00%** | **100.00%** | **100.00%** | 0.00% | **98.28%** | 0.00% | 0.00% |
| 101 | 0.00% | 0.00% | 0.00% | 0.00% | 0.00% | 0.00% | 0.00% | 0.00% | 0.00% | 0.00% | 0.00% | 0.00% | 0.00% | **79.31%** | 0.00% | 0.00% |
| 103 | 0.00% | **100.00%** | 0.00% | 0.00% | 0.00% | **100.00%** | 0.00% | 0.00% | 0.00% | **100.00%** | 0.00% | 0.00% | 0.00% | **93.10%** | 0.00% | 0.00% |
| 104 | 0.00% | **100.00%** | 0.00% | 0.00% | 0.00% | **100.00%** | 0.00% | 0.00% | 0.00% | **100.00%** | 0.00% | 0.00% | 0.00% | **100.00%** | 0.00% | 0.00% |
| 105 | 0.00% | **100.00%** | 0.00% | 0.00% | 0.00% | **100.00%** | 0.00% | 0.00% | 0.00% | **100.00%** | 0.00% | 0.00% | 0.00% | **96.55%** | 0.00% | 0.00% |
| 107 | 0.00% | 0.00% | 0.00% | 0.00% | 0.00% | 0.00% | 0.00% | 0.00% | 0.00% | 0.00% | 0.00% | 0.00% | 0.00% | **29.31%** | 0.00% | 0.00% |
| 108 | 0.00% | 0.00% | 0.00% | 0.00% | 0.00% | 0.00% | **100.00%** | 0.00% | **100.00%** | **100.00%** | **100.00%** | **100.00%** | **3.33%** | **98.28%** | **100.00%** | **4.00%** |
| 110 | 0.00% | 0.00% | 0.00% | 0.00% | 0.00% | 0.00% | 0.00% | 0.00% | **100.00%** | **100.00%** | **100.00%** | **100.00%** | 0.00% | **82.76%** | 0.00% | 0.00% |
| 111 | 0.00% | 0.00% | 0.00% | 0.00% | 0.00% | 0.00% | 0.00% | 0.00% | **100.00%** | **100.00%** | **100.00%** | **100.00%** | 0.00% | **68.97%** | 0.00% | 0.00% |
| 112 | 0.00% | 0.00% | 0.00% | 0.00% | 0.00% | 0.00% | 0.00% | 0.00% | **100.00%** | **100.00%** | **100.00%** | **100.00%** | 0.00% | **77.59%** | 0.00% | 0.00% |
| 113 | 0.00% | 0.00% | 0.00% | 0.00% | 0.00% | 0.00% | 0.00% | 0.00% | 0.00% | 0.00% | 0.00% | 0.00% | 0.00% | **34.48%** | 0.00% | 0.00% |
| 114 | 0.00% | 0.00% | 0.00% | 0.00% | 0.00% | 0.00% | 0.00% | 0.00% | 0.00% | 0.00% | 0.00% | 0.00% | 0.00% | 0.00% | 0.00% | **24.00%** |
| 115 | 0.00% | 0.00% | 0.00% | 0.00% | 0.00% | **100.00%** | 0.00% | **100.00%** | 0.00% | 0.00% | 0.00% | 0.00% | 0.00% | 0.00% | 0.00% | **72.00%** |
| 116 | 0.00% | 0.00% | 0.00% | 0.00% | 0.00% | 0.00% | 0.00% | 0.00% | **100.00%** | **100.00%** | **100.00%** | **100.00%** | 0.00% | **91.38%** | 0.00% | 0.00% |
| 117 | 0.00% | 0.00% | 0.00% | 0.00% | 0.00% | 0.00% | 0.00% | 0.00% | **100.00%** | **100.00%** | **100.00%** | **100.00%** | 0.00% | **98.28%** | 0.00% | 0.00% |
| 118 | 0.00% | 0.00% | 0.00% | 0.00% | 0.00% | 0.00% | **100.00%** | 0.00% | **100.00%** | **100.00%** | **100.00%** | **100.00%** | 0.00% | **93.10%** | **100.00%** | 0.00% |
| 122 | 0.00% | 0.00% | 0.00% | 0.00% | **100.00%** | **100.00%** | 0.00% | **100.00%** | 0.00% | 0.00% | 0.00% | 0.00% | **100.00%** | 0.00% | 0.00% | **84.00%** |
| 123 | 0.00% | 0.00% | 0.00% | 0.00% | 0.00% | 0.00% | 0.00% | 0.00% | **100.00%** | **100.00%** | **100.00%** | **100.00%** | 0.00% | **91.38%** | 0.00% | 0.00% |
| 124 | 0.00% | 0.00% | 0.00% | 0.00% | 0.00% | **100.00%** | 0.00% | **100.00%** | **100.00%** | **100.00%** | **100.00%** | **100.00%** | **36.67%** | **98.28%** | 0.00% | **100.00%** |
| 125 | 0.00% | 0.00% | 0.00% | 0.00% | 0.00% | 0.00% | 0.00% | 0.00% | **100.00%** | **100.00%** | **100.00%** | **100.00%** | 0.00% | **63.79%** | 0.00% | 0.00% |
| 126 | 0.00% | 0.00% | 0.00% | 0.00% | 0.00% | **100.00%** | 0.00% | **100.00%** | **100.00%** | **100.00%** | **100.00%** | **100.00%** | 0.00% | **98.28%** | 0.00% | **100.00%** |
| 127 | 0.00% | 0.00% | 0.00% | 0.00% | 0.00% | 0.00% | 0.00% | 0.00% | 0.00% | 0.00% | 0.00% | 0.00% | 0.00% | **77.59%** | 0.00% | 0.00% |
| 128 | 0.00% | 0.00% | 0.00% | 0.00% | 0.00% | 0.00% | 0.00% | 0.00% | 0.00% | 0.00% | 0.00% | 0.00% | 0.00% | **48.28%** | 0.00% | 0.00% |
| 129 | 0.00% | 0.00% | 0.00% | 0.00% | 0.00% | 0.00% | 0.00% | 0.00% | 0.00% | 0.00% | 0.00% | 0.00% | 0.00% | **48.28%** | 0.00% | 0.00% |
| 130 | 0.00% | 0.00% | 0.00% | 0.00% | 0.00% | 0.00% | 0.00% | 0.00% | **100.00%** | **100.00%** | **100.00%** | **100.00%** | 5.00% | **100.00%** | 0.00% | 0.00% |
| 131 | 0.00% | 0.00% | 0.00% | 0.00% | 0.00% | 0.00% | 0.00% | 0.00% | **100.00%** | **100.00%** | **100.00%** | **100.00%** | 0.00% | **100.00%** | 0.00% | 0.00% |
| 132 | 0.00% | 0.00% | 0.00% | 0.00% | **100.00%** | **100.00%** | 0.00% | **100.00%** | 0.00% | 0.00% | 0.00% | 0.00% | 90.00% | 0.00% | 0.00% | **96.00%** |
| 133 | 0.00% | 0.00% | 0.00% | 0.00% | 0.00% | 0.00% | 0.00% | 0.00% | 0.00% | 0.00% | 0.00% | 0.00% | 0.00% | **34.48%** | 0.00% | 0.00% |
| 134 | 0.00% | 0.00% | 0.00% | 0.00% | 0.00% | 0.00% | 0.00% | 0.00% | 0.00% | 0.00% | 0.00% | 0.00% | 0.00% | 0.00% | **39.13%** | 0.00% |
| 135 | 0.00% | 0.00% | 0.00% | 0.00% | 0.00% | 0.00% | 0.00% | 0.00% | **100.00%** | **100.00%** | **100.00%** | **100.00%** | 0.00% | **87.93%** | 0.00% | 0.00% |
| 136 | 0.00% | 0.00% | 0.00% | 0.00% | 0.00% | 0.00% | 0.00% | 0.00% | 0.00% | 0.00% | 0.00% | 0.00% | **1.67%** | 0.00% | **2.17%** | 0.00% |
| 137 | 0.00% | 0.00% | 0.00% | 0.00% | **100.00%** | 0.00% | **100.00%** | 0.00% | 0.00% | 0.00% | 0.00% | 0.00% | **98.33%** | 0.00% | **100.00%** | 0.00% |
| 138 | 0.00% | 0.00% | 0.00% | 0.00% | 0.00% | 0.00% | 0.00% | 0.00% | 0.00% | 0.00% | 0.00% | 0.00% | 0.00% | **1.72%** | 0.00% | 0.00% |
| 139 | 0.00% | 0.00% | 0.00% | 0.00% | 0.00% | 0.00% | 0.00% | 0.00% | **100.00%** | **100.00%** | **100.00%** | **100.00%** | 0.00% | **96.55%** | 0.00% | 0.00% |
| 140 | 0.00% | **100.00%** | 0.00% | 0.00% | 0.00% | **100.00%** | 0.00% | 0.00% | 0.00% | **100.00%** | 0.00% | 0.00% | 0.00% | **96.55%** | 0.00% | 0.00% |
| 141 | 0.00% | 0.00% | **100.00%** | 0.00% | 0.00% | 0.00% | 0.00% | 0.00% | 0.00% | 0.00% | **100.00%** | 0.00% | 0.00% | 0.00% | **86.96%** | 0.00% |
| 142 | 0.00% | **100.00%** | 0.00% | 0.00% | 0.00% | **100.00%** | 0.00% | 0.00% | 0.00% | **100.00%** | 0.00% | 0.00% | 0.00% | **93.10%** | 0.00% | 0.00% |
| 143 | 0.00% | **100.00%** | 0.00% | 0.00% | 0.00% | **100.00%** | 0.00% | 0.00% | 0.00% | 0.00% | 0.00% | 0.00% | 0.00% | **72.41%** | 0.00% | 0.00% |
| 144 | 0.00% | 0.00% | 0.00% | 0.00% | 0.00% | 0.00% | 0.00% | 0.00% | 0.00% | 0.00% | 0.00% | 0.00% | 0.00% | **3.45%** | 0.00% | 0.00% |
| 145 | 0.00% | 0.00% | 0.00% | 0.00% | 0.00% | 0.00% | 0.00% | 0.00% | 0.00% | 0.00% | 0.00% | 0.00% | 0.00% | **25.86%** | 0.00% | 0.00% |
| 146 | 0.00% | 0.00% | 0.00% | 0.00% | 0.00% | 0.00% | 0.00% | 0.00% | **100.00%** | **100.00%** | **100.00%** | **100.00%** | 0.00% | **84.48%** | 0.00% | 0.00% |
| 147 | 0.00% | 0.00% | 0.00% | 0.00% | 0.00% | 0.00% | 0.00% | 0.00% | **100.00%** | **100.00%** | **100.00%** | **100.00%** | 0.00% | **77.59%** | 0.00% | 0.00% |
| 148 | 0.00% | 0.00% | 0.00% | 0.00% | 0.00% | 0.00% | 0.00% | 0.00% | 0.00% | 0.00% | 0.00% | 0.00% | 0.00% | 0.00% | 0.00% | 0.00% |
| 149 | 0.00% | 0.00% | 0.00% | 0.00% | 0.00% | 0.00% | 0.00% | 0.00% | **100.00%** | **100.00%** | **100.00%** | **100.00%** | 0.00% | **96.55%** | 0.00% | 0.00% |
| 150 | 0.00% | 0.00% | 0.00% | 0.00% | 0.00% | 0.00% | 0.00% | 0.00% | 0.00% | 0.00% | 0.00% | 0.00% | **85.00%** | 0.00% | 0.00% | 0.00% |
| 151 | 0.00% | 0.00% | 0.00% | 0.00% | 0.00% | 0.00% | 0.00% | 0.00% | 0.00% | 0.00% | 0.00% | 0.00% | 0.00% | **48.28%** | 0.00% | 0.00% |
| 152 | 0.00% | 0.00% | 0.00% | 0.00% | 0.00% | 0.00% | **100.00%** | 0.00% | 0.00% | 0.00% | 0.00% | 0.00% | 0.00% | 0.00% | **78.26%** | 0.00% |
| 153 | 0.00% | 0.00% | 0.00% | 0.00% | **100.00%** | 0.00% | 0.00% | 0.00% | 0.00% | 0.00% | 0.00% | 0.00% | **41.67%** | 0.00% | 0.00% | 0.00% |
| 154 | 0.00% | 0.00% | 0.00% | 0.00% | 0.00% | 0.00% | 0.00% | 0.00% | **100.00%** | **100.00%** | **100.00%** | **100.00%** | 0.00% | **94.83%** | 0.00% | 0.00% |
| 155 | 0.00% | 0.00% | 0.00% | 0.00% | 0.00% | 0.00% | 0.00% | 0.00% | **100.00%** | **100.00%** | **100.00%** | **100.00%** | 0.00% | **100.00%** | 0.00% | 0.00% |
| 156 | 0.00% | 0.00% | 0.00% | 0.00% | 0.00% | 0.00% | 0.00% | 0.00% | 0.00% | 0.00% | 0.00% | 0.00% | **1.67%** | 0.00% | **45.65%** | 0.00% |
| 157 | 0.00% | 0.00% | 0.00% | 0.00% | 0.00% | 0.00% | 0.00% | 0.00% | **100.00%** | **100.00%** | **100.00%** | **100.00%** | 0.00% | **98.28%** | 0.00% | 0.00% |
| 158 | 0.00% | 0.00% | 0.00% | 0.00% | 0.00% | 0.00% | 0.00% | 0.00% | **100.00%** | **100.00%** | **100.00%** | **100.00%** | 0.00% | **56.90%** | 0.00% | 0.00% |
| 159 | 0.00% | 0.00% | 0.00% | 0.00% | 0.00% | 0.00% | 0.00% | 0.00% | **100.00%** | **100.00%** | **100.00%** | **100.00%** | 0.00% | **98.28%** | 0.00% | 0.00% |
| 160 | 0.00% | 0.00% | 0.00% | 0.00% | **100.00%** | 0.00% | 0.00% | 0.00% | 0.00% | 0.00% | 0.00% | 0.00% | **98.33%** | 0.00% | 0.00% | 0.00% |
| 161 | 0.00% | 0.00% | 0.00% | 0.00% | 0.00% | 0.00% | 0.00% | 0.00% | 0.00% | 0.00% | 0.00% | 0.00% | 0.00% | 0.00% | 0.00% | 0.00% |
| 162 | 0.00% | 0.00% | 0.00% | 0.00% | 0.00% | 0.00% | 0.00% | 0.00% | **100.00%** | **100.00%** | **100.00%** | **100.00%** | 0.00% | **81.03%** | 0.00% | 0.00% |
| 163 | 0.00% | 0.00% | 0.00% | 0.00% | **100.00%** | 0.00% | 0.00% | 0.00% | 0.00% | 0.00% | 0.00% | 0.00% | **98.33%** | 0.00% | 0.00% | 0.00% |
| 164 | 0.00% | 0.00% | 0.00% | 0.00% | 0.00% | 0.00% | 0.00% | 0.00% | 0.00% | 0.00% | 0.00% | 0.00% | 0.00% | 0.00% | **84.78%** | 0.00% |
| 165 | 0.00% | 0.00% | 0.00% | 0.00% | **100.00%** | 0.00% | 0.00% | 0.00% | 0.00% | 0.00% | 0.00% | 0.00% | **28.33%** | 0.00% | 0.00% | 0.00% |
| 166 | 0.00% | 0.00% | 0.00% | 0.00% | **100.00%** | 0.00% | 0.00% | 0.00% | 0.00% | 0.00% | 0.00% | 0.00% | **98.33%** | 0.00% | 0.00% | 0.00% |
| 167 | 0.00% | 0.00% | 0.00% | 0.00% | **100.00%** | 0.00% | **100.00%** | 0.00% | **100.00%** | **100.00%** | **100.00%** | **100.00%** | **98.33%** | **100.00%** | **100.00%** | 0.00% |
| 168 | 0.00% | 0.00% | 0.00% | 0.00% | 0.00% | **100.00%** | 0.00% | **100.00%** | **100.00%** | **100.00%** | **100.00%** | **100.00%** | **36.67%** | **98.28%** | 0.00% | **100.00%** |
| 169 | 0.00% | 0.00% | 0.00% | 0.00% | **100.00%** | 0.00% | **100.00%** | 0.00% | 0.00% | 0.00% | 0.00% | 0.00% | **63.33%** | 0.00% | **100.00%** | 0.00% |
| 170 | 0.00% | 0.00% | 0.00% | 0.00% | 0.00% | 0.00% | 0.00% | 0.00% | **100.00%** | **100.00%** | **100.00%** | **100.00%** | 0.00% | **94.83%** | 0.00% | 0.00% |
| 173 | 0.00% | 0.00% | 0.00% | 0.00% | 0.00% | 0.00% | 0.00% | 0.00% | 0.00% | 0.00% | 0.00% | 0.00% | 0.00% | 0.00% | **52.17%** | 0.00% |
| 174 | 0.00% | 0.00% | 0.00% | 0.00% | 0.00% | **100.00%** | 0.00% | **100.00%** | 0.00% | 0.00% | 0.00% | 0.00% | 0.00% | 0.00% | 0.00% | **100.00%** |
| 175 | 0.00% | 0.00% | 0.00% | 0.00% | 0.00% | 0.00% | 0.00% | 0.00% | 0.00% | 0.00% | 0.00% | 0.00% | 0.00% | 0.00% | 0.00% | **80.00%** |

Supplementary table 6– Results of epitope scores grouped by location on the polyprotein.

| **Protein** | **ID** | **HLA class** | **Percentage of Conservation** | | | | | | | **Scores** | | | | | |
| --- | --- | --- | --- | --- | --- | --- | --- | --- | --- | --- | --- | --- | --- | --- | --- |
|  |  |  | **in circulating viruses** | | | | **in vaccines** | | | **without normalization** | | | **with normalization** | | |
|  |  |  | **DENV1** | **DENV2** | **DENV3** | **DENV4** | **DENGVAXIA** | **TAK-003** | **LAV-TDV** | **DENGVAXIA®** | **TAK-003** | **LAV-TDV** | **DENGVAXIA** | **TAK-003** | **LAV-TDV** |
|  |  |  |  |  |  |  |  |  |  |  |  |  |  |  |  |
| PROTEIN C | 001 | I | 100.0% | 0.0% | 0.0% | 0.0% | 0.0% | 0.0% | 100.0% | 0.00 | 0.00 | 0.25 | 0.000 | 0.000 | 0.258 |
|  | 002 | I | 50.0% | 50.0% | 0.0% | 0.0% | 0.0% | 100.0% | 100.0% | 0.00 | 0.25 | 0.25 | 0.000 | 0.505 | 0.505 |
|  | 062 | I | 0.0% | 0.0% | 0.0% | 52.0% | 0.0% | 0.0% | 100.0% | 0.00 | 0.00 | 0.13 | 0.000 | 0.000 | 0.035 |
|  | 024 | I | 50.0% | 0.0% | 50.0% | 0.0% | 0.0% | 0.0% | 100.0% | 0.00 | 0.00 | 0.25 | 0.000 | 0.000 | 0.055 |
|  | 095 | II | 0.0% | 100.0% | 0.0% | 0.0% | 0.0% | 100.0% | 0.0% | 0.00 | 0.25 | 0.00 | 0.000 | 0.258 | 0.000 |
|  | 139 | II | 0.0% | 96.6% | 0.0% | 0.0% | 0.0% | 100.0% | 0.0% | 0.00 | 0.24 | 0.00 | 0.000 | 0.221 | 0.000 |
| PROTEIN prM | 037 | I | 0.0% | 0.0% | 100.0% | 0.0% | 100.0% | 100.0% | 100.0% | 0.25 | 0.25 | 0.25 | 0.258 | 0.258 | 0.258 |
|  | 038 | I | 0.0% | 0.0% | 89.1% | 0.0% | 0.0% | 100.0% | 100.0% | 0.00 | 0.22 | 0.22 | 0.000 | 0.159 | 0.159 |
|  | 143 | II | 0.0% | 72.4% | 0.0% | 0.0% | 100.0% | 0.0% | 100.0% | 0.18 | 0.00 | 0.18 | 0.078 | 0.000 | 0.078 |
| PROTEIN E | 004 | I | 78.3% | 0.0% | 0.0% | 0.0% | 100.0% | 100.0% | 100.0% | 0.20 | 0.20 | 0.20 | 0.100 | 0.100 | 0.100 |
|  | 005 | I | 75.0% | 0.0% | 0.0% | 0.0% | 100.0% | 100.0% | 100.0% | 0.19 | 0.19 | 0.19 | 0.087 | 0.087 | 0.087 |
|  | 006 | I | 95.0% | 0.0% | 0.0% | 0.0% | 100.0% | 0.0% | 100.0% | 0.24 | 0.00 | 0.24 | 0.206 | 0.000 | 0.206 |
|  | 030 | I | 0.0% | 70.7% | 0.0% | 0.0% | 100.0% | 100.0% | 100.0% | 0.18 | 0.18 | 0.18 | 0.072 | 0.072 | 0.072 |
|  | 031 | I | 0.0% | 67.2% | 0.0% | 0.0% | 100.0% | 100.0% | 100.0% | 0.17 | 0.17 | 0.17 | 0.063 | 0.063 | 0.063 |
|  | 033 | I | 0.0% | 96.6% | 0.0% | 0.0% | 100.0% | 100.0% | 100.0% | 0.24 | 0.24 | 0.24 | 0.221 | 0.221 | 0.221 |
|  | 034 | I | 0.0% | 93.1% | 0.0% | 0.0% | 100.0% | 100.0% | 100.0% | 0.23 | 0.23 | 0.23 | 0.189 | 0.189 | 0.189 |
|  | 035 | I | 0.0% | 100.0% | 0.0% | 0.0% | 100.0% | 100.0% | 100.0% | 0.25 | 0.25 | 0.25 | 0.258 | 0.258 | 0.258 |
|  | 036 | I | 0.0% | 96.6% | 0.0% | 0.0% | 100.0% | 100.0% | 100.0% | 0.24 | 0.24 | 0.24 | 0.221 | 0.221 | 0.221 |
|  | 039 | I | 0.0% | 0.0% | 95.7% | 0.0% | 100.0% | 100.0% | 100.0% | 0.24 | 0.24 | 0.24 | 0.212 | 0.212 | 0.212 |
|  | 040 | I | 5.0% | 0.0% | 91.3% | 0.0% | 100.0% | 100.0% | 100.0% | 0.24 | 0.24 | 0.24 | 0.176 | 0.176 | 0.176 |
|  | 041 | I | 0.0% | 0.0% | 63.0% | 0.0% | 0.0% | 100.0% | 0.0% | 0.00 | 0.16 | 0.00 | 0.000 | 0.053 | 0.000 |
|  | 055 | I | 0.0% | 0.0% | 0.0% | 80.0% | 100.0% | 100.0% | 100.0% | 0.20 | 0.20 | 0.20 | 0.107 | 0.107 | 0.107 |
|  | 094 | I | 0.0% | 0.0% | 95.7% | 0.0% | 100.0% | 0.0% | 100.0% | 0.24 | 0.00 | 0.24 | 0.212 | 0.000 | 0.212 |
|  | 103 | I | 0.0% | 93.1% | 0.0% | 0.0% | 100.0% | 100.0% | 100.0% | 0.23 | 0.23 | 0.23 | 0.189 | 0.189 | 0.189 |
|  | 104 | I | 0.0% | 100.0% | 0.0% | 0.0% | 100.0% | 100.0% | 100.0% | 0.25 | 0.25 | 0.25 | 0.258 | 0.258 | 0.258 |
|  | 105 | I | 0.0% | 96.6% | 0.0% | 0.0% | 100.0% | 100.0% | 100.0% | 0.24 | 0.24 | 0.24 | 0.221 | 0.221 | 0.221 |
|  | 140 | II | 0.0% | 96.6% | 0.0% | 0.0% | 100.0% | 100.0% | 100.0% | 0.24 | 0.24 | 0.24 | 0.221 | 0.221 | 0.221 |
|  | 141 | II | 0.0% | 0.0% | 87.0% | 0.0% | 100.0% | 100.0% | 0.0% | 0.22 | 0.22 | 0.00 | 0.145 | 0.145 | 0.000 |
|  | 142 | II | 0.0% | 93.1% | 0.0% | 0.0% | 100.0% | 100.0% | 100.0% | 0.23 | 0.23 | 0.23 | 0.189 | 0.189 | 0.189 |
| PROTEIN NS1 | 007 | I | 0.0% | 86.2% | 97.8% | 0.0% | 0.0% | 0.0% | 100.0% | 0.00 | 0.00 | 0.46 | 0.000 | 0.000 | 0.364 |
|  | 025 | I | 100.0% | 0.0% | 95.7% | 0.0% | 0.0% | 0.0% | 100.0% | 0.00 | 0.00 | 0.49 | 0.000 | 0.000 | 0.460 |
|  | 042 | I | 3.3% | 100.0% | 100.0% | 0.0% | 0.0% | 100.0% | 100.0% | 0.00 | 0.51 | 0.51 | 0.000 | 0.505 | 0.505 |
|  | 043 | I | 0.0% | 0.0% | 37.0% | 0.0% | 0.0% | 0.0% | 100.0% | 0.00 | 0.00 | 0.09 | 0.000 | 0.000 | 0.021 |
|  | 057 | I | 0.0% | 0.0% | 0.0% | 80.0% | 0.0% | 0.0% | 100.0% | 0.00 | 0.00 | 0.20 | 0.000 | 0.000 | 0.107 |
|  | 073 | I | 100.0% | 98.3% | 100.0% | 96.0% | 0.0% | 100.0% | 100.0% | 0.00 | 0.99 | 0.99 | 0.000 | 0.939 | 0.939 |
|  | 108 | I | 3.3% | 98.3% | 100.0% | 4.0% | 0.0% | 100.0% | 100.0% | 0.00 | 0.51 | 0.51 | 0.000 | 0.487 | 0.487 |
|  | 146 | II | 0.0% | 84.5% | 0.0% | 0.0% | 0.0% | 100.0% | 0.0% | 0.00 | 0.21 | 0.00 | 0.000 | 0.130 | 0.000 |
|  | 147 | II | 0.0% | 77.6% | 0.0% | 0.0% | 0.0% | 100.0% | 0.0% | 0.00 | 0.19 | 0.00 | 0.000 | 0.097 | 0.000 |
| PROTEIN NS2A | 008 | I | 98.3% | 0.0% | 0.0% | 0.0% | 0.0% | 0.0% | 100.0% | 0.00 | 0.00 | 0.25 | 0.000 | 0.000 | 0.239 |
|  | 009 | I | 38.3% | 0.0% | 0.0% | 0.0% | 0.0% | 0.0% | 100.0% | 0.00 | 0.00 | 0.10 | 0.000 | 0.000 | 0.022 |
|  | 044 | I | 0.0% | 0.0% | 91.3% | 0.0% | 0.0% | 0.0% | 100.0% | 0.00 | 0.00 | 0.23 | 0.000 | 0.000 | 0.175 |
|  | 058 | I | 0.0% | 0.0% | 0.0% | 68.0% | 0.0% | 0.0% | 100.0% | 0.00 | 0.00 | 0.17 | 0.000 | 0.000 | 0.065 |
|  | 063 | I | 0.0% | 0.0% | 0.0% | 100.0% | 0.0% | 0.0% | 100.0% | 0.00 | 0.00 | 0.25 | 0.000 | 0.000 | 0.258 |
|  | 074 | I | 0.0% | 0.0% | 0.0% | 100.0% | 0.0% | 0.0% | 100.0% | 0.00 | 0.00 | 0.25 | 0.000 | 0.000 | 0.258 |
|  | 110 | I | 0.0% | 82.8% | 0.0% | 0.0% | 0.0% | 100.0% | 0.0% | 0.00 | 0.21 | 0.00 | 0.000 | 0.121 | 0.000 |
|  | 111 | I | 0.0% | 69.0% | 0.0% | 0.0% | 0.0% | 100.0% | 0.0% | 0.00 | 0.17 | 0.00 | 0.000 | 0.067 | 0.000 |
|  | 112 | I | 0.0% | 77.6% | 0.0% | 0.0% | 0.0% | 100.0% | 0.0% | 0.00 | 0.19 | 0.00 | 0.000 | 0.097 | 0.000 |
|  | 115 | I | 0.0% | 0.0% | 0.0% | 72.0% | 0.0% | 0.0% | 100.0% | 0.00 | 0.00 | 0.18 | 0.000 | 0.000 | 0.076 |
|  | 116 | I | 0.0% | 91.4% | 0.0% | 0.0% | 0.0% | 100.0% | 0.0% | 0.00 | 0.23 | 0.00 | 0.000 | 0.176 | 0.000 |
|  | 096 | II | 0.0% | 91.4% | 0.0% | 0.0% | 0.0% | 100.0% | 0.0% | 0.00 | 0.23 | 0.00 | 0.000 | 0.176 | 0.000 |
|  | 149 | II | 0.0% | 96.6% | 0.0% | 0.0% | 0.0% | 100.0% | 0.0% | 0.00 | 0.24 | 0.00 | 0.000 | 0.221 | 0.000 |
|  | 152 | II | 0.0% | 0.0% | 78.3% | 0.0% | 0.0% | 0.0% | 100.0% | 0.00 | 0.00 | 0.20 | 0.000 | 0.000 | 0.099 |
| PROTEIN NS2B | 059 | I | 0.0% | 0.0% | 0.0% | 84.0% | 0.0% | 0.0% | 100.0% | 0.00 | 0.00 | 0.21 | 0.000 | 0.000 | 0.127 |
|  | 064 | I | 0.0% | 0.0% | 0.0% | 96.0% | 0.0% | 0.0% | 100.0% | 0.00 | 0.00 | 0.24 | 0.000 | 0.000 | 0.215 |
|  | 117 | I | 0.0% | 98.3% | 0.0% | 0.0% | 0.0% | 100.0% | 0.0% | 0.00 | 0.25 | 0.00 | 0.000 | 0.238 | 0.000 |
|  | 153 | II | 41.7% | 0.0% | 0.0% | 0.0% | 0.0% | 0.0% | 100.0% | 0.00 | 0.00 | 0.10 | 0.000 | 0.000 | 0.025 |
|  | 154 | II | 0.0% | 94.8% | 0.0% | 0.0% | 0.0% | 100.0% | 0.0% | 0.00 | 0.24 | 0.00 | 0.000 | 0.205 | 0.000 |
| PROTEIN NS3 | 011 | I | 70.0% | 0.0% | 0.0% | 0.0% | 0.0% | 0.0% | 100.0% | 0.00 | 0.00 | 0.18 | 0.000 | 0.000 | 0.070 |
|  | 012 | I | 0.0% | 98.3% | 0.0% | 0.0% | 0.0% | 100.0% | 0.0% | 0.00 | 0.25 | 0.00 | 0.000 | 0.238 | 0.000 |
|  | 013 | I | 91.7% | 0.0% | 0.0% | 0.0% | 0.0% | 0.0% | 100.0% | 0.00 | 0.00 | 0.23 | 0.000 | 0.000 | 0.178 |
|  | 014 | I | 0.0% | 98.3% | 0.0% | 0.0% | 0.0% | 100.0% | 0.0% | 0.00 | 0.25 | 0.00 | 0.000 | 0.238 | 0.000 |
|  | 015 | I | 96.7% | 0.0% | 100.0% | 0.0% | 0.0% | 0.0% | 100.0% | 0.00 | 0.00 | 0.49 | 0.000 | 0.000 | 0.469 |
|  | 016 | I | 93.3% | 0.0% | 0.0% | 0.0% | 0.0% | 0.0% | 100.0% | 0.00 | 0.00 | 0.23 | 0.000 | 0.000 | 0.191 |
|  | 026 | I | 100.0% | 0.0% | 100.0% | 0.0% | 0.0% | 0.0% | 100.0% | 0.00 | 0.00 | 0.50 | 0.000 | 0.000 | 0.505 |
|  | 027 | I | 100.0% | 0.0% | 100.0% | 0.0% | 0.0% | 0.0% | 100.0% | 0.00 | 0.00 | 0.50 | 0.000 | 0.000 | 0.505 |
|  | 028 | I | 96.7% | 0.0% | 100.0% | 0.0% | 0.0% | 0.0% | 100.0% | 0.00 | 0.00 | 0.49 | 0.000 | 0.000 | 0.469 |
|  | 029 | I | 100.0% | 0.0% | 95.7% | 0.0% | 0.0% | 0.0% | 100.0% | 0.00 | 0.00 | 0.49 | 0.000 | 0.000 | 0.460 |
|  | 046 | I | 71.7% | 100.0% | 100.0% | 0.0% | 0.0% | 100.0% | 100.0% | 0.00 | 0.68 | 0.68 | 0.000 | 0.570 | 0.570 |
|  | 047 | I | 0.0% | 0.0% | 0.0% | 92.0% | 0.0% | 0.0% | 100.0% | 0.00 | 0.00 | 0.23 | 0.000 | 0.000 | 0.180 |
|  | 048 | I | 0.0% | 0.0% | 0.0% | 100.0% | 0.0% | 0.0% | 100.0% | 0.00 | 0.00 | 0.25 | 0.000 | 0.000 | 0.258 |
|  | 049 | I | 0.0% | 10.3% | 0.0% | 100.0% | 0.0% | 0.0% | 100.0% | 0.00 | 0.00 | 0.28 | 0.000 | 0.000 | 0.259 |
|  | 050 | I | 0.0% | 100.0% | 0.0% | 0.0% | 0.0% | 100.0% | 0.0% | 0.00 | 0.25 | 0.00 | 0.000 | 0.258 | 0.000 |
|  | 060 | I | 96.7% | 0.0% | 0.0% | 0.0% | 0.0% | 0.0% | 100.0% | 0.00 | 0.00 | 0.24 | 0.000 | 0.000 | 0.222 |
|  | 065 | I | 0.0% | 0.0% | 0.0% | 40.0% | 0.0% | 0.0% | 100.0% | 0.00 | 0.00 | 0.10 | 0.000 | 0.000 | 0.023 |
|  | 067 | I | 0.0% | 0.0% | 100.0% | 96.0% | 0.0% | 0.0% | 100.0% | 0.00 | 0.00 | 0.49 | 0.000 | 0.000 | 0.463 |
|  | 071 | I | 0.0% | 0.0% | 0.0% | 96.0% | 0.0% | 0.0% | 100.0% | 0.00 | 0.00 | 0.24 | 0.000 | 0.000 | 0.215 |
|  | 076 | I | 86.7% | 0.0% | 0.0% | 0.0% | 0.0% | 0.0% | 100.0% | 0.00 | 0.00 | 0.22 | 0.000 | 0.000 | 0.143 |
|  | 078 | I | 0.0% | 100.0% | 100.0% | 100.0% | 0.0% | 100.0% | 100.0% | 0.00 | 0.75 | 0.75 | 0.000 | 0.753 | 0.753 |
|  | 079 | I | 0.0% | 0.0% | 4.4% | 100.0% | 0.0% | 0.0% | 100.0% | 0.00 | 0.00 | 0.26 | 0.000 | 0.000 | 0.258 |
|  | 080 | I | 95.0% | 0.0% | 97.8% | 0.0% | 0.0% | 0.0% | 100.0% | 0.00 | 0.00 | 0.48 | 0.000 | 0.000 | 0.430 |
|  | 081 | I | 100.0% | 100.0% | 100.0% | 100.0% | 0.0% | 100.0% | 100.0% | 0.00 | 1.00 | 1.00 | 0.000 | 1.000 | 1.000 |
|  | 082 | I | 96.7% | 0.0% | 100.0% | 100.0% | 0.0% | 0.0% | 100.0% | 0.00 | 0.00 | 0.74 | 0.000 | 0.000 | 0.717 |
|  | 083 | I | 0.0% | 0.0% | 0.0% | 100.0% | 0.0% | 0.0% | 100.0% | 0.00 | 0.00 | 0.25 | 0.000 | 0.000 | 0.258 |
|  | 118 | I | 0.0% | 93.1% | 100.0% | 0.0% | 0.0% | 100.0% | 100.0% | 0.00 | 0.48 | 0.48 | 0.000 | 0.437 | 0.437 |
|  | 166 | I | 98.3% | 0.0% | 0.0% | 0.0% | 0.0% | 0.0% | 100.0% | 0.00 | 0.00 | 0.25 | 0.000 | 0.000 | 0.239 |
|  | 167 | I | 98.3% | 100.0% | 100.0% | 0.0% | 0.0% | 100.0% | 100.0% | 0.00 | 0.75 | 0.75 | 0.000 | 0.734 | 0.734 |
|  | 170 | I | 0.0% | 94.8% | 0.0% | 0.0% | 0.0% | 100.0% | 0.0% | 0.00 | 0.24 | 0.00 | 0.000 | 0.205 | 0.000 |
|  | 174 | I | 0.0% | 0.0% | 0.0% | 100.0% | 0.0% | 0.0% | 100.0% | 0.00 | 0.00 | 0.25 | 0.000 | 0.000 | 0.258 |
|  | 100 | II | 0.0% | 98.3% | 0.0% | 0.0% | 0.0% | 100.0% | 0.0% | 0.00 | 0.25 | 0.00 | 0.000 | 0.238 | 0.000 |
|  | 155 | II | 0.0% | 100.0% | 0.0% | 0.0% | 0.0% | 100.0% | 0.0% | 0.00 | 0.25 | 0.00 | 0.000 | 0.258 | 0.000 |
|  | 157 | II | 0.0% | 98.3% | 0.0% | 0.0% | 0.0% | 100.0% | 0.0% | 0.00 | 0.25 | 0.00 | 0.000 | 0.238 | 0.000 |
|  | 158 | II | 0.0% | 56.9% | 0.0% | 0.0% | 0.0% | 100.0% | 0.0% | 0.00 | 0.14 | 0.00 | 0.000 | 0.042 | 0.000 |
|  | 159 | II | 0.0% | 98.3% | 0.0% | 0.0% | 0.0% | 100.0% | 0.0% | 0.00 | 0.25 | 0.00 | 0.000 | 0.238 | 0.000 |
|  | 160 | II | 98.3% | 0.0% | 0.0% | 0.0% | 0.0% | 0.0% | 100.0% | 0.00 | 0.00 | 0.25 | 0.000 | 0.000 | 0.239 |
| PROTEIN NS4A | 051 | I | 0.0% | 0.0% | 0.0% | 100.0% | 0.0% | 0.0% | 100.0% | 0.00 | 0.00 | 0.25 | 0.000 | 0.000 | 0.258 |
|  | 122 | I | 100.0% | 0.0% | 0.0% | 84.0% | 0.0% | 0.0% | 100.0% | 0.00 | 0.00 | 0.46 | 0.000 | 0.000 | 0.375 |
|  | 162 | II | 0.0% | 81.0% | 0.0% | 0.0% | 0.0% | 100.0% | 0.0% | 0.00 | 0.20 | 0.00 | 0.000 | 0.112 | 0.000 |
| PROTEIN NS4B | 010 | I | 0.0% | 100.0% | 97.8% | 0.0% | 0.0% | 100.0% | 100.0% | 0.00 | 0.49 | 0.49 | 0.000 | 0.481 | 0.481 |
|  | 017 | I | 93.3% | 0.0% | 97.8% | 0.0% | 0.0% | 0.0% | 100.0% | 0.00 | 0.00 | 0.48 | 0.000 | 0.000 | 0.415 |
|  | 018 | I | 98.3% | 0.0% | 100.0% | 0.0% | 0.0% | 0.0% | 100.0% | 0.00 | 0.00 | 0.50 | 0.000 | 0.000 | 0.486 |
|  | 069 | I | 100.0% | 100.0% | 100.0% | 0.0% | 0.0% | 100.0% | 100.0% | 0.00 | 0.75 | 0.75 | 0.000 | 0.753 | 0.753 |
|  | 084 | I | 100.0% | 100.0% | 100.0% | 0.0% | 0.0% | 100.0% | 100.0% | 0.00 | 0.75 | 0.75 | 0.000 | 0.753 | 0.753 |
|  | 085 | I | 0.0% | 0.0% | 87.0% | 0.0% | 0.0% | 0.0% | 100.0% | 0.00 | 0.00 | 0.22 | 0.000 | 0.000 | 0.145 |
|  | 123 | I | 0.0% | 91.4% | 0.0% | 0.0% | 0.0% | 100.0% | 0.0% | 0.00 | 0.23 | 0.00 | 0.000 | 0.176 | 0.000 |
|  | 124 | I | 36.7% | 98.3% | 0.0% | 100.0% | 0.0% | 100.0% | 100.0% | 0.00 | 0.59 | 0.59 | 0.000 | 0.497 | 0.497 |
|  | 125 | I | 0.0% | 63.8% | 0.0% | 0.0% | 0.0% | 100.0% | 0.0% | 0.00 | 0.16 | 0.00 | 0.000 | 0.055 | 0.000 |
|  | 126 | I | 0.0% | 98.3% | 0.0% | 100.0% | 0.0% | 100.0% | 100.0% | 0.00 | 0.50 | 0.50 | 0.000 | 0.486 | 0.486 |
|  | 168 | I | 36.7% | 98.3% | 0.0% | 100.0% | 0.0% | 100.0% | 100.0% | 0.00 | 0.59 | 0.59 | 0.000 | 0.497 | 0.497 |
|  | 169 | I | 63.3% | 0.0% | 100.0% | 0.0% | 0.0% | 0.0% | 100.0% | 0.00 | 0.00 | 0.41 | 0.000 | 0.000 | 0.301 |
|  | 097 | II | 0.0% | 96.6% | 0.0% | 0.0% | 0.0% | 100.0% | 0.0% | 0.00 | 0.24 | 0.00 | 0.000 | 0.221 | 0.000 |
|  | 163 | II | 98.3% | 0.0% | 0.0% | 0.0% | 0.0% | 0.0% | 100.0% | 0.00 | 0.00 | 0.25 | 0.000 | 0.000 | 0.239 |
| PROTEIN NS5 | 019 | I | 96.7% | 0.0% | 0.0% | 0.0% | 0.0% | 0.0% | 100.0% | 0.00 | 0.00 | 0.24 | 0.000 | 0.000 | 0.222 |
|  | 020 | I | 86.7% | 0.0% | 0.0% | 0.0% | 0.0% | 0.0% | 100.0% | 0.00 | 0.00 | 0.22 | 0.000 | 0.000 | 0.143 |
|  | 021 | I | 96.7% | 0.0% | 0.0% | 0.0% | 0.0% | 0.0% | 100.0% | 0.00 | 0.00 | 0.24 | 0.000 | 0.000 | 0.222 |
|  | 022 | I | 100.0% | 0.0% | 0.0% | 0.0% | 0.0% | 0.0% | 100.0% | 0.00 | 0.00 | 0.25 | 0.000 | 0.000 | 0.258 |
|  | 023 | I | 98.3% | 0.0% | 100.0% | 88.0% | 0.0% | 0.0% | 100.0% | 0.00 | 0.00 | 0.72 | 0.000 | 0.000 | 0.628 |
|  | 052 | I | 96.7% | 0.0% | 0.0% | 96.0% | 0.0% | 0.0% | 100.0% | 0.00 | 0.00 | 0.48 | 0.000 | 0.000 | 0.427 |
|  | 053 | I | 0.0% | 60.3% | 84.8% | 100.0% | 0.0% | 100.0% | 100.0% | 0.00 | 0.61 | 0.61 | 0.000 | 0.417 | 0.417 |
|  | 061 | I | 100.0% | 100.0% | 0.0% | 0.0% | 0.0% | 100.0% | 100.0% | 0.00 | 0.50 | 0.50 | 0.000 | 0.505 | 0.505 |
|  | 066 | I | 0.0% | 96.6% | 0.0% | 0.0% | 0.0% | 100.0% | 0.0% | 0.00 | 0.24 | 0.00 | 0.000 | 0.221 | 0.000 |
|  | 070 | I | 98.3% | 0.0% | 100.0% | 88.0% | 0.0% | 0.0% | 100.0% | 0.00 | 0.00 | 0.72 | 0.000 | 0.000 | 0.628 |
|  | 072 | I | 100.0% | 100.0% | 0.0% | 0.0% | 0.0% | 100.0% | 100.0% | 0.00 | 0.50 | 0.50 | 0.000 | 0.505 | 0.505 |
|  | 086 | I | 91.7% | 0.0% | 0.0% | 100.0% | 0.0% | 0.0% | 100.0% | 0.00 | 0.00 | 0.48 | 0.000 | 0.000 | 0.425 |
|  | 087 | I | 75.0% | 94.8% | 0.0% | 0.0% | 0.0% | 100.0% | 100.0% | 0.00 | 0.42 | 0.42 | 0.000 | 0.281 | 0.281 |
|  | 088 | I | 100.0% | 100.0% | 100.0% | 100.0% | 100.0% | 100.0% | 100.0% | 1.00 | 1.00 | 1.00 | 1.000 | 1.000 | 1.000 |
|  | 089 | I | 0.0% | 0.0% | 0.0% | 100.0% | 0.0% | 0.0% | 100.0% | 0.00 | 0.00 | 0.25 | 0.000 | 0.000 | 0.258 |
|  | 090 | I | 100.0% | 100.0% | 100.0% | 0.0% | 100.0% | 100.0% | 100.0% | 0.75 | 0.75 | 0.75 | 0.753 | 0.753 | 0.753 |
|  | 091 | I | 100.0% | 100.0% | 2.2% | 100.0% | 0.0% | 100.0% | 100.0% | 0.00 | 0.76 | 0.76 | 0.000 | 0.753 | 0.753 |
|  | 092 | I | 0.0% | 0.0% | 93.5% | 0.0% | 0.0% | 0.0% | 100.0% | 0.00 | 0.00 | 0.23 | 0.000 | 0.000 | 0.193 |
|  | 093 | I | 98.3% | 0.0% | 100.0% | 88.0% | 0.0% | 0.0% | 100.0% | 0.00 | 0.00 | 0.72 | 0.000 | 0.000 | 0.628 |
|  | 130 | I | 5.0% | 100.0% | 0.0% | 0.0% | 0.0% | 100.0% | 0.0% | 0.00 | 0.26 | 0.00 | 0.000 | 0.258 | 0.000 |
|  | 131 | I | 0.0% | 100.0% | 0.0% | 0.0% | 0.0% | 100.0% | 0.0% | 0.00 | 0.25 | 0.00 | 0.000 | 0.258 | 0.000 |
|  | 132 | I | 90.0% | 0.0% | 0.0% | 96.0% | 0.0% | 0.0% | 100.0% | 0.00 | 0.00 | 0.47 | 0.000 | 0.000 | 0.371 |
|  | 135 | I | 0.0% | 87.9% | 0.0% | 0.0% | 0.0% | 100.0% | 0.0% | 0.00 | 0.22 | 0.00 | 0.000 | 0.151 | 0.000 |
|  | 137 | I | 98.3% | 0.0% | 100.0% | 0.0% | 0.0% | 0.0% | 100.0% | 0.00 | 0.00 | 0.50 | 0.000 | 0.000 | 0.486 |
|  | 098 | II | 0.0% | 100.0% | 0.0% | 0.0% | 0.0% | 100.0% | 0.0% | 0.00 | 0.25 | 0.00 | 0.000 | 0.258 | 0.000 |
|  | 165 | II | 28.3% | 0.0% | 0.0% | 0.0% | 0.0% | 0.0% | 100.0% | 0.00 | 0.00 | 0.07 | 0.000 | 0.000 | 0.017 |
